# Imaging drug uptake by bioorthogonal stimulated Raman scattering microscopy†
†Electronic supplementary information (ESI) available: Fig. S1–S11, Table S1, Scheme S1, synthetic procedures and spectra for all labels and Raman-labelled anisomycin derivatives, drug uptake avi files. See DOI: 10.1039/c7sc01837a. Primary data files can be found at http://dx.doi.org/10.7488/ds/2046


**DOI:** 10.1039/c7sc01837a

**Published:** 2017-05-24

**Authors:** William J. Tipping, Martin Lee, Alan Serrels, Valerie G. Brunton, Alison N. Hulme

**Affiliations:** a EaStCHEM School of Chemistry , The University of Edinburgh , Joseph Black Building, David Brewster Road , Edinburgh , EH9 3FJ , UK . Email: Alison.Hulme@ed.ac.uk; b Edinburgh Cancer Research Centre , Institute of Genetics and Molecular Medicine , The University of Edinburgh , Crewe Road South , Edinburgh , EH4 2XR , UK . Email: v.brunton@ed.ac.uk

## Abstract

Stimulated Raman scattering (SRS) microscopy in tandem with bioorthogonal Raman labelling strategies is set to revolutionise the direct visualisation of intracellular drug uptake. Rational evaluation of a series of Raman-active labels has allowed the identification of highly active labels which have minimal perturbation on the biological efficacy of the parent drug. Drug uptake has been correlated with markers of cellular composition and cell cycle status, and mapped across intracellular structures using dual-colour and multi-modal imaging. The minimal phototoxicity and low photobleaching associated with SRS microscopy has enabled real-time imaging in live cells. These studies demonstrate the potential for SRS microscopy in the drug development process.

## Abstract 2

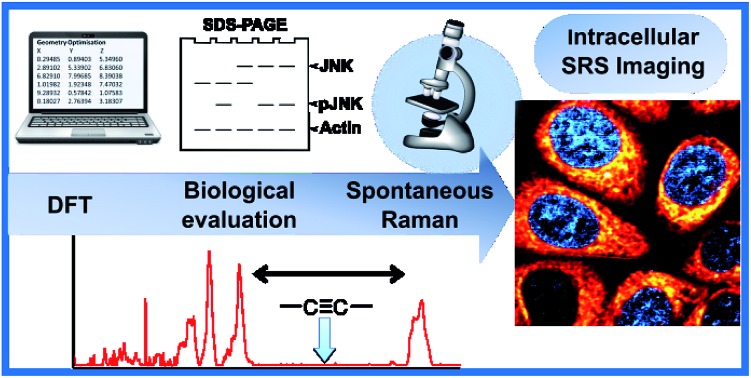
Stimulated Raman scattering (SRS) microscopy in tandem with bioorthogonal Raman labelling enables intracellular drug concentrations, distribution and therapeutic response to be measured in living cells.

## Introduction

Visualising the complex interplay between bioactive small molecules and an intricate network of cellular machinery represents a major challenge within chemical biology, medical sciences and pharmaceutical development.^1^–^4^ Improving pre-clinical modelling studies, through integrating advanced imaging techniques into the early stages of drug-discovery campaigns, may also help reduce the high attrition rates of clinical drug candidates.^5^ Consequently, techniques which enable the direct visualisation of drug molecules in a non-destructive manner, and without the use of bioorthogonal ligation or antibody detection, represent an attractive prospect for biomedical and pharmaceutical research.

Spontaneous Raman microscopy enables the detection of molecular vibrations and has been applied to the direct visualisation of cells and their contents,^6^ and used for *in vivo* cancer diagnosis.^7^ Advances in microscope design and the development of stimulated Raman scattering (SRS) microscopy have delivered improved sensitivity, spectral resolution and imaging speeds.^8^–^10^ SRS allows sample visualisation and quantification based upon bond-specific chemical contrast, at up-to video-rate acquisition speeds.^11^ It has been widely used to study high-concentration species within cells, including protein,^12^–^14^ lipids^15^,^16^ and DNA.^17^,^18^ SRS microscopy in tandem with the development of Raman labelling strategies looks set to play an important role as a new imaging modality to significantly enhance drug discovery and life-sciences research.^19^–^23^

**Fig. 1:**
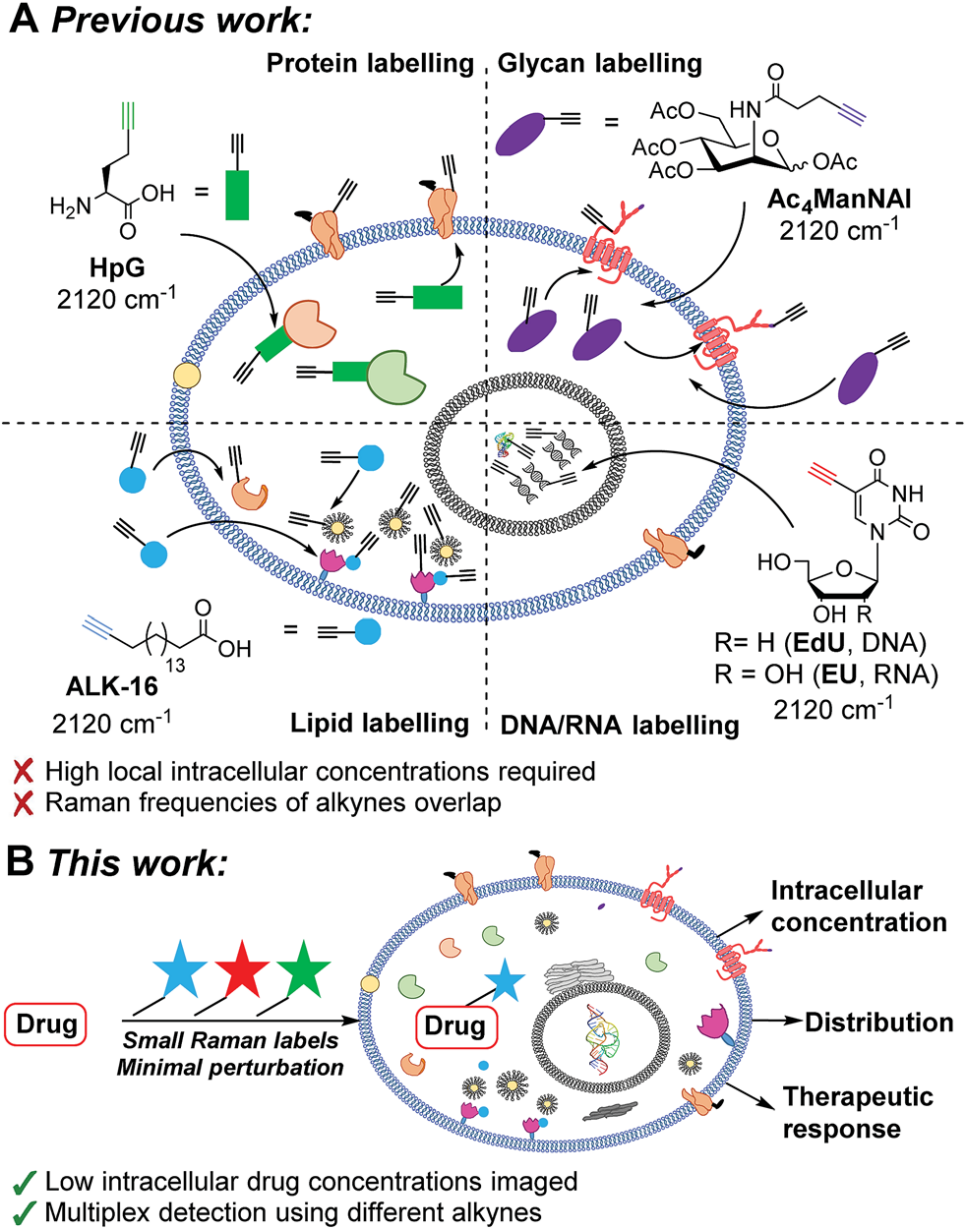
Bioorthogonal Raman imaging approaches. (A) Raman imaging through metabolic incorporation of alkynes. (B) Multicolour SRS drug imaging with bioorthogonal (bis)alkyne labels.

Spectroscopically bioorthogonal labelling strategies, including alkyne-tag Raman imaging, have been proposed as an efficient route to bio-molecule visualisation by Raman microscopy (Fig. 1).^24^ Alkynes, and a number of other functional groups, generate Raman signals within the cellular silent region (1800–2800 cm^–1^), making them particularly attractive as labels. A limited number of spectroscopically bioorthogonal Raman labelled species, typically with high intracellular concentrations, have been visualised by Raman and SRS microscopy.^25^–^29^ This includes **EdU** (Fig. 1A),^30^ an alkyne-containing thymidine analogue, and the clinical drug, erlotinib.^31^ However, despite the potential which this technique clearly demonstrates, a unified approach to the successful imaging of drugs within cells using SRS microscopy is yet to be established. An analytical approach based on label design, biological validation, confirmation of cellular uptake and successful SRS imaging would allow SRS microscopy to be fully exploited in the drug development process.

## Results and discussion

### Selection of Raman labels

Small molecule visualisation by Raman microscopy requires a large Raman scattering cross section with minimal perturbation on the structure and dynamics of the conjugated small molecule. Rational prediction of Raman activity could greatly accelerate the design of labels for use in biological systems, and help to overcome current detection limits for the intracellular visualisation of small molecules.^31^ Density functional theory (DFT) calculations at the B3LYP/6-31G(d) level of theory have been used to aid the identification of drug metabolites within cells,^31^ whilst *ab initio* calculations at the HF/6311G(d) level have been used to predict the polarizability of alkyne and aryl alkyne groups.^29^ These previous studies have shown that increased conjugation within a poly-yne chain is concomitant with enhanced polarizability and hence an increase in Raman scattering cross section.^32^–^34^ Experimentally, the introduction of aryl end-capping groups can also improve the stability and shelf-life of poly-ynes.^35^–^37^ We sought to evaluate computationally a range of nitrile and alkynyl labels, which are both small and produce intense Raman bands in the cellular silent region (1800–2800 cm^–1^),^24^,^29^ and to compare these results directly with experimentally determined values.

**Fig. 2:**
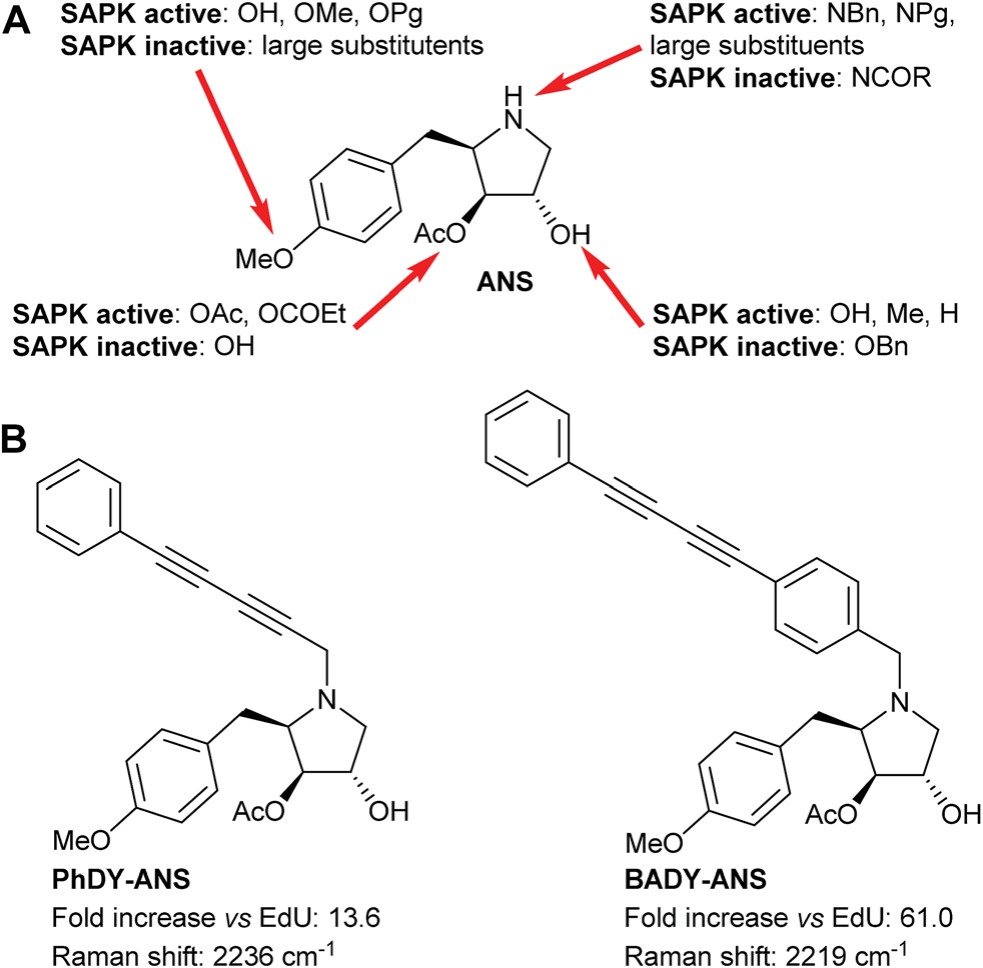
Functionalisation of the anisomycin scaffold. (A) Summary of the known SAR for anisomycin (**ANS**) in SAPK activation studies (Pg indicates a propargyl group). (B) Labelled anisomycin derivatives used in this study: *N*-5-phenyl-2,4-pentadiyn-1-yl anisomycin (**PhDY-ANS**) and *N*-4-(4-phenyl-1,3-butadiyn-1-yl)benzyl anisomycin (**BADY-ANS**). Fold-increase *vs.***EdU** represents the increase in Raman scattering activity of the Raman label compared to **EdU** (C≡C) predicted by DFT calculation. Raman shifts of the solid material measured using the corresponding ·TFA salt.

The bioactive small molecule anisomycin (**ANS**, Fig. 2) was identified as a suitable scaffold onto which spectroscopically bioorthogonal labels could be readily introduced with minimal functional perturbation,^38^–^40^ allowing direct comparison between predicted and experimental Raman activity (Fig. S1 and Table S1†). To model the labels, DFT calculations using the B3LYP hybrid exchange-correlation functional, with the 6-31G(d,p) double-zeta plus polarisation basis set, were used to predict the vibrational shift and Raman scattering activities (*I*_Ram_). For comparison, DFT calculations were also performed on **EdU**, which has previously been successfully imaged by SRS microscopy.^25^,^26^ As expected, these DFT calculations predicted an increase in the Raman scattering activity as the polarizability of the Raman label increased (Fig. S2A†). A series of labelled anisomycin derivatives were prepared to validate these predictions (Scheme S1†), and the spontaneous Raman spectra were recorded of each (Fig. S3†). The experimental results show a high level of consistency with DFT predictions (Fig. S2B, S4†) and allowed the identification of two lead compounds (**PhDY-ANS** and **BADY-ANS**, Fig. 2) for use in this study.

### Assessment of biological activity

Although the labels analysed by DFT generally have a much smaller *M*_W_ than traditionally used fluorophores and thus might be expected to have fewer effects on drug metabolism and pharmacokinetic properties (DMPK),^41^ they may still perturb the function of a drug under investigation. Initial biological screens for activity are typically conducted in the low (1–25 μM) to mid (25–100 μM) micromolar range,^42^ which correlates with the higher end of drug concentration ranges for plasma and extracellular fluid.^43^ Thus we sought to validate the biological efficacy of our labelled anisomycin derivatives at low micromolar concentrations.

**Fig. 3:**
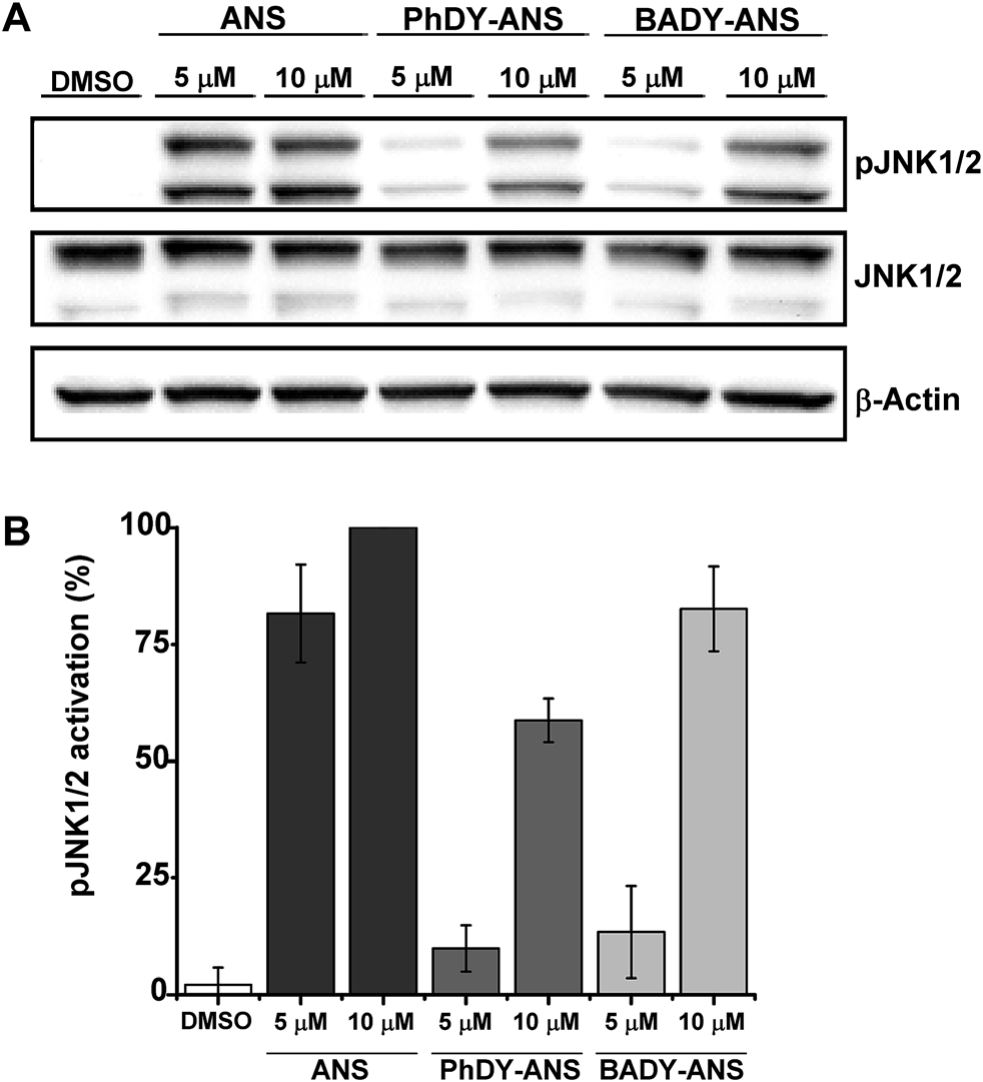
Effects of anisomycin and Raman-labelled anisomycin derivatives, **PhDY-ANS** and **BADY-ANS** on the phosphorylation of JNK1/2 isoforms in SKBR3 cells. (A) SKBR3 cells were exposed to DMSO (lane 1); anisomycin (5 μM, lane 2; 10 μM, lane 3); **PhDY-ANS** (5 μM, lane 4; 10 μM, lane 5) and **BADY-ANS** (5 μM, lane 6; 10 μM, lane 7). Western blot analysis was carried out with an antibody specific to phosphorylated JNK (pJNK1/2 at Thr183) and an antibody that recognises phosphorylated and unphosphorylated JNK1/2 equally well (JNK1/2). β-Actin was used as a loading control. (B) Quantification of the extent of JNK1/2 phosphorylation (pJNK1/2) in SKBR3 cells under the conditions described in (A). The pJNK1/2 activation levels in (A) were normalised to the pJNK1/2 activation observed with anisomycin (10 μM, 30 min) which is expressed as 100%. Error bars represent the standard deviation across *n* = 3 repeats.

Anisomycin is a potent inhibitor of protein synthesis and is known to activate the mitogen activated protein kinase pathways JNK/SAPK1 and p38/SAPK2 across a range of cell lines.^44^–^46^ Thus phosphorylation of JNK1/2 was used as a read-out of the biological efficacy of anisomycin and its Raman-labeled derivatives. Anisomycin has shown activity against a number of breast cancer cell lines^47^–^49^ and phosphorylation of JNK1/2 in mammalian SKBR3 breast cancer cells was therefore selected as a suitable model. Time-course studies showed that maximal activation of JNK1/2 in SKBR3 cells by anisomycin was obtained at low micromolar concentrations within 30 minutes (Fig. S5A†), and this activity was mirrored by all of the Raman-labelled derivatives (Fig. S5B†). Although **PhDY-ANS** and **BADY-ANS** gave reduced levels of activation at 5 μM they were both shown to be active at 10 μM (Fig. 3), indicating that they retain biological activity at the low micromolar concentrations typically used in screening assays.

### Raman spectral analysis and SRS microscopy of anisomycin derivatives

**Fig. 4:**
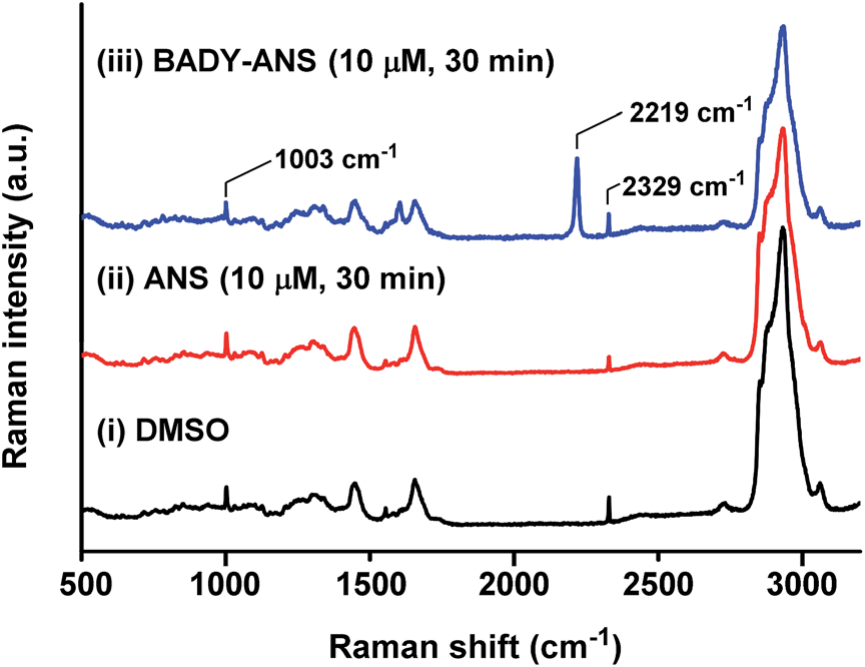
Raman spectroscopy of SKBR3 cells treated with **ANS** and **BADY-ANS**. Representative spontaneous Raman spectrum recorded of a single fixed SKBR3 cell treated with (i) DMSO; (ii) **ANS** (10 μM, 30 min) and (iii) **BADY-ANS** (10 μM, 30 min). Raman spectra were normalised to the intensity of the peak at 1003 cm^–1^ (phenylalanine ring breathing mode), scaled between 0–35 000 counts and offset for clarity. The following peaks have been annotated: 1003 cm^–1^ (phenylalanine ring breathing mode); 2219 cm^–1^ (C≡C, **BADY-ANS**); 2329 cm^–1^ (N_2_).^27^,^30^ Raman spectra were acquired at *λ*_ex_ = 532 nm for 10 s using a 50× objective.

Spontaneous Raman spectroscopy can be used to determine drug uptake, with studies on pelleted cells providing a useful initial screening platform.^50^ The local intracellular environment may affect the Raman vibrational frequency associated with bioorthogonal peaks; thus screening by spontaneous Raman spectroscopy enables rapid establishment of on- and off-target vibrational frequencies for subsequent SRS microscopy. SKBR3 cells were treated with the most Raman active derivative, **BADY-ANS** (10 μM, 30 min) and washed; analysis of a treated cell revealed a clear peak indicative of **BADY-ANS** at 2219 cm^–1^, which is absent in the DMSO and anisomycin treated controls (Fig. 4). This supports both the cellular uptake of **BADY-ANS**, and that there was negligible change in the bioorthogonal Raman vibrational shift relative to that of the solid material (*cf.* Table S1†).

**Fig. 5:**
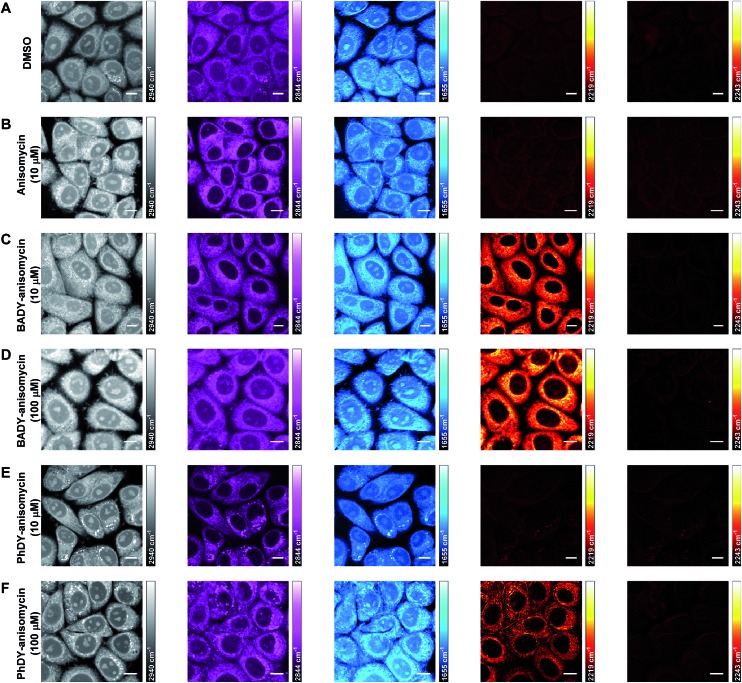
Multicolour SRS imaging of fixed SKBR3 cells treated with **ANS** and its derivatives. SKBR3 cells were treated with: (A) DMSO; (B) **ANS** (10 μM, 30 min); (C) **BADY-ANS** (10 μM, 30 min); (D) **BADY-ANS** (100 μM, 30 min); (E) **PhDY-ANS** (10 μM, 30 min) and (F) **PhDY-ANS** (100 μM, 30 min). Images were acquired in sequence at (i) 2940 cm^–1^ (CH_3_, proteins); (ii) 2844 cm^–1^ (CH_2_, lipids); (iii) 1655 cm^–1^ (amide-I, proteins); (iv) 2219 cm^–1^ (C≡C, **PhDY-**/**BADY-ANS**); 2243 cm^–1^ (cell-silent region). Images acquired at 1024 × 1024 pixels, 20 μs pixel dwell time with false colours applied to different detection wavenumbers. Scale bars: 10 μm.

Imaging using spontaneous Raman is time-consuming with typical acquisition times in excess of 30 minutes for a single cell-mapping experiment.^51^ SRS offers up-to video-rate acquisition speeds, and hence is preferred for cellular imaging.^11^ In this study, SRS microscopy was performed on a custom-built microscope,^52^ with optimum sensitivity achieved at speeds of 20 μs per pixel (which matches the longest time constant on the lock-in amplifier). This resulted in image acquisition times of less than 1 minute for a 1024 × 1024 frame, with pixel sizes ranging from 100 nm × 100 nm to 1 μm × 1 μm depending upon the field of view scanned. SKBR3 cells were treated with DMSO and **ANS** (10 μM, 30 min) and SRS images were acquired by tuning the frequency difference between the pump and Stokes lasers to be resonant with intracellular components including proteins (CH_3_, 2953 cm^–1^; amide I, 1655 cm^–1^)^53^ and lipids (CH_2_, 2844 cm^–1^).^54^ False colours were applied to individual Raman spectral frequencies to allow the differentiation of intracellular components (Fig. 5A and B). This process allows cellular registration without the need for any additional labelling.

SRS images can contain background signals from competing pump-probe processes such as cross-phase modulation, transient absorption and photothermal effects.^55^ Re-tuning the pump wavelength by a few nanometers allows off-resonance images to be acquired (at a difference of 10–20 cm^–1^ from the on-resonance image), which can be used to distinguish true SRS signals from these artefacts.^55^ Tuning to the spectroscopically bioorthogonal region of these cellular images (Fig. 5A and B) showed there was minimal SRS signal detected at either 2219 cm^–1^ (C≡C **BADY-ANS** on resonance) or 2243 cm^–1^ (cell-silent region), indicating that any signals detected in this region can be assigned to the Raman-labelled species with confidence. Subsequently SKBR3 cells were treated with **BADY-ANS** (10 μM, 30 min), washed and fixed for imaging. Tuning to the bioorthogonal region of the Raman spectrum enabled detection of **BADY-ANS** (alkyne, 2219 cm^–1^; off-resonance, 2243 cm^–1^) which distributes throughout the cytoplasm of the cell (Fig. 5C), and is especially pronounced in regions surrounding the nucleus.§
§This is consistent with the distribution observed by fluorescence microscopy upon incubation of HEK-293 cells with *N*-linked dansyl-anisomycin (109 μM, 30 min): see ref. 38. The off-resonance image at 2243 cm^–1^, where no Raman transition occurs, demonstrates the non-resonant background free nature of SRS microscopy. SKBR3 cells treated at higher **BADY-ANS** concentrations (100 μM, 30 min) showed a similar intracellular distribution of labelled drug (Fig. 5D) to those treated at the lower concentration. DFT calculations predicted that **PhDY-ANS** would have a reduced Raman scattering cross section (Table S1†) and when SKBR3 cells were treated with **PhDY-ANS** at lower concentrations (10 μM, 30 min) the labelled derivative could not be detected by SRS (Fig. 5E). However, when SKBR3 cells were treated with higher concentrations of **PhDY-ANS** (100 μM, 30 min) the expected cytoplasmic distribution of drug was observed (Fig. 5F). These data suggest that the higher Raman scattering intensity of the BADY label, as compared to PhDY, enables detection of the Raman-labelled drug at lower dosing concentrations.

Semi-quantitative determination of the absolute intracellular concentration of **BADY-ANS** was achieved using a dilution series in DMSO. Using the standard microscope set-up and an acquisition speed of 20 μs per pixel, a linear relationship between SRS contrast and concentration was obtained (Fig. S6†); with clear contrast between on-resonance and off-resonance signals for **BADY-ANS** at 500 μM solution concentrations. The determination of the absolute intracellular concentration of **BADY-ANS** is difficult due to variation in the signal intensities both within individual cells and across a cell population (Fig. 5). However, the images suggest that the local intracellular concentrations of labelled drug could be enriched by more than 50-fold from the dosing concentrations used (10 μM). These results highlight the potential that this method has for probing mechanistic aspects of cellular drug uptake.

### Multi-modal and dual-colour imaging in live cells

**Fig. 6:**
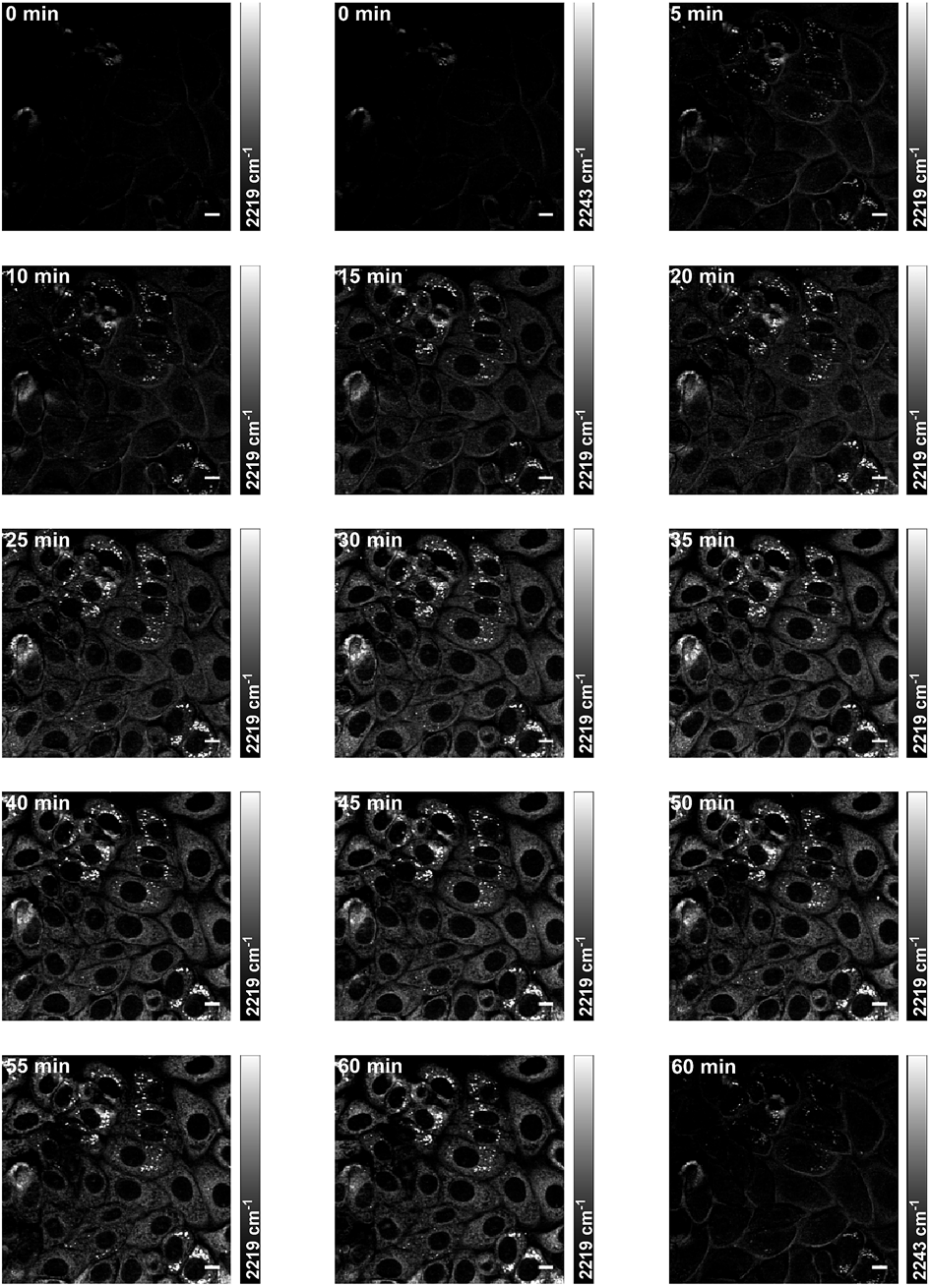
Time-lapse imaging of **BADY-ANS** uptake into live SKBR3 cells. SKBR3 cells were treated with **BADY-ANS** (10 μM at *t* = 0 min) and imaged at 2219 cm^–1^ (C≡C, **BADY-ANS**) every minute for 60 min. Off-resonance images were acquired at 2243 cm^–1^ (cell-silent region) at *t* = 0 min and *t* = 60 min. Images were acquired at 1024 × 1024 pixels, 20 μs pixel dwell time. Scale bars: 10 μm.

Live cell imaging of drug accumulation would enable studies correlating drug uptake and subcellular localisation with downstream phenotypic markers such as changes to cellular composition and cell cycle progression.^56^ To demonstrate the potential of SRS microscopy to visualise drug uptake and retention, time-resolved imaging of **BADY-ANS** uptake into live cells was performed. SKBR3 cells were treated with **BADY-ANS** (10 μM at *t* = 0 min), and an on-resonance SRS image (at 2219 cm^–1^) was acquired every minute at 20 μs per pixel, for 60 minutes (Fig. 6, ESI† Movie **BADY-ANS**). Intracellular **BADY-ANS** is detectable within 5 min suggesting rapid accumulation of the drug within cells, and it appears as locally concentrated bright spots within 10 min which persist through to 60 minutes.

Off-resonance images acquired at 0 and 60 minutes suggest that there is no localised sample damage due to prolonged image acquisition under these conditions (Fig. 6). Furthermore, the lack of any detectable signal (at 2219 cm^–1^) in time-lapse images of **ANS** uptake over 60 minutes at equivalent dosing levels (Fig. S7, ESI† Movie **ANS**) indicates that the increase in signal observed in Fig. 6 is a direct result of **BADY-ANS** uptake, rather than the emergence of any parasitic signals. The signal intensity afforded by the BADY label means that SRS images can be acquired at much faster acquisition times if needed, and at speeds which are unobtainable using conventional spontaneous Raman spectroscopy. Images of SKBR3 cells incubated with **BADY-ANS** at the higher dosing concentration (100 μM, 20 min) could be obtained in under 1 s using acquisition times of 2 μs per pixel (512 × 512 frame, Fig. S8†).¶
¶Acquisition speed limited by the microscope rather than signal intensity. These real-time images highlight the applicability of Raman-labels to drug uptake studies and demonstrate the minimal phototoxicity resulting from SRS imaging conditions. They also show that in contrast to fluorescence microscopy, where photostability can represent a major experimental challenge, Raman labels show minimal photobleaching.^57^

**Fig. 7:**
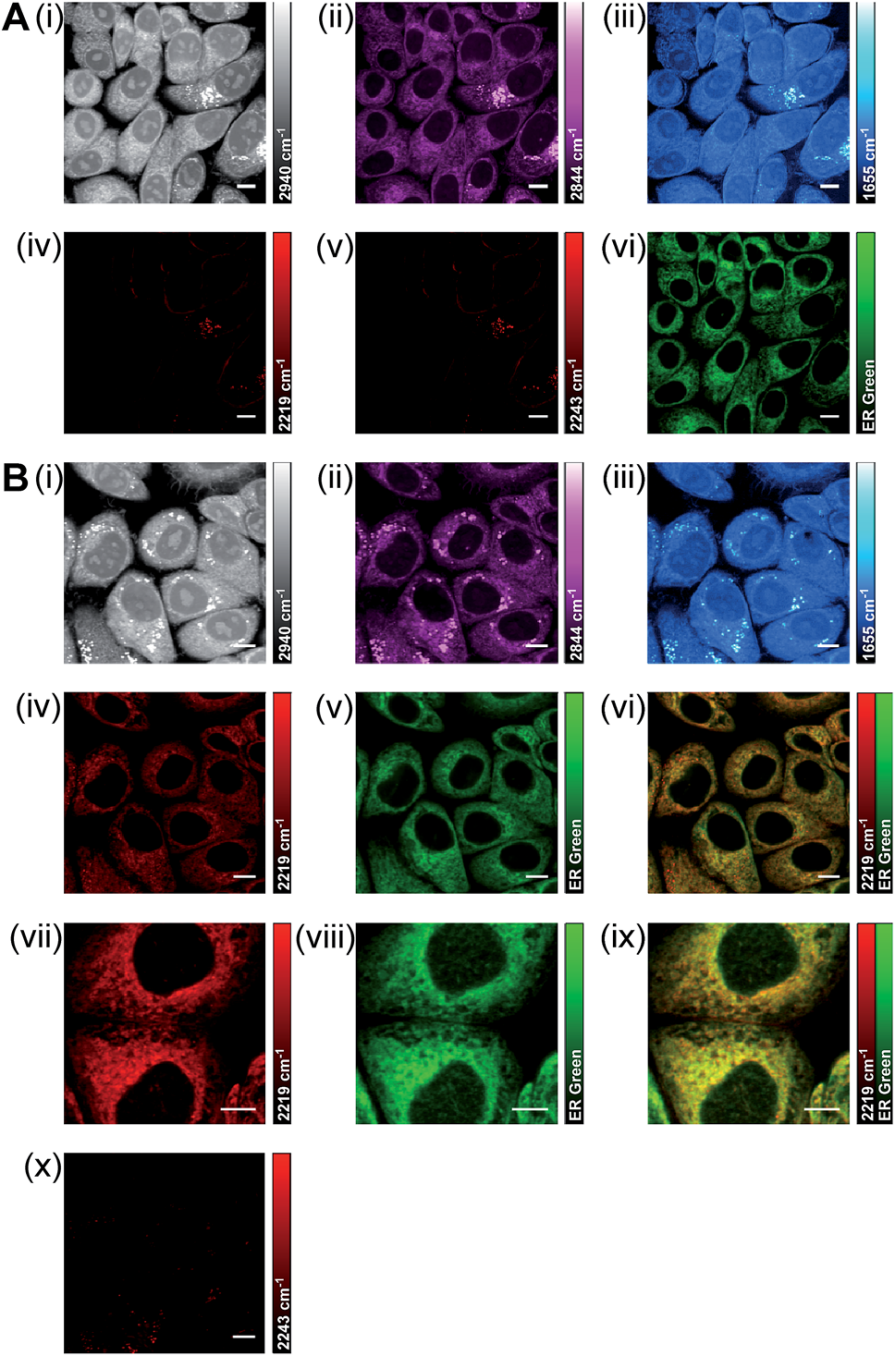
Multi-modal imaging to determine the intracellular location of **BADY-ANS**. (A) SKBR3 cells were treated with DMSO and ER Tracker Green (1 μM, 30 min) before washing and fixing. SRS images were acquired at (i) 2940 cm^–1^ (CH_3_, proteins); (ii) 2844 cm^–1^ (CH_2_, lipids); (iii) 1655 cm^–1^ (amide-I, proteins); (iv) 2219 cm^–1^ (C≡C, **BADY-ANS**); (v) 2243 cm^–1^ (cell-silent region); (vi) Two-photon fluorescence (TPF) image (ER Tracker Green). (B) SKBR3 cells were treated with **BADY-ANS** (10 μM, 30 min) and ER Tracker Green (1 μM, 30 min) before washing and fixing. SRS images were acquired at (i) 2940 cm^–1^ (CH_3_, proteins); (ii) 2844 cm^–1^ (CH_2_, lipids); (iii) 1655 cm^–1^ (amide-I, proteins); (iv) and (vii) 2219 cm^–1^ (C≡C, **BADY-ANS**); and (x) 2243 cm^–1^ (cell-silent region); (v) and (viii) TPF image (ER Tracker Green); (vi) merge of images acquired in (iv) and (v); (ix) merge of images acquired in (vii) and (viii). SRS images acquired at 1024 × 1024 pixels, 20 μs pixel dwell time with false colours applied to different detection wavenumbers. TPF images *λ*_ex_ = 860.8 nm and 1064 nm. Scale bars: 10 μm.

Using a dual-colour approach, an overlay of images acquired for lipid CH_2_ (2844 cm^–1^) and **BADY-ANS** (2219 cm^–1^) at 30 minutes (Fig. S9†) suggests that in some cells the drug is initially concentrated in lipid droplets; however, a very heterogeneous uptake is observed across the focal field. Using a multi-modal approach to investigate this further, SKBR3 cells were treated simultaneously with **BADY-ANS** (10 μM, 30 min) and ER-Tracker Green (1 μM, 30 min), a cell-permeable fluorescent stain selective for the endoplasmic reticulum (Fig. 7). In the control experiment, cells treated with the DMSO vehicle and ER Tracker green (Fig. 7A) showed no SRS signal at either 2219 cm^–1^ (C≡C **BADY-ANS** on-resonance) or 2243 cm^–1^ (cell-silent region), indicating that there is no interference in the SRS images arising from the presence of the fluorophore. In the **BADY-ANS** treated cells (Fig. 7B), the Raman-labelled drug is co-localised with ER-Tracker Green in the merged images, which is in agreement with its known binding to the 60S ribosomal subunit.^44^ These studies show that SRS microscopy allows rapid, non-destructive, high-resolution imaging of drug uptake in living cells which can be readily augmented using dual-colour and multi-modal approaches to follow accumulation into intracellular structures.

Image acquisition at wavenumbers resonant with intracellular components allows drug-induced changes in cellular composition to be analysed, which may help define the drug mechanism of action. The protein CH_3_ peak (2940 cm^–1^) has been used to distinguish between necrotic and healthy tissues and also for the detection of tumor margins.^58^,^59^ However, such changes in global protein content will not occur following acute drug administration. In contrast, the dynamic nature of lipid droplet assembly and disassembly,^60^,^61^ means that quantification of short term drug-induced changes in lipid droplets is possible. SKBR3 cells were treated (10 μM, 30 min) with either **ANS** or **BADY-ANS** and the lipid CH_2_ peak (2844 cm^–1^) was used to determine changes in the number of lipid droplets across the respective cell populations (Fig. S10†). There was a small reduction in the number of lipid droplets following treatment with **ANS** and **BADY-ANS** compared to DMSO controls, but further mechanistic studies will be required to determine the biological significance of these findings. That both **ANS** and **BADY-ANS** resulted in the same reduction in lipid droplet number provides further evidence that the BADY label does not alter the biological activity of anisomycin.

**Fig. 8:**
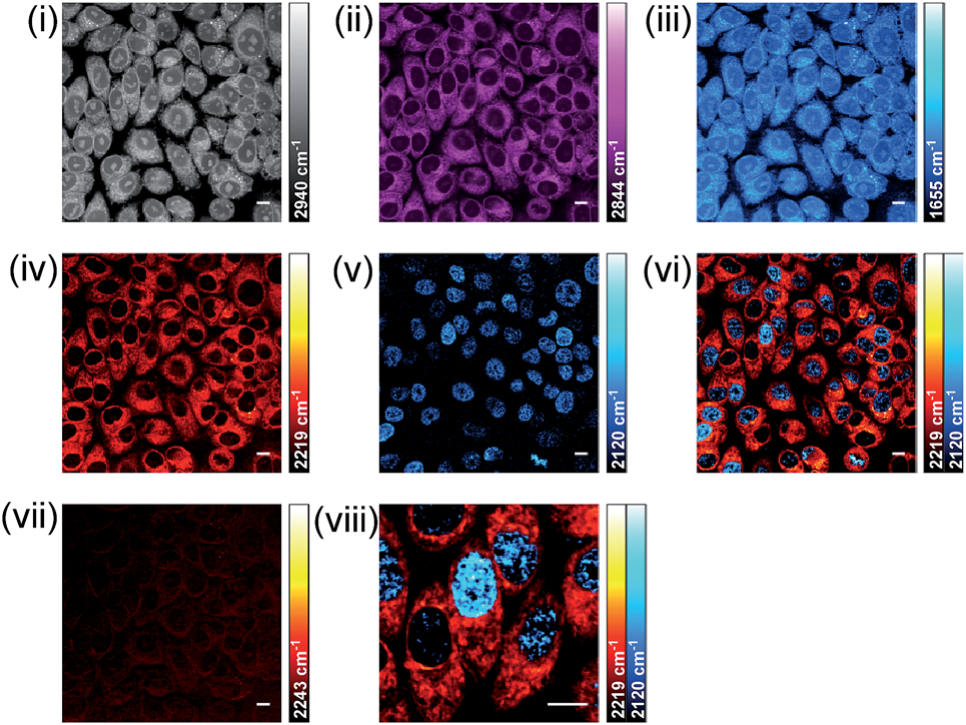
Dual-colour alkyne-label imaging by SRS microscopy. SKBR3 cells were treated with **EdU** (100 μM, 18 h) and **BADY-ANS** (10 μM, 30 min), washed and fixed prior to imaging. SRS images acquired at (i) 2940 cm^–1^ (CH_3_, proteins); (ii) 2844 cm^–1^ (CH_2_, lipids); (iii) 1655 cm^–1^ (amide-I, proteins); (iv) 2219 cm^–1^ (C≡C, **BADY-ANS**); (v) 2120 cm^–1^ (C≡C, **EdU**); (vi) overlay of images acquired in (iv) and (v); (vii) 2243 cm^–1^ (cell-silent region); (viii) expanded view of image presented in (vi). Images acquired at 1024 × 1024 pixels, 20 μs pixel dwell time with false colours applied to different detection wavenumbers. Scale bars: 10 μm.

As well as providing information about the spatial distribution of drug uptake, a dual-colour SRS approach might also be used to correlate drug uptake with markers of cell cycle progression. **EdU** (Fig. 1) is a thymidine analogue widely used as a probe for DNA synthesis in proliferating cells through the CuAAC conjugation of fluorophores;^62^ and its detection has recently been achieved by Raman microscopy.^30^ Detection of **EdU** and **BADY-ANS** would demonstrate the potential application of SRS microscopy to this field. SKBR3 cells were serum-starved to synchronise the cell population in the G0 phase of the cell cycle, then treated with **EdU** (100 μM, 18 h) in serum containing medium to stimulate DNA synthesis, before washing and treatment with **BADY-ANS** (10 μM, 30 min). Intracellular uptake of both molecules was confirmed by the presence of two peaks (at 2120 cm^–1^**EdU** and 2219 cm^–1^**BADY-ANS**) in the spontaneous Raman spectrum of a pellet of treated cells (Fig. S11†). Multicolour SRS microscopy allowed the detection of intracellular protein (CH_3_, 2953 cm^–1^; amide-I, 1655 cm^–1^), lipid (CH_2_, 2844 cm^–1^), **BADY-ANS** (C≡C, 2219 cm^–1^) and **EdU** (C≡C, 2120 cm^–1^) in a single cell (Fig. 8). Remarkably, despite the lower Raman scattering activity afforded by the bioorthogonal alkyne of **EdU** as predicted by DFT (Fig. S2A†), it is possible to detect it by SRS microscopy. This suggests that the high intra-nuclear concentration of **EdU** compensates for its lower Raman scattering activity, enabling its detection by SRS within the cell. Merging the image acquired for **BADY-ANS** (at 2219 cm^–1^) and **EdU** (at 2120 cm^–1^), allows simultaneous assessment of the intracellular drug concentration and cell cycle status (Fig. 8). This demonstrates the potential of this approach for identifying drug dependent changes in cell cycle progression at the single cell level, which is an important advance in the drug development process.

### Application to the investigation of combination therapies

The use of combination therapies is an area of increasing interest in drug discovery;^63^–^65^ assessment of the individual effects of drugs (often with differential rates of uptake) on cell populations in combined doses represents a major challenge.^66^ Here we demonstrate the power of dual-colour alkyne-label imaging by SRS microscopy using bespoke alkyne-derived Raman labels as a potential solution to this challenge. Accumulation of a drug within cells can range from a high local concentration, as with **EdU**, to a more diffuse distribution, as illustrated for **BADY-ANS**. Since the signal intensity achieved is directly related to this local concentration, this is an important consideration in the choice of label used for Raman imaging. Drugs with a more diffuse distribution thus require the use of a higher intensity label (BADY or PhDY) to enable effective imaging. Selecting two appropriate Raman labels based on DFT predictions (Table S1†) would enable rapid experimental design. While further label development could extend the current spectroscopic pallet to enable greater opportunities for multiplex detection in SRS imaging.

## Conclusions

The outstanding biocompatibility of SRS microscopy is set to revolutionise our ability to probe intracellular biological processes. This study describes the rational evaluation of a selection of spectroscopically bioorthogonal labels for SRS microscopy, allowing two functionally-active lead derivatives (**PhDY-ANS** and **BADY-ANS**) to be selected for study. The subcellular localisation of the most Raman active derivative, **BADY-ANS**, has been determined using dual-colour and multi-modal imaging approaches. The simultaneous detection of **BADY-ANS** together with either lipid droplets or **EdU**, demonstrates the potential of dual-colour SRS microscopy for identifying drug-dependent changes in cellular composition and cell cycle progression at the single cell level. Time-resolved detection of the uptake of **BADY-ANS** into live cells highlights the biocompatibility of this technique. On-going improvements to microscope design and optics, combined with the development of bespoke spectroscopically bioorthogonal labels, ensure that the future is bright for this imaging modality.

## Experimental

### Computational methods

The geometries of compounds evaluated in Table S1 and Fig. S2† were optimised at the B3LYP/6-31G(d,p) level of theory in the gas phase, using the Gaussian 09 programme. Vibrational frequencies and Raman scattering activities, obtained from the polarizability derivatives, were calculated analytically.

### Cell culture

Human breast cancer cell line SKBR3 was purchased from the American Type Culture Collection, and maintained in Dulbecco's modified Eagle's medium (DMEM, Sigma-Aldrich) supplemented with 2 mM l-glutamine, 1% penicillin-streptomycin and 10% foetal bovine serum (all Thermo Fisher Scientific) at 37 °C in a humidified atmosphere containing 5% CO_2_. For experiments using **EdU** (Thermo Fisher Scientific) cells grown under these normal conditions were synchronized in G0 by serum starvation, where the 10% FBS containing medium was removed and cells were cultured in medium containing 0.1% FBS for 24 h, prior to **EdU** treatment in 10% serum-containing DMEM.

### Western blotting

Cells were treated with **ANS** (Abcam) or Raman-labelled derivatives as indicated in the figure legend. Cells were washed with ice-cold PBS and subsequently lysed in RIPA buffer supplemented with cOmplete™ ULTRA protease inhibitor and PhosSTOP phosphatase inhibitor cocktails (Roche). Cleared lysates were resolved by SDS-PAGE. Primary antibodies used for Western blotting were as follows: phosphoJNK1/2 (Thr183/Tyr185; 1 : 1000), JNK1/2 (1 : 1000) and β-Actin (1 : 3000) (all Cell Signaling Technologies).

### Spontaneous Raman spectroscopy

The spontaneous Raman spectra were acquired using a confocal Raman spectrometer (inVia Raman microscope, Renishaw) at room temperature. A 297 mW (206 mW after objective) 785 nm diode laser or a 200 mW 532 nm laser excitation source was used to excite the sample through a 50×, N.A. 0.75 objective (Leica Biosystems). The recorded spectral range for grating 1200 g mm^–1^ was 100–3200 cm^–1^. The total data acquisition was performed during 60 s for spectra recorded at *λ*_ex_ = 785 nm or 10 s for spectra recorded at *λ*_ex_ = 532 nm using the WiRE software. All of the spectra acquired at *λ*_ex_ = 785 nm were background subtracted using a background correction algorithm as described previously,^67^ whilst those acquired at *λ*_ex_ = 532 nm are uncorrected.

### Two-photon fluorescence and stimulated Raman scattering (SRS) microscopy

Images were acquired using a custom-built multi-modal microscope setup. A picoEmerald (APE, Berlin, Germany) laser provided both a tunable pump laser (720–990 nm, 7 ps, 80 MHz repetition rate) and a spatially and temporally overlapped Stokes laser (1064 nm, 5–6 ps, 80 MHz repetition rate). The output beams were inserted into the scanning unit of an Olympus FV1000MPE microscope using a series of dielectric mirrors and a 2× lens based beam-expanding module. The resulting 2.4 mm beams were expanded by a further 3.6× lens within the microscope and directed into an Olympus XLPL25XWMP N.A. 1.05 objective lens using a short-pass 690 nm dichroic mirror (Olympus). The objective was under filled to achieve higher power transmissions through the microscope which were shown to be essential to detect the SRS signal. Backscattered emission signals from two-photon fluorescence was separated from any backscattered excitation light using a short-pass 690 nm dichroic mirror and IR cut filter (Olympus). A series of filters and dichroic mirrors were then used to deconvolve the different emission signals onto one of 4 available photo-multiplier tubes (PMT). ER-Tracker Green twophoton fluorescence signals were filtered using FF552-Di02, FF483/639-Di01 and FF510/84 (Semrock).

For SRS measurements, the Stokes beam was modulated with a 20 MHz EoM built into the picoEmerald. Forward scattered light was collected by a 20× Olympus XLUMPLFLN N.A. 1.00 objective lens and Stokes light was removed by filtering with an ET890/220m filter (Chroma). A telescope focused the light onto an APE silicon photodiode connected to an APE lock in amplifier with the time constant set to 20 μs except for fast acquisitions where it was set to 2 μs. The lock in amplifier signal was fed into an Olympus FV10-Analog unit.

Laser powers after the objective were measured up to 40–70 mW for the pump laser and up to 70 mW for the Stokes laser. All images were recorded at 512 × 512 or 1024 × 1024 pixels with a pixel dwell time between 2 and 20 μs, using FluoView FV10-ASW scanning software (Olympus).

### Sample preparation for spontaneous Raman spectroscopy

SKBR3 cells were treated with **BADY-ANS** (10 μM, 30 min), **ANS** (10 μM, 30 min) or DMSO (1 : 1000, 30 min) and then washed with PBS prior to fixing with formaldehyde (3.7% in PBS, 10 min, 37 °C). The cells were washed with PBS (2×) prior to analysis of a single cell by spontaneous Raman spectroscopy at *λ*_ex_ = 532 nm. For analysis of **EdU** incorporation into newly-synthesised DNA, SKBR3 cells were serum starved for 24 h prior to treatment with **EdU** (100 μM, 18 h) in serum-containing DMEM followed by treatment with **BADY-ANS** (100 μM, 20 min), before washing with PBS prior to trypsinisation and diluted with DMEM. The resulting cell suspension was centrifuged at 175*g* and the cell pellet washed with DMEM/PBS (1/1 v/v; 10 mL) and re-centrifuged (2×). The cell pellet was transferred to CaF_2_ coverslips for analysis by spontaneous Raman spectroscopy using *λ*_ex_ = 785 nm.

### Sample preparation for SRS microscopy

For all SRS imaging experiments, SKBR3 cells were first seeded into Fluorodish Cell Culture Dishes (World Precision Instruments) with a density of 1 × 10^5^ cells per dish with 2 mL DMEM culture medium for 20 h. Details of the dosing concentrations and dosing times are provided in the figure legends. Where indicated, cells were fixed with formaldehyde (3.7% in PBS, 10 min, 37 °C) and washed with PBS (3 × 2 mL) before imaging. For multi-modal imaging experiments with ER-Tracker, SKBR3 cells were treated with ER-Tracker Green (BODIPY® TR Glibenclamide, Thermo Fisher Scientific) (1 μM, 30 min) and **BADY-ANS** (10 μM, 30 min), washed with PBS (3 × 2 mL), fixed (3.7% formaldehyde in PBS, 2 min, 37 °C), washed with PBS (3 × 2 mL; 5 min) and imaged by SRS microscopy and two-photon microscopy. For analysis of **EdU** incorporation into newly synthesised DNA, SKBR3 cells were serum starved for 24 h, prior to treatment with **EdU** (100 μM, 18 h) in serum-containing DMEM followed by treatment with **BADY-ANS** (10 μM, 30 min) and fixed.

### Image analysis

False colour assignments, overlays and scale bars were added to images using ImageJ. Consistent brightness settings were used throughout. For the analysis of lipid droplets: a z-stack across a typical field of view (∼40 cells, 25× objective lens) was acquired for each treatment condition and a maximum intensity projection was created (*n* = 9 for each condition). The number of lipid droplets >1 μm were counted using threshold analysis with the droplet_counter.jar application on ImageJ. For real-time imaging: frames were acquired at minute intervals and were compiled using ImageJ using consistent brightness settings throughout. Movies are supplied as .avi files in the ESI.†

## Supplementary Material

Supplementary movie

Supplementary movie

Supplementary information
