# Supplementary material for: Imaging drug uptake by bioorthogonal stimulated Raman scattering microscopy
Source: Chem Sci. 2017 May 24;8(8):5606–15. doi: 10.1039/c7sc01837a (PMC6103005; doi:10.1039/c7sc01837a)
Supplement: Supplementary file 3 [file SC-008-C7SC01837A-s003.pdf]

Electronic Supplementary Material (ESI) for Chemical Science.
This journal is © The Royal Society of Chemistry 2017

Imaging Drug Uptake by Bioorthogonal Stimulated Raman Scattering Microscopy

W. J. Tipping,[a,b] M. Lee,[b] A. Serrels,[b,c] V. G. Brunton[b,]* and A. N. Hulme[a,]*

[a.] EaStCHEM School of Chemistry, The University of Edinburgh, Joseph Black Building, David Brewster Road, Edinburgh, EH9 3FJ, U.K.
[b.] Edinburgh Cancer Research Centre, Institute of Genetics and Molecular Medicine, The University of Edinburgh, Crewe Road South, Edinburgh, EH4 2XR, U.K.
[c.] Current address: Queens Medical Research Institute, The University of Edinburgh, 47 Little France Crescent, Edinburgh, EH16 4TJ, U.K.

Supplementary Figures, Supplementary Materials and Synthetic Procedures

Table of Contents

Supplementary Figures S1- S11 and Table S1....1

General Methods 9

Preparative and Analytical RP-HPLC Conditions 9

Scheme S1 and Synthetic Procedures 10

References 20

^{1}H and ^{13}C NMR NMR Spectra 21

Raman labels ....21 – 33

Anisomycin analogues ....34 – 49

DFT Optimised Geometries for compounds 50

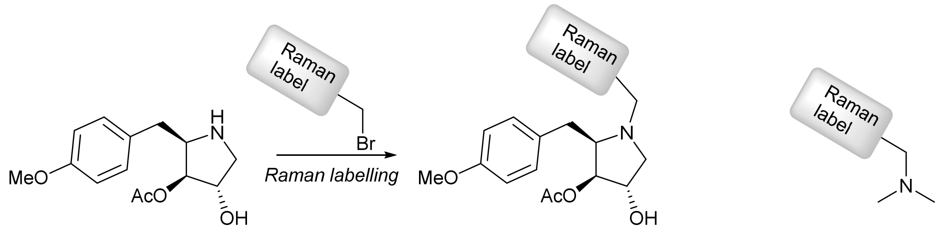

ANS **2b-i** **3b-i**

Figure S1 *N*-pyrrolidine labelled anisomycin derivatives (**2b-i**) used in the experimental determination of vibrational shifts and Raman scattering intensities. (**2h** = **PhDY-ANS**; **2i** = **BADY-ANS**) Dimethylamine counterparts (**3b-i**) used to as truncated models to enable rapid DFT calculations of vibrational shifts and Raman scattering activities (I_{Ram}).

Table S1 Anisomycin derivatives and their dimethylamine counterparts used in DFT studies.

Raman Label	R	Compound	Raman shift (cm^{-1}) [a]
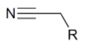	*N*-labelled anisomycin	**2b**	2234
	Me_2N	**3b**	-
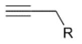	*N*-labelled anisomycin	**2c**	2136
	Me_2N	**3c**	-
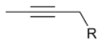	*N*-labelled anisomycin	**2d**	2249
	Me_2N	**3d**	-
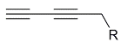	*N*-labelled anisomycin	**2e**	2239
	Me_2N	**3e**	-
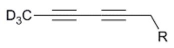	*N*-labelled anisomycin	**2f**	2260[b]
	Me_2N	**3f**	-
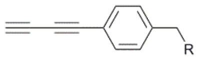	*N*-labelled anisomycin	**2g**	N.D.[c]
	Me_2N	**3g**	-
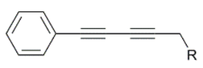	*N*-labelled anisomycin	**2h** (**PhDY-ANS**)	2236[d]
	Me_2N	**3h**	-
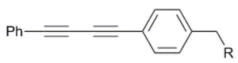	*N*-labelled anisomycin	**2i** (**BADY-ANS**)	2219
	Me_2N	**3i**	-

[a] Values presented for the solid material in the bioorthogonal region 1800 – 2800 cm^{-1}. [b] The bioorthogonal Raman shift due to the CD_3 group is of much lower intensity and was not used in this study. [c] Compound **2g** was found to be unstable to laser irradiation. [d] Intracellular shift 2219 cm^{-1} determined by SRS imaging.

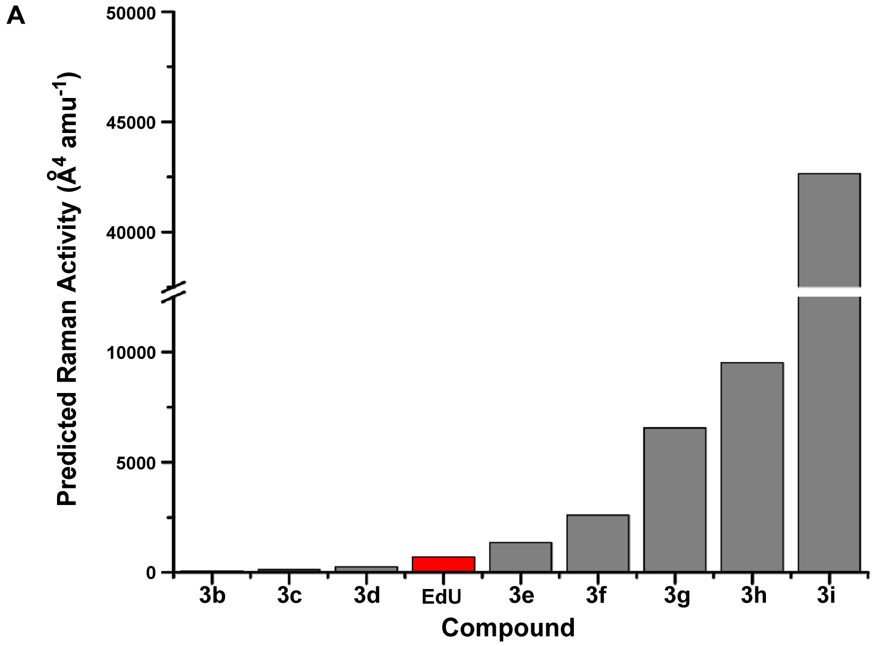

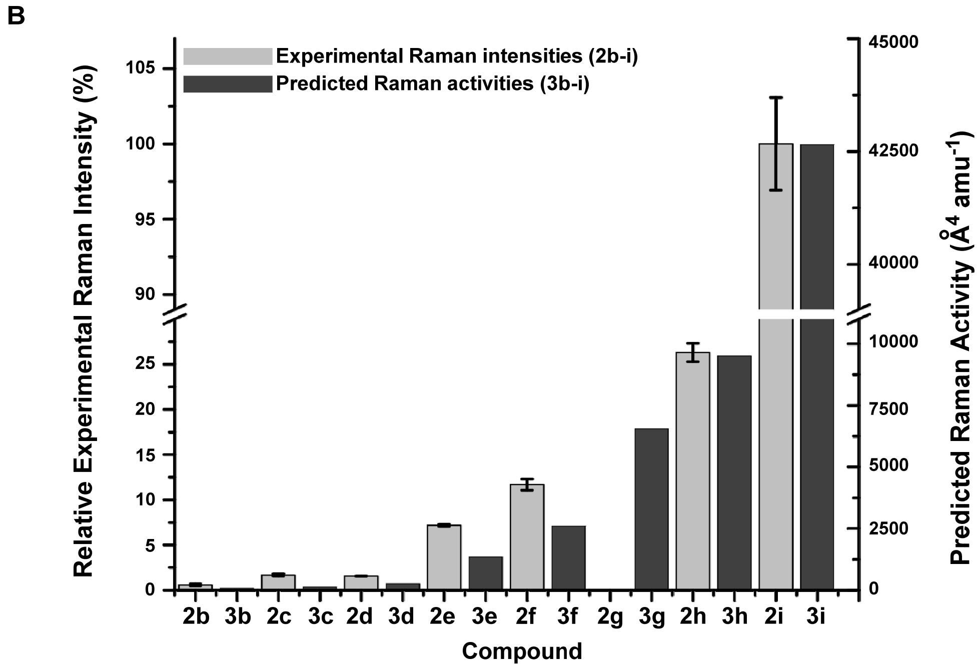

Figure S2 Raman activities anisomycin analogues and their dimethylamine counterparts. **A** Predicted Raman activity of dimethylamine adducts **3b-i**, and EdU **1**. DFT predicted Raman scattering activity (I_{ram}) of the peak maximum between 2100 – 2250 cm^{-1} [gas phase intensities calculated at B3LYP/6-31G(d,p)]. EdU is highlighted in red for clarity. **B** Comparison of the DFT-predicted Raman activities and experimental spontaneous Raman scattering intensities for peaks in the region 2100 – 2250 cm^{-1}. DFT gas phase activities (I_{Ram}) for dimethylamine adducts **3b-i** calculated at the B3LYP/6-31G(d,p) level. Relative spontaneous Raman scattering intensities for the solid anisomycin analogues **2b-i** normalised to the peak area$_{(819\ cm^{-1})}$ for each analogue and expressed as a percentage of the BADY-ANS peak; mean of ten replicates with error bars ± S.D. (**2h** = **PhDY-ANS**; **2i** = **BADY-ANS**)

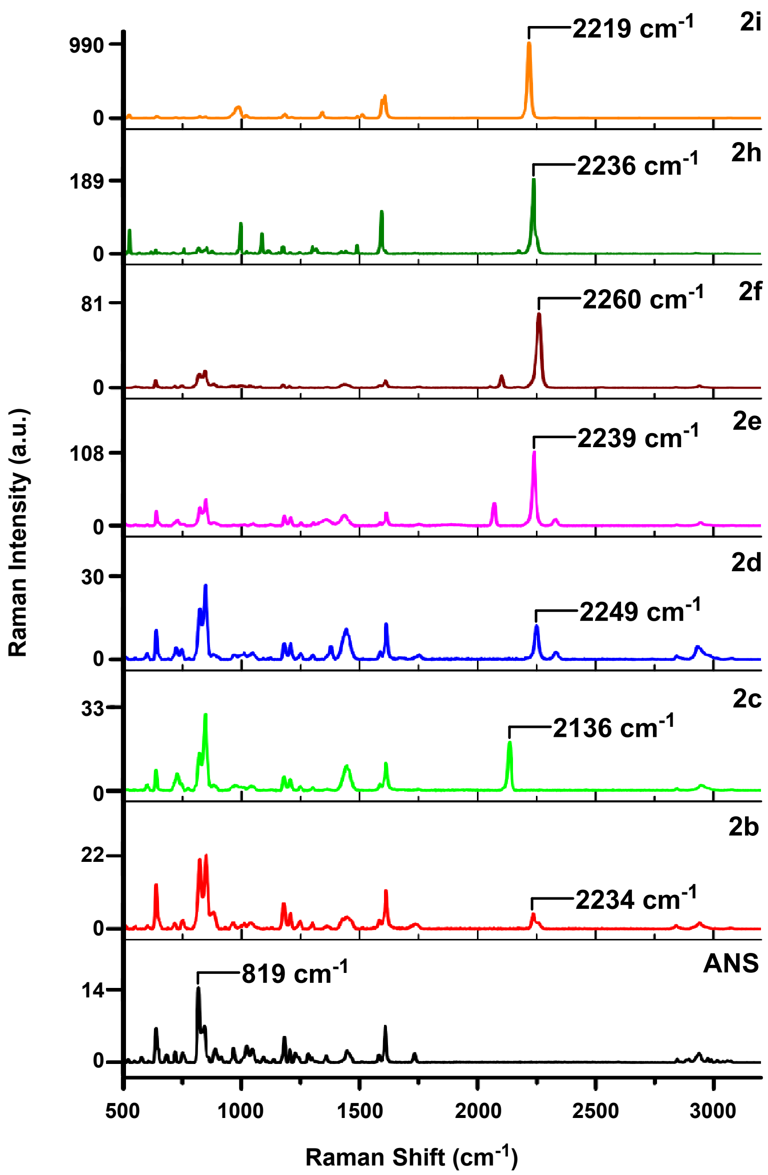

Figure S3 Spontaneous Raman spectroscopy of anisomycin **ANS** and analogues **2b-f**, **2h** and **2i** as solid material. Spectra normalised to the anisomycin peak at 819 cm^{-1} present in each sample, and offset for clarity. (**2h** = **PhDY-ANS**; **2i** = **BADY-ANS**)

Note: compound **2g** is unstable upon laser irradiation, and therefore no Raman spectrum is presented.

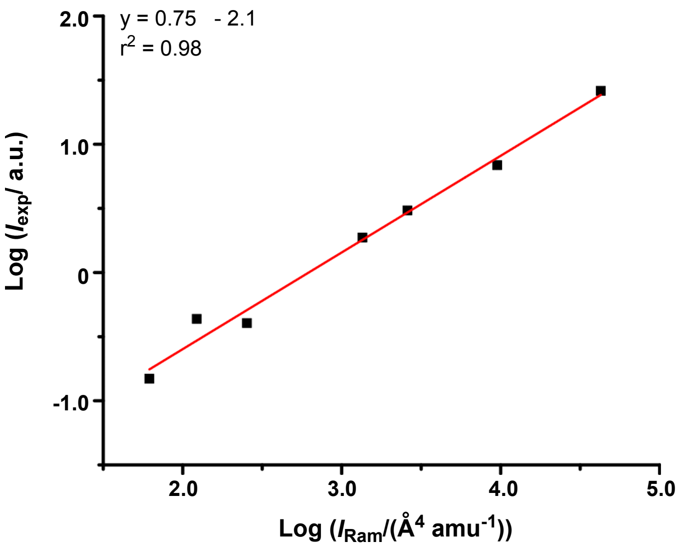

Figure S4 Correlation of the predicted Raman scattering activities calculated by Gaussian (I_{ram}), with experimental Raman intensities measured by spontaneous Raman scattering (I_{exp}). Due to the large dynamic ranges, these are plotted as their logarithms.

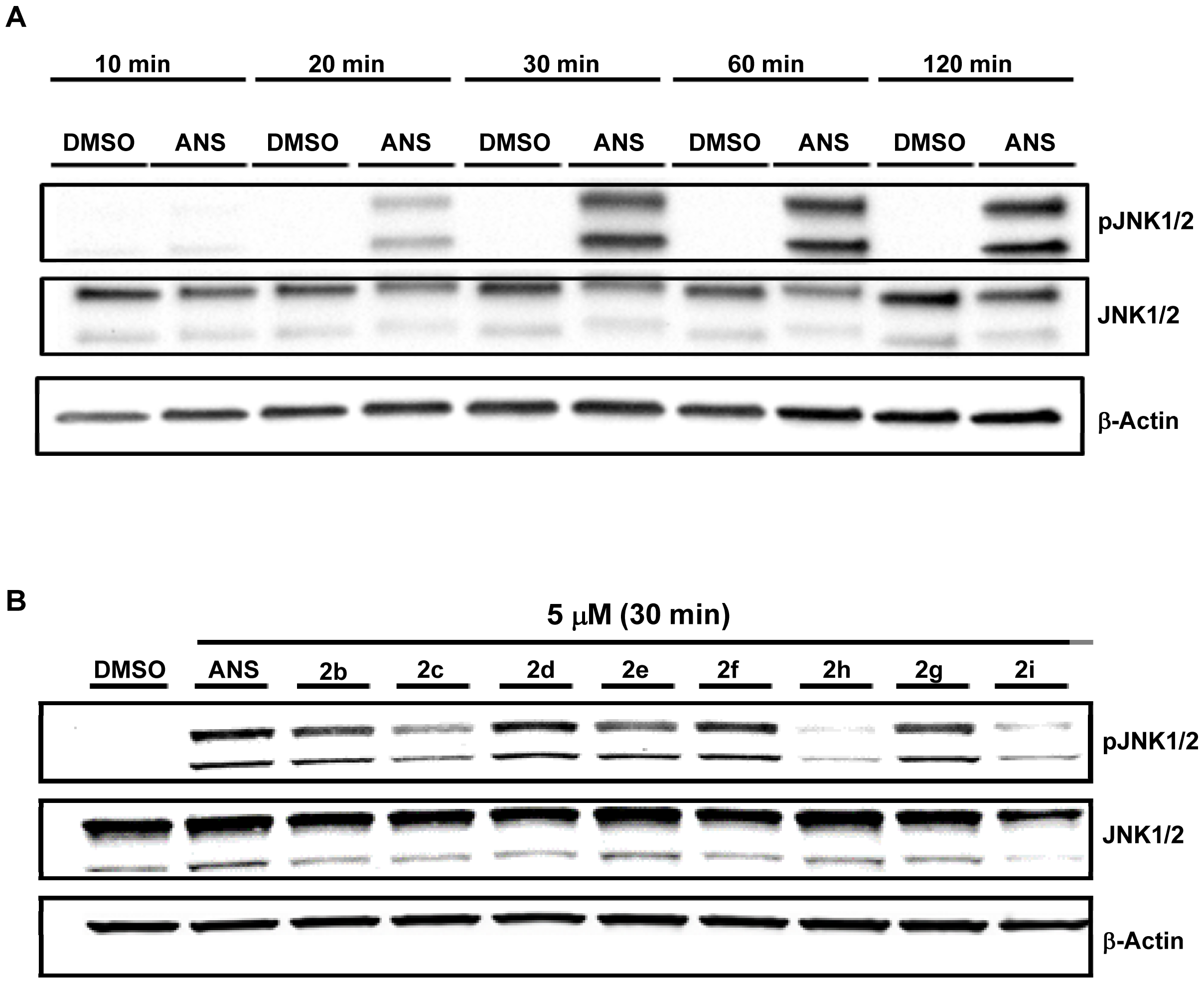

Figure S5 Effect of anisomycin **ANS** and analogues **2b-i** on the phosphorylation of JNK1/2 isoforms in SKBR3 cells. (**A**) Cells were exposed to either DMSO or anisomycin ANS (10 μM) at selected timepoints; (**B**) Cells were exposed to DMSO (lane 1), and 5 μM concentrations of anisomycin **ANS** (lane 2) and analogues **2b-i** (lanes 3-10) for 30 min. Western blot analysis was carried out with antibodies to phosphorylated JNK (pJNK1/2) and JNK1/2. β-actin was used as a loading control. (**2h** = **PhDY-ANS**; **2i** = **BADY-ANS**)

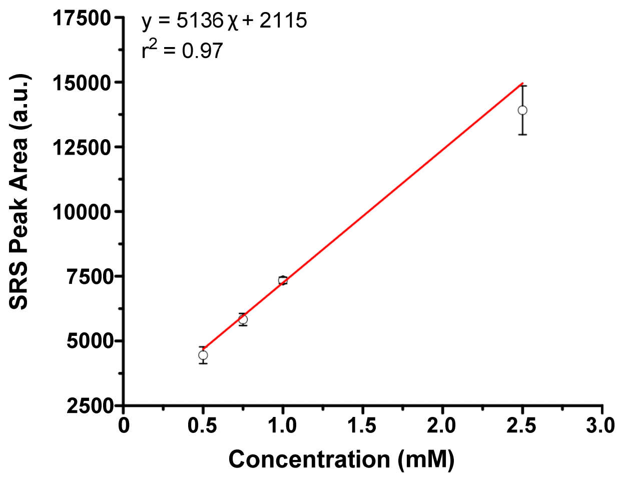

Figure S6 Correlation between SRS intensity measured at 2219 cm^{-1} (**BADY-ANS** on-resonance) and **BADY-ANS** concentration as DMSO stock solutions. Average pixel intensities were calculated using ImageJ software. Images were acquired at 512 × 512 pixels with a 20 µs pixel dwell time. Data represented as mean of 6 replicates with error bars ± SD.

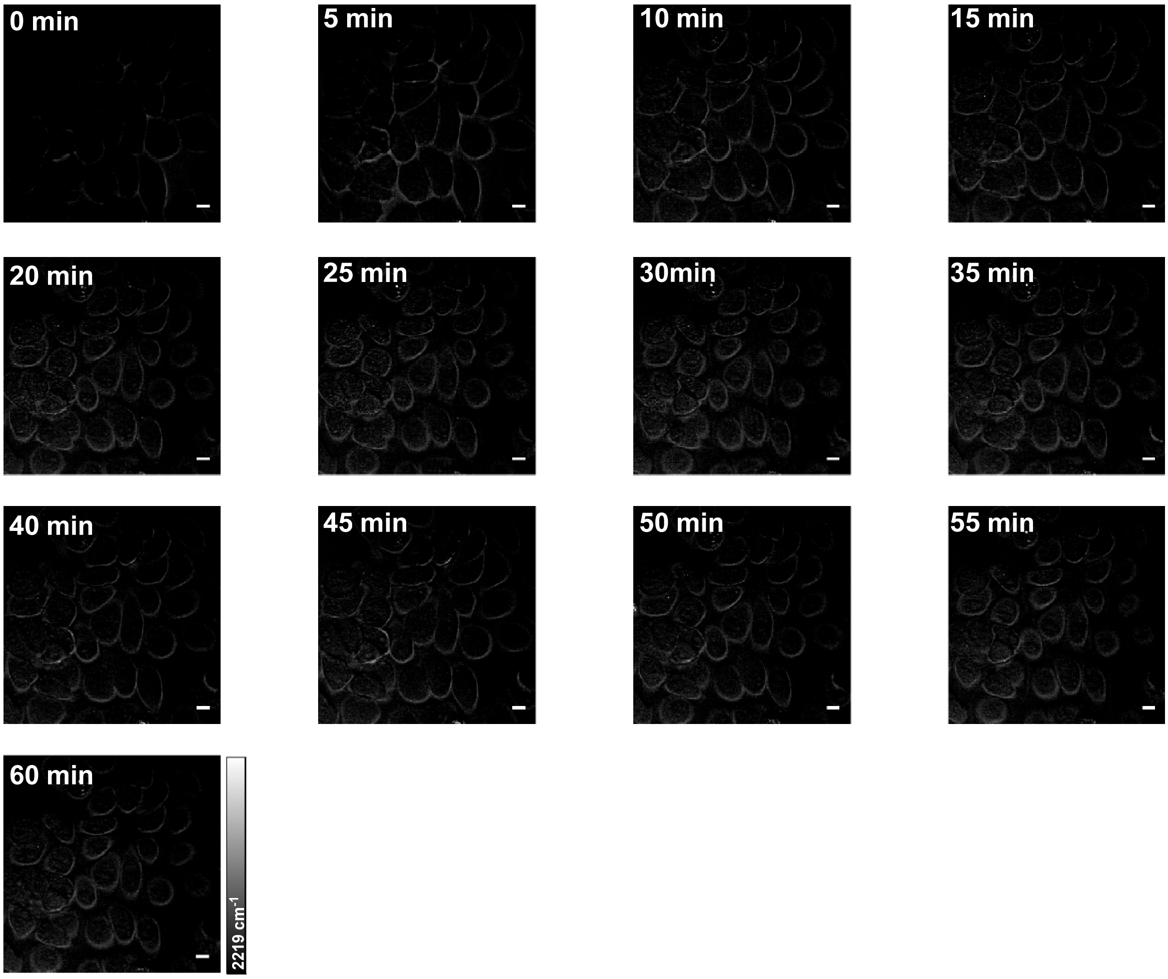

Figure S7 Time-lapse imaging of anisomycin **ANS** uptake into live SKBR3 cells. SKBR3 cells were treated with **ANS** (10 µM at t = 0 min) and imaged at 2219 cm^{-1} (C≡C, **BADY-ANS**) every minute for 60 mins. Images were acquired at 1024 × 1024 pixels, 20 µs pixel dwell time. Scale bars: 10 µm.

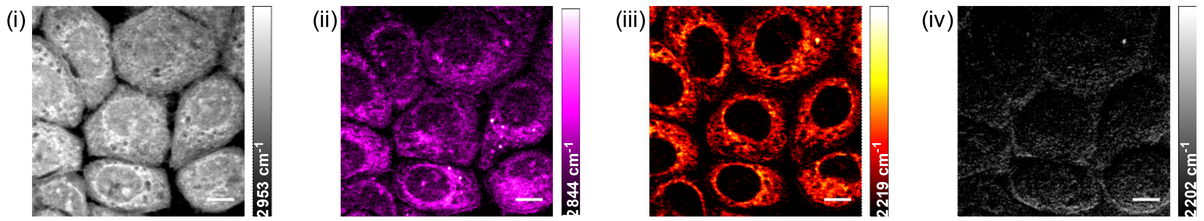

Figure S8. Fast-acquisition SRS images of fixed SKBR3 cells treated with **BADY-ANS**. SKBR3 cells treated with **BADY-ANS** (100 μM, 20 mins) and images acquired at (i) 2953 cm^{-1} (CH_3, proteins); (ii) 2844 cm^{-1} (CH_2, lipids); (iii) 2219 cm^{-1} (C≡C, **BADY-ANS**); and (iv) 2202 cm^{-1} (cell silent region). Images acquired at 512 × 512 pixels, 2 μs pixel dwell time, false colour for images applied to different detection wavenumbers. Scale bars: 10 μm.

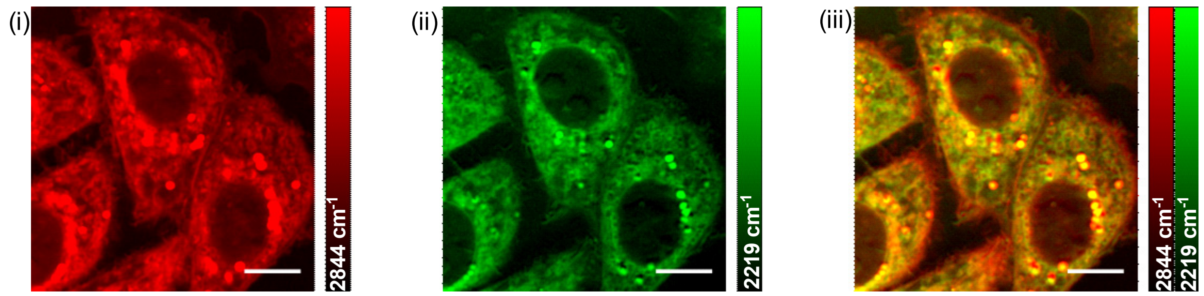

Figure S9 Multi-colour SRS imaging of fixed SKBR3 cells treated with **BADY-ANS**. SKBR3 cells were treated with **BADY-ANS** (10 μM, 30 min) and SRS images acquired at (i) 2844 cm^{-1} (CH_2, lipids) and (ii) 2219 cm^{-1} (C≡C, **BADY-ANS**) and (iii) overlay of (i) and (ii) showing that **BADY-ANS** is initially concentrated in lipid droplets in some of the cells. Images acquired at 1024 × 1024 pixels, 20 μs pixel dwell time, false colour for images applied to different detection wavenumbers. Scale bars: 10 μm.

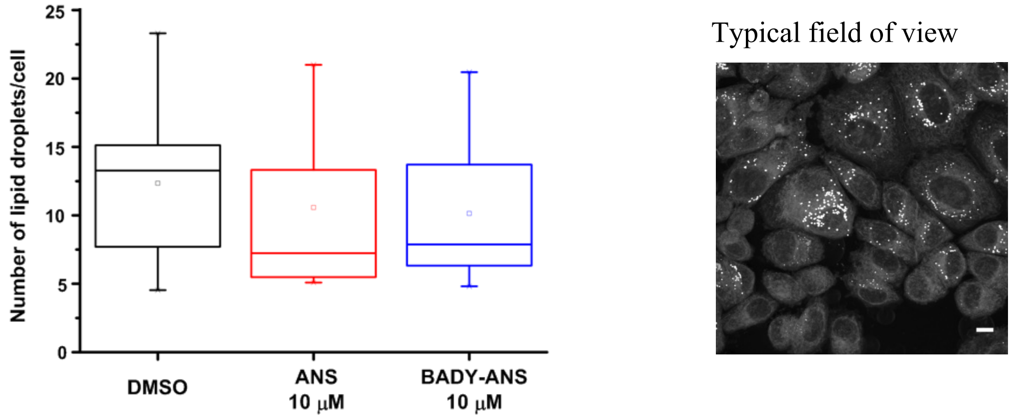

Figure S10 Quantification of the number of lipid droplets present in SKBR3 cells following treatment with (i) DMSO; (ii) **ANS** (10 μM, 30 min) and (iii) **BADY-ANS** (10 μM, 30 min). SRS images were acquired at 2844 cm^{-1} (CH_2, lipids) across a typical field of view (~40 cells, 20× objective lens, n = 9 repeats), and the number of lipid droplets >1 μm were counted using ImageJ. Inset: maximum intensity Z-projection for a typical field of view following acquisition of a Z-stack of SRS images at 2844 cm^{-1}. Scale bar: 10 μm. See Materials and Methods for further details. The average number of lipid droplets under each treatment is reported across n = 9 repeats.

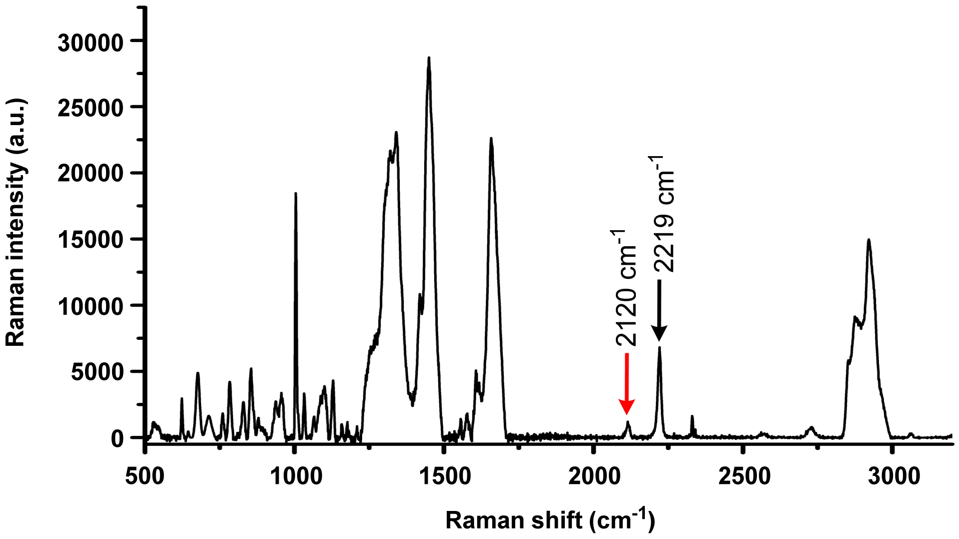

Figure S11 Dual colour alkyne-label imaging by spontaneous Raman spectrosocpy. Spontaneous Raman spectrum of SKBR3 cells treated with **EdU** (100 µM, 18 h) and **BADY-ANS** (100 µM, 20 min). Peak at 2120 cm^{-1} indicative of **EdU** (red) and peak at 2219 cm^{-1} indicative of **BADY-ANS** (black). Raman spectrum acquired at λ_{ex} = 785 nm for 60 s using a 50× objective.

Movie BADY-ANS

Live SKBR3 cells were treated with **BADY-ANS** (10 µM) at t=0 min and SRS images were acquired at 2219 cm^{-1} (C≡C, **BADY-ANS**) every minute for 60 minutes. The individual frames were compiled on ImageJ as an image stack to generate the movie. Scale bars: 50 µm.

Movie ANS

Live SKBR3 cells were treated with **ANS** (10 µM) at t=0 min and SRS images were acquired at 2219 cm^{-1} (C≡C, **BADY-ANS**) every minute for 60 minutes. The individual frames were compiled on ImageJ as an image stack to generate the movie. Scale bars: 50 µm.

General Methods

All non-aqueous reactions were carried out under an atmosphere of nitrogen or argon using oven-dried glassware that was cooled in a desiccator prior to use. Unless otherwise noted, starting materials and reagents were obtained from commercial suppliers and were used without further purification. Toluene, THF, CH_2Cl_2, and Et_2O were dried and purified by passage through activated alumina columns using a Glass Contour Solvent Purification System. Triethylamine was distilled from calcium hydride and stored over activated 4 Å molecular sieves under an argon atmosphere. Anhydrous DMF was purchased from Acros Organics. Saturated aqueous solutions of inorganic salts are represented as (volume, sat aq.). Nuclear magnetic resonance (**NMR**) spectra were recorded at ambient temperature (298 K, unless otherwise stated) on a Bruker AVA400, AVA500 or AVA600 spectrometer running at 400, 500, or 600 MHz (^{1}H spectra) or 101, 126, 151 Hz (^{13}C spectra), respectively. Chemical shifts (δ values) are reported in parts-per-million (ppm) relative to tetramethylsilane (^{1}H and ^{13}C spectra; $\delta_{TMS} = 0$) and are calibrated to the residual solvent peak. ^{1}H NMR data are reported as follows: chemical shift, relative intensity, multiplicity (s = singlet, d = doublet, t = triplet, q = quartet, qn = quintet, m = multiplet, br = broad), coupling constants (J value, Hz), and interpretation. ^{13}C NMR data are reported as follows: chemical shift, relative intensity and assignment (Q = quaternary, CH = methane, CH_2 = methylene, CH_3 = methyl). Infra-red spectra (**IR**) were recorded neat on Shimadzu IRAffinity-1. The value of peaks at maximum absorbance (ν_{max}) are quoted in wavenumbers (cm^{-1}). Mass spectra were obtained by electrospray (ESI) on a Bruker microTOF II mass spectrometer, or by electron ionisation (EI) on a Kratos MS50TC mass spectrometer. Mass-to-charge ratios (***m/z***) of all parent (molecular) ions ($[M]^{+/-}$) and their intensities are reported, followed by (major) fragment or adduct ions and their intensities. Melting points (**mp**) were determined on a Gallenkamp Electrothermal Melting Point apparatus and are uncorrected the temperature range. R_f values (**R_f**) were recorded using Merck Silicagel 60 F254 aluminium backed plates. Flash chromatography was carried out using Merck Kieselgel 60 (Merck 9385) under positive pressure. Eluent compositions are quoted as v/v ratios. R_t values were recorded by analytical reverse phase HPLC analysis using a Waters 600E (100 µL) gradient pump using a 717plus autosampler and a Waters 996 PDA equipped with a Phenomenex Luna C18(2), 5 µm, 250 x 4.6 mm column at a flow rate of 1 mL min^{-1}. Preparative reverse phase HPLC was performed using a Waters 600 (225 µL) system using a Waters 486 tuneable absorbance detector recording at 254 nm equipped with a Phenomonex Luna C18(2), 5 µm, 250 x 21.2 mm column at a flow rate of 21.2 mL min^{-1}.

Preparative RP HPLC Method A

Flow Rate: 21.2 mL min^{-1}	λ: 275 nm	
Time (min)	% H_2O + 0.1% TFA	% MeCN + 0.1% TFA
0.0	80	20
20	60	40
50	60	40
55	5	95
65	5	95

Analytical RP HPLC Method B

Flow Rate: 1.0 mL min^{-1}	λ: 275 nm	
Time (min)	% H_2O + 0.1% TFA	% MeCN + 0.1% TFA
0	80	20
20	60	40
25	5	95
35	5	95
40	80	20
50	80	20

Analytical RP HPLC Method C

Flow Rate: 1.0 mL min^{-1}	λ: 275 nm	
Time (min)	% H_2O + 0.1% TFA	% MeCN + 0.1% TFA
0.0	95	5
30	5	95
35	5	95
40	95	5
50	95	5

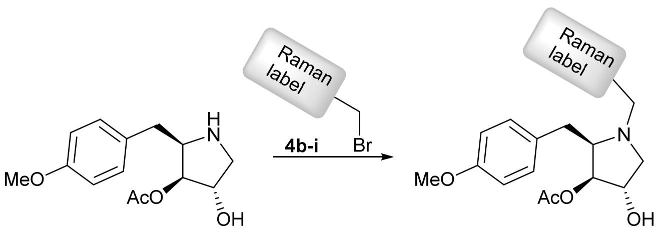

ANS **2b-i**

Scheme S1 Synthesis of anisomycin analogues. Reagents and Conditions: **ANS** (60 µmol), **4b-i** (60 µmol), K_2CO_3, DMF, 9 h, 71-95%.[37] Labels **4e** and **4g** were reacted as their alkyne protected-counterparts **4e-TMS** and **4g-TMS** which were deprotected *in situ*.

Synthesis of labels 4e-i

5-Trimethylsilyl-2,4-pentadiyn-1-ol, S1

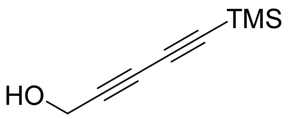

A solution of 1,4-bis(trimethylsilyl)butadiyne (486 mg, 2.50 mmol) and LiBr (271 mg, 3.13 mmol) in anhydrous Et_2O (25 mL) at -10 °C, was stirred for 10 min in darkness. MeLi (1.95 mL, 3.13 mmol; 1.6 M in Et_2O) was added dropwise and the mixture was stirred at -10 °C for 15 min, warmed to rt and stirred for a further 2 h in darkness. The mixture was cooled to 0 °C, and a suspension of paraformaldehyde (225 mg, 7.50 mmol) in dry Et_2O (30 mL) was added slowly, the mixture was warmed to rt and stirred for 18 h in darkness. The mixture was washed with NH_4Cl (50 mL; sat. aq.), $NaHCO_3$ (50 mL; sat. aq.), and brine (50 mL). The combined aqueous extracts were extracted with Et_2O (3 × 50 mL). The combined organic extracts were dried ($MgSO_4$), filtered, and concentrated *in vacuo*. The crude material was purified using flash column chromatography (Hexane:EtOAc, 5:1) to afford the product as a pale golden oil (199 mg, 52%). $\mathbf{R}_f$ (Hexane:EtOAc, 5:1) = 0.19; **IR** (neat, cm^{-1}) 3323 (OH), 2224 (C≡C), 2108 (C≡C); **^{1}H NMR** (CD_3OD, 500 MHz) δ 4.26 (2H, s, CH_2); 0.20 (9H, s, 3CH_3); **^{13}C NMR** (CD_3OD, 126 MHz) 87.2 (1C, Q), 85.5 (1C, Q), 76.5 (1C, Q), 68.7 (1C, Q), 49.6 (1C, CH_2), -1.9 (3C, 3CH_3); ***m/z*** (EI) 152.1 ($[M]^+$, 22%), 137.0 (100), 109.0 (17), 77.0 (10), 75.0 (31), 63 (3); **HRMS** (EI) calcd. for $C_8H_{12}OSi$ $[M]^+$ 152.0652, found 152.0647. ^{1}H and ^{13}C NMR spectroscopic data were in good agreement with the literature.[1]

5-Bromo-1-trimethylsilyl-1,3-pentadiyne, 4e-TMS

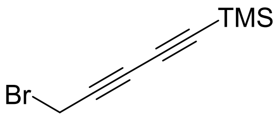

A solution of 5-trimethylsilyl-2,4-pentadiyn-1-ol **S1** (59.8 mg, 400 µmol) in anhydrous Et_2O (1.0 mL) at 0 °C, was treated successively with pyridine (2.5 µL, 32 µmol) and PBr_3 (15.2 µL, 160 µmol). The reaction mixture was warmed to rt and stirred for 18 h in darkness. The mixture was diluted with Et_2O (30 mL) and washed with Na_2CO_3 (15 mL; sat. aq.). The aqueous layer was separated and extracted with Et_2O (3 × 30 mL). The combined organic extracts were dried ($MgSO_4$), filtered and concentrated *in vacuo*. The crude material was purified using flash column chromatography (Hexane) to afford the product as a pale yellow oil (68 mg, 79%). $\mathbf{R}_f$ (Hexane) = 0.74; **IR** (neat, cm^{-1}) 2358 (C≡C), 2112 (C≡C); **^{1}H NMR** (CD_3OD, 500 MHz) δ 4.15 (2H, s, CH_2); 0.22 (9H, s, 3CH_3); **^{13}C NMR** (CD_3OD, 126 MHz) δ 87.8 (1C, Q), 86.6 (1C, Q), 72.6 (1C, Q), 70.1 (1C, Q), 13.0 (1C, CH_2), -2.0 (3C, 3CH_3); ***m/z*** (EI) 216.0 ($[^{81}BrM]^+$, 42%), 214.0 ($[^{79}BrM]^+$, 40), 201.0 (100), 199.0 (97), 172.9 (34), 170.9 (26), 135.1 (44), 64.1 (78); **HRMS** (EI) calcd. for $C_8H_{11}BrSi$ $[^{79}BrM]^+$ 213.9808, found 213.9807. ^{1}H and ^{13}C NMR spectroscopic data are in good agreement with the literature.[2]

[5-$^2H_3$]-1-Trimethylsilyl-1,3-pentadiyne, S2

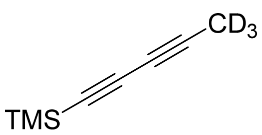

A solution of 1,4-bis(trimethylsilyl)butadiyne (972 mg, 5.00 mmol) in anhydrous THF (10 mL) was treated with MeLi•LiBr complex (3.78 mL, 5.67 mmol, 1.5 N in Et_2O), and the resultant mixture stirred at rt for 5 h in darkness. The reaction mixture was cooled to -78 °C, and CD_3I (335 µL, 5.38 mmol) was added dropwise. The mixture was stirred at -78 °C for 1 h, and slowly warmed to rt over 12 h. The reaction mixture was cooled to 0 °C, and slowly quenched with NH_4Cl (20 mL; sat. aq.). The aqueous mixture was then extracted with Et_2O (3 × 15 mL), and the combined organic

extracts were washed with water (3 × 15 mL) and brine (15 mL). The resultant organic mixture was dried ($MgSO_4$), filtered and concentrated *in vacuo*. The crude material was purified using flash column chromatography (Hexane) to give the product as a pale yellow oil (400 mg, 57%). **R_f**(Hexane) = 0.82; **IR** (neat, cm^{-1}) 2266 (C≡C), 2230 (C≡C), 2110 (CD); **^{1}H NMR** (CD_3OD, 500 MHz) δ 0.18 (9H, s, 3C*H_3*); **^{13}C NMR** (CD_3OD, 126 MHz) δ 88.4 (1C, Q), 80.8 (1C, Q), 75.1 (1C, Q), 64.0 (1C, Q), 1.6 (1C, sept, *J* 20.9 Hz, *C*D_3), -1.7 (3C, 3 × CH_3); ***m/z*** (EI) 139.0 ($[M]^+$, 39%), 124.0 (100); **HRMS** (EI) calcd. for $C_8H_9D_3Si$ $[M]^+$ 139.0891, found 139.0889.

[6-$^2H_3$]-2,4-Hexadiyn-1-ol, S3

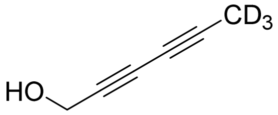

A solution of [5-$^2H_3$]-1-trimethylsilyl-1,3-pentadiyne **S2** (139 mg, 1.00 mmol) in anhydrous THF (10 mL) was cooled to -10 °C, and treated with MeLi•LiBr complex (830 µL, 1.25 mmol, 1.5 N in Et_2O). The mixture was stirred at -10 °C for 15 min, warmed to rt and stirred for an additional 2 h. After complete desilylation, the mixture was cooled to -10 °C, and a suspension of paraformaldehyde (60.9 mg, 2.03 mmol) in anhydrous THF (3.0 mL) was added. The mixture was warmed to rt and stirred for 18 h. The reaction was quenched by the slow addition of NH_4Cl (20 mL; sat. aq.), and the organic layer was washed with $NaHCO_3$ (20 mL; sat. aq.) and brine (20 mL). The combined aqueous layers were extracted with Et_2O (3 × 30 mL), and the combined organic extracts were dried ($MgSO_4$), filtered and concentrated *in vacuo*. The crude material was purified by flash column chromatography (Hexane:EtOAc, 10:1 → Hexane:EtOAc, 4:1) to afford the product as pale yellow needles (95 mg, 98%). **R_f** (Hexane:EtOAc, 4:1) = 0.39; **IR** (neat, cm^{-1}) 3329 (OH), 2259 (C≡C); **^{1}H NMR** (CD_3OD, 500 MHz) δ 4.21 (2H, s, C*H_2*); **^{13}C NMR** (CD_3OD, 126 MHz) δ 75.8 (1C, Q), 73.1 (1C, Q), 69.1 (1C, Q), 63.2 (1C, Q), 49.6 (1C, CH_2), 1.5 (1C, sept., *J* 20.7 Hz, CD_3); ***m/z*** (EI) 97.0 ($[M]^+$, 100%), 80.0 (60), 77.9 (73), 69.0 (72), 68.0 (46), 62.9 (60), 52.9 (39); **HRMS** (EI) calcd. for $C_6H_3D_3O$ $[M]^+$ 97.0602, found 97.0598.

[6-$^2H_3$]-1-Bromo-2,4-hexadiyne, 4f

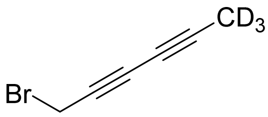

A solution of [6-$^2H_3$]-2,4-hexadiyn-1-ol **S3** (27.2 mg, 280 µmol) in anhydrous Et_2O (1.0 mL) at 0 °C, was treated successively with pyridine (1.9 µL, 24 µmol) and PBr_3 (10.5 µL, 110 µmol). The reaction mixture was warmed to rt and stirred for 18 h in darkness. The mixture was diluted with Et_2O (20 mL) and washed with Na_2CO_3 (10 mL; sat. aq.). The aqueous layer was separated and extracted with Et_2O (3 × 25 mL). The combined organic extracts were dried ($MgSO_4$), filtered and concentrated *in vacuo*. The crude material was purified by flash column chromatography (Hexane) to afford the product as a colourless oil (36 mg, 80%). **R_f**(Hexane) = 0.85; **IR** (neat, cm^{-1}) 2257 (C≡C); **^{1}H NMR** (CD_3OD, 600 MHz) δ 4.10 (2H, s, C*H_2*Br); **^{13}C NMR** (CD_3OD, 151 MHz) δ 78.0 (1C, Q), 70.8 (1C, Q), 69.4 (1C, Q), 62.9 (1C, Q), 13.6 (1C, CH_2), 1.7 (1C, sept. *J* 20.1 Hz, CD_3); ***m/z*** (EI) 161.0 ($[^{81}BrM]^+$, 27%), 159.0 ($[^{79}BrM]^+$, 28), 80.1 (100), 78.1 (58), 63.0 (62) **HRMS** (EI) calcd. for $C_6H_2D_3Br$ $[^{79}BrM]^+$ 158.9757, found 158.9762.

4-Iodobenzyl alcohol, S4

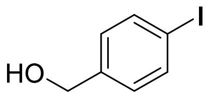

A solution of 4-iodobenzoic acid (1.44 g, 5.81 mmol) in anhydrous THF (12 mL) was treated with BH_3•THF (12.0 mL, 12.0 mmol, 1 N in THF), and the reaction stirred at rt for 4 h. The reaction was quenched by the careful addition of HCl (30 mL, 2 N aq.) and the resulting aqueous mixture extracted with CH_2Cl_2 (3 × 45 mL). The combined organic extracts were washed with $NaHCO_3$ (2 × 25 mL; sat. aq.) and brine (2 × 25 mL), dried ($MgSO_4$), filtered and concentrated *in vacuo*. The crude material was purified by flash column chromatography (Hexane:EtOAc, 10:1) to afford the product as a white solid (1.35 g, 99%). $\mathbf{R}_f$ (CH_2Cl_2) = 0.42; **mp** 66 – 68 °C, lit. 68 – 70 °C; **IR** (neat, cm^{-1}) 3294 (OH), 1584 (C=C), 789 (C-I); **^{1}H NMR** ($CDCl_3$, 500 MHz) δ 7.72 (2H, d, *J* 8.3 Hz, 2 × Ar*H*), 7.14 (2H, d, *J* 8.3 Hz, 2 × Ar*H*), 4.68 (2H, s, CH_2), 1.72 (1H, s, O*H*); **^{13}C NMR** ($CDCl_3$, 126 MHz) δ 140.5 (1C, Q), 137.6 (2C, CH), 128.8 (2C, CH), 93.0 (1C, Q), 64.7 (1C, CH_2); ***m/z*** (EI) 234.0 ($[M]^+$, 100%), 107.1 (28) **HRMS** (EI) calcd. for C_7H_7OI $[M]^+$ 233.9536, found 233.9536. ^{1}H and ^{13}C NMR spectroscopic data are in good agreement with the literature.[3]

1-Trimethylsilyl-1,3-butadiyne, S5

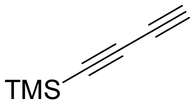

A solution of 1,4-bis(trimethylsilyl)butadiyne (972 mg, 5.00 mmol) in anhydrous Et_2O (10 mL) at 0 °C was treated with MeLi•LiBr complex (5.0 mL, 7.50 mmol, 1.5 N in Et_2O). The reaction mixture was warmed to rt, and stirred for 5 h. The reaction was quenched by the addition of NH_4Cl (10 mL, sat. aq.), and the resulting mixture extracted with Et_2O (3 × 20 mL). The combined organic extracts were washed with water (20 mL) and brine (20 mL), dried ($MgSO_4$), filtered and concentrated at ambient conditions overnight. The product was found to be volatile and unstable when concentrating to dryness; approximate concentration was determined by ^{1}H NMR (0.16 N in Et_2O). $\mathbf{R}_f$ (Hexane) = 0.98; **^{1}H NMR** ($CDCl_3$, 500 MHz) δ 2.13 (1H, s, *H*C≡CC≡C), 0.23 (9H, s, 3 × CH_3); **^{13}C NMR** ($CDCl_3$, 126 MHz) δ 88.0 (1C, Q), 84.7 (1C, Q), 68.3 (1C, CH), 66.6 (1C, Q), -0.6 (3C, 3CH_3). ^{1}H and ^{13}C NMR spectroscopic data are in good agreement with the literature.[4]

4-(4-Trimethylsilyl-1,3-butadiyn-1-yl)benzyl alcohol, S6

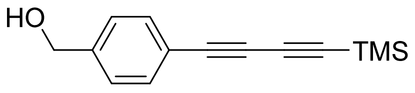

A mixture of 4-iodobenzyl alcohol **S4** (748 mg, 3.20 mmol), Et_3N (890 μL, 6.40 mmol), $PdCl_2(PPh_3)_2$ (45.5 mg, 65.0 μmol, 2 mol%) and CuI (34.2 mg, 180 μmol, 5.6 mol%) were dissolved in anhydrous Et_2O (4 mL). The reaction mixture was stirred for 10 minutes, after which 1-trimethylsilyl-1,3-butadiyne **S5** (20 mL, 3.2 mmol, 0.16 N in Et_2O) was added, and the mixture stirred at rt for 18 h. The resulting mixture was concentrated *in vacuo* and re-dissolved in CH_2Cl_2 (50 mL). The organic mixture was washed with HCl (2 × 40 mL, 1 N aq.) and water (40 mL). The combined aqueous washings were extracted CH_2Cl_2 (3 × 100 mL). The combined organic extracts were dried ($MgSO_4$), filtered and concentrated *in vacuo*. The crude material was purified by flash column chromatography (CH_2Cl_2) to afford the product as a light brown solid (272 mg, 37%). $\mathbf{R}_f$(CH_2Cl_2) = 0.36; **mp** 88 - 90 °C; **IR** (neat, cm^{-1}) 3256 (OH), 2203 (C≡C), 2102 (C≡C), 1508 (C=C); **^{1}H NMR** ($CDCl_3$, 500 MHz) δ 7.51 (2H, d, *J* 8.4 Hz, 2 × Ar*H*), 7.35 (2H, d, *J* 8.4 Hz, 2 × Ar*H*), 4.73 (2H, d, *J* 5.9 Hz, CH_2OH), 1.72 (1H, t, *J* 5.9 Hz, CH_2O*H*), 0.26 (9H, s, 3 × CH_3); **^{13}C NMR** ($CDCl_3$, 126 MHz) δ 142.2 (1C, Q), 132.9 (2C, CH), 126.8 (2C, CH), 120.6 (1C, Q), 90.8

(1C, Q), 87.8 (1C, Q), 76.6 (1C, Q), 74.2 (1C, Q), 64.8 (1C, CH_2), -0.4 (3C, CH_3); ***m/z*** (EI) 228.1 ($[M]^+$, 39%), 213.1 (100); **HRMS** (EI) calcd. for $C_{14}H_{16}OSi$ $[M]^+$ 228.0965, found 228.0978. ^{1}H NMR spectroscopic data is in good agreement with the literature.[5]

4-(4-Trimethylsilyl-1,3-butadiyn-1-yl)benzyl bromide, 4g-TMS

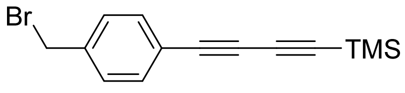

A solution of 4-(4-trimethylsilyl-1,3-butadiyn-1-yl)benzyl alcohol **S6** (91 mg, 400 µmol) in anhydrous Et_2O (1 mL) at 0 °C, was treated with pyridine (2.5 µL, 32 µmol) then PBr_3 (15.2 µL, 160 µmol). The reaction mixture was warmed to rt and stirred for 18 h in darkness. The reaction mixture was diluted with Et_2O (30 mL) and washed with Na_2CO_3 (15 mL; sat. aq.). The aqueous layer was separated and extracted with Et_2O (3 × 30 mL). The combined organic extracts were dried ($MgSO_4$), filtered and concentrated *in vacuo.* The crude material was purified by flash column chromatography (Hexane) to afford the product as a pale yellow solid (90 mg, 77%). $\mathbf{R}_f$ (Hexane) = 0.48; **mp** 65 – 67 °C; **IR** (neat, cm^{-1}) 2203 (C≡C), 2099 (C≡C); **^{1}H NMR** (CD_3OD, 500 MHz) δ 7.50 (2H, d, *J* 8.4 Hz, 2 × Ar*H*), 7.44 (2H, d, *J* 8.4 Hz, 2 × Ar*H*), 4.59 (2H, s, C*H*$_2$Br), 0.24 (9H, s, 3 × C*H*$_3$); **^{13}C NMR** (CD_3OD, 126 MHz) δ 140.0 (1C, Q), 132.6 (2C, CH), 129.1 (2C, CH), 121.0 (1C, Q), 90.0 (1C, Q), 87.2 (1C, Q), 75.6 (1C, Q), 74.0 (1C, Q), 31.7 (1C, CH_2), -1.9 (3C, CH_3); ***m/z*** (EI) 292.0 ($[^{81}BrM]^+$, 14%), 290.0 ($[^{79}BrM]^+$, 14), 211.1 (100), 196.0 (27), 183.0 (24); **HRMS** (EI) calcd. for $C_{14}H_{15}BrSi$ $[^{79}BrM]^+$ 290.0121, found 290.0132.

4-Phenyl-1-trimethylsilyl-1,3-butadiyne, S7

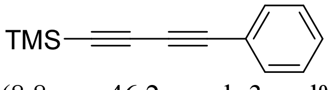

A mixture of iodobenzene (180 µL, 1.6 mmol), Et_3N (450 µL, 3.2 mmol), $PdCl_2(PPh_3)_2$ (11.5 mg, 16.4 µmol, 1 mol%) and CuI (8.8 mg, 46.2 µmol, 3 mol%) were dissolved in anhydrous Et_2O (2 mL). The mixture was stirred for 10 min, after which 1-trimethylsilyl-1,3-butadiyne **S5** (10 mL, 1.6 mmol, 0.16 N in Et_2O) was added, and the mixture stirred at rt for 18 h. The mixture was concentrated *in vacuo* and re-dissolved in CH_2Cl_2 (25 mL). The organic mixture was washed with HCl (2 × 20 mL, 1 N aq.) and water (20 mL). The combined aqueous washings were extracted CH_2Cl_2 (3 × 50 mL). The combined organic extracts were dried ($MgSO_4$), filtered and concentrated *in vacuo*. The crude material was purified by flash column chromatography (CH_2Cl_2) to afford the product as a pale yellow oil (260 mg, 82%). $\mathbf{R}_f$(Hexane) = 0.64; **IR** (neat, cm^{-1}) 2207 (C≡C), 2102 (C≡C); **^{1}H NMR** (CD_3OD, 500 MHz) δ 7.53 - 7.49 (2H, m, 2 × Ar*H*), 7.46 – 7.41 (1H, m, Ar*H*), 7.38 (2H, t, *J* 7.5 Hz, 2 × Ar*H*), 0.24 (9H, s, 3 × C*H*$_3$); **^{13}C NMR** (CD_3OD, 126 MHz) δ 132.2 (2C, CH), 129.3 (1C, CH), 128.3 (2C, CH), 121.1 (1C, Q), 89.5 (1C, Q), 87.4 (1C, Q), 76.0 (1C, Q), 73.3 (1C, Q), -1.8 (3C, CH_3); ***m/z*** (EI) 198.1 ($[M]^+$, 32%), 183.2 (100); **HRMS** (EI) *m/z* calcd. for $C_{13}H_{14}Si$ $[M]^+$ 198.0859, found 198.0862. ^{1}H and ^{13}C NMR spectroscopic data are in good agreement with the literature.[6]

5-Phenyl-2,4-pentadiyn-1-ol, S8

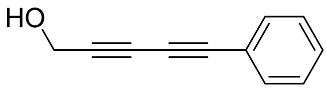

A solution of 4-phenyl-1-trimethylsilyl-1,3-butadiyne **S7** (198.3 mg, 1.00 mmol) in anhydrous THF (10 mL) was cooled to -10 °C, and treated with MeLi•LiBr complex (1.3 mL, 2.00 mmol, 1.5 N in Et_2O).

The mixture was stirred at -10 °C for 15 min, warmed to rt and stirred for an additional 2 h. After complete desilylation, the mixture was cooled to -10 °C, and a suspension of paraformaldehyde (90.7 mg, 3.02 mmol) in anhydrous THF (3.0 mL) was added. The mixture was warmed to rt and stirred for 18 h. The reaction was quenched by the slow addition of NH_4Cl (20 mL; sat. aq.), and the organic layer was washed with $NaHCO_3$ (20 mL; sat. aq.) and brine (20 mL). The combined aqueous layers were extracted with Et_2O (3 × 30 mL), and the combined organic extracts were dried ($MgSO_4$), filtered and concentrated *in vacuo*. The crude material was purified by flash column chromatography (Hexane:EtOAc, 10:1) to afford the product as a pale yellow oil (117.2 mg, 75%). **R_f** (Hexane:EtOAc, 5:2) = 0.54; **IR** (neat, cm^{-1}) 3293 (OH), 2241 (C≡C); **^{1}H NMR** (CD_3OD, 500 MHz) δ 7.53 – 7.48 (2H, m, 2 × Ar*H*), 7.45 – 7.35 (3H, m, 3 × Ar*H*), 4.34 (2H, s, C*H*$_2$OH); **^{13}C NMR** (CD_3OD, 126 MHz) δ 132.1 (2C, CH), 129.1 (1C, CH), 128.3 (2C, CH), 121.4 (1C, Q), 81.2 (1C, Q), 77.1 (1C, Q), 72.7 (1C, Q), 68.3 (1C, Q), 49.8 (1C, CH_2); ***m/z*** (EI) 156.0 ($[M]^+$, 100%), 139.0 (22), 128.1 (65), 102.0 (64); **HRMS** (EI) *m/z* calcd. for $C_{11}H_8O$ $[M]^+$ 156.0570, found 156.0565. ^{1}H and ^{13}C NMR spectroscopic data are in good agreement with the literature.[7]

5-Bromo-1-phenyl-1,3-pentadiyne, 4h

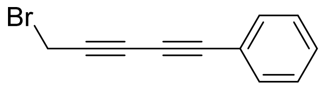

A solution of 5-phenyl-2,4-pentadiyn-1-ol **S8** (117.2 mg, 750 μmol) in anhydrous Et_2O (3 mL) at 0 °C, was treated with pyridine (4.8 μL, 60 μmol) then PBr_3 (28.5 μL, 300 μmol). The reaction mixture was warmed to rt and stirred for 18 h in darkness. The mixture was diluted with Et_2O (30 mL) and washed with Na_2CO_3 (15 mL; sat. aq.). The aqueous layer was separated and extracted with Et_2O (3 × 30 mL). The combined organic extracts were dried ($MgSO_4$), filtered and concentrated *in vacuo.* The crude material was purified by flash column chromatography (Hexane) to afford the product as a yellow oil (97 mg, 59 %). **R_f** (Hexane) = 0.47; **IR** (neat, cm^{-1}) 2243 (C≡C), 2222 (C≡C); **^{1}H NMR** (CD_3OD, 500 MHz) δ 7.55 – 7.51 (2H, m, 2 × Ar*H*), 7.47 – 7.42 (1H, m, Ar*H*), 7.41 – 7.37 (2H, m, 2 × Ar*H*), 4.24 (2H, s, C*H*$_2$Br); **^{13}C NMR** (CD_3OD, 126 MHz) δ 132.2 (2C, CH), 129.4 (1C, CH), 128.3 (2C, CH), 121.0 (1C, Q), 78.9 (1C, Q), 77.3 (1C, Q), 72.3 (1C, Q), 69.8 (1C, Q), 13.4 (1C, CH_2); ***m/z*** (EI) 219.9 ($[^{81}BrM]^+$, 11%), 217.9 ($[^{79}BrM]^+$, 11), 139.0 (100); **HRMS** (EI) calcd. for $C_{11}H_7Br$ $[^{79}BrM]^+$ 217.9726, found 217.9711.

4-(4-Phenyl-1,3-butadiyn-1-yl)benzyl alcohol, S9

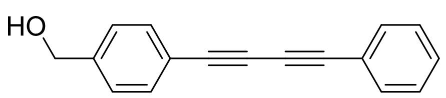

A solution of 4-phenyl-1-trimethylsilyl-1,3-butadiyne **S7** (100.1 mg, 500 μmol) in THF:MeOH (2 mL, 1:1 v/v) was treated with K_2CO_3 (138.2 mg, 1.00 mmol) and the mixture stirred at rt for 2 h. The reaction was quenched by the addition of NH_4Cl (2.0 mL, sat. aq.) and Et_2O (4.0 mL). The organic phase was separated, washed with brine (2 × 5 mL), dried ($MgSO_4$) and filtered. To the deprotected alkyne was added Et_3N (4.2 mL, 30.0 mmol), 4-iodobenzyl alcohol **S4** (117.1 mg, 500 μmol), $PdCl_2(PPh_3)_2$ (3.5 mg, 5.0 μmol, 1 mol%) and CuI (2.7 mg, 14 μmol, 2.8 mol%), and the mixture stirred at rt for 18 h. The reaction was quenched by the addition of NH_4Cl (10 mL, sat. aq.) and the resulting mixture extracted with Et_2O (3 × 20 mL). The combined organic extracts were washed with brine (15 mL), dried ($MgSO_4$), filtered and concentrated *in vacuo*. The crude material was purified by flash column chromatography (Hexane:EtOAc, 5:2) to give the product as a white solid (97.6 mg, 84%). **R_f** (Hexane:EtOAc, 5:2) = 0.35; **mp** 126 – 128 °C;

IR (neat, cm^{-1}) 3246 (OH), 2216 (C≡C), 2145 (C≡C); **^{1}H NMR** (CD_3OD, 500 MHz) δ 7.58 – 7.51 (4H, m, 4 × Ar*H*), 7.48 – 7.35 (5H, m, 5 × Ar*H*), 4.65 (2H, s, C*H*$_2$OH); **^{13}C NMR** (CD_3OD, 126 MHz) δ 143.3 (1C, Q), 132.1 (2C, CH), 132.0 (2C, CH), 129.1 (1C, CH), 128.3 (2C, CH), 126.6 (2C, CH), 121.6 (1C, Q), 120.2 (1C, Q), 80.9 (1C, Q), 80.7 (1C, Q), 73.0 (1C, Q), 72.8 (1C, Q), 63.2 (1C, CH_2); ***m/z*** (EI) 232.1 ($[M]^+$, 100%), 202.1 (44); **HRMS** (EI) calcd. for $C_{17}H_{12}O$ $[M]^+$ 232.0883, found 232.0889. ^{1}H and ^{13}C NMR spectroscopic data are in good agreement with the literature.[8]

4-(4-Phenyl-1,3-butadiyn-1-yl)benzyl bromide, 4i

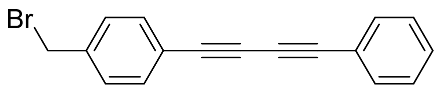

A solution of 4-(4-phenyl-1,3-butadiyn-1-yl)benzyl alcohol **S9** (116.1 mg, 500 µmol) in anhydrous Et_2O (3 mL) at 0 °C, was treated with pyridine (4.8 µL, 60 µmol) then PBr_3 (28.5 µL, 300 µmol). The reaction mixture was warmed to rt and stirred for 18 h in darkness. The reaction mixture was diluted with Et_2O (30 mL) and washed with Na_2CO_3 (15 mL; sat. aq.). The aqueous layer was separated and extracted with Et_2O (3 × 30 mL). The combined organic extracts were dried ($MgSO_4$), filtered and concentrated *in vacuo.* The crude material was purified by flash column chromatography (Hexane) to afford the product as white solid (91 mg, 62 %). **R*$_f$*** (Hexane) = 0.25; **IR** (neat, cm^{-1}) 2216 (C≡C), 2145 (C≡C); **^{1}H NMR** (CD_3OD, 500 MHz) δ 7.57 – 7.51 (4H, m, 4 × Ar*H*), 7.49 – 7.38 (5H, m, 5 × Ar*H*), 4.60 (2H, s, C*H*$_2$Br); **^{13}C NMR** (CD_3OD, 126 MHz) δ 139.8 (1C, Q), 132.4 (2C, CH), 132.1 (2C, CH), 129.2 (1C, CH), 129.1 (2C, CH), 128.3 (2C, CH), 121.4 (1C, Q), 121.4 (1C, Q), 81.2 (1C, Q), 80.4 (1C, Q), 73.7 (1C, Q), 72.9 (1C, Q), 31.7 (1C, CH_2); ***m/z*** (EI) 295.9 ($[^{81}BrM]^+$, 12%), 293.9 ($[^{79}BrM]^+$, 12), 215.0 (100), 213.0 (44), 107.4 (10); **HRMS** (EI) calcd. for $C_{17}H_{11}Br$ $[^{79}BrM]^+$ 294.0039, found 294.0034.

Characterisation of anisomycin analogues 2b-i

General procedure for the synthesis of Raman-labelled anisomycin derivatives

To a solution of **ANS** (60.0 µmol) in DMF (1.5 mL) was added potassium carbonate (60.0 µmol) and **4b-i** (60.0 µmol), the solution was stirred at room temperature for 9 h. The solution was then concentrated *in vacuo*, and the residue was purified by RP HPLC, and freeze dried to afford the product.

***N*-Cyanomethyl anisomycin, 2b**

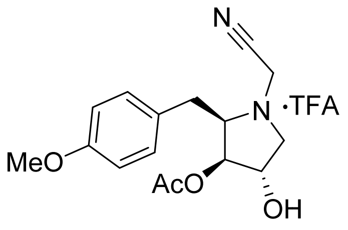

Purification (Method A); colourless oil (20.9 mg, 87%). **R$_t$** (Method B) = 19.4 min; **IR** (neat, cm^{-1}) 3410 (OH), 2363 (C≡N), 1748 (C=O), 1672 (C=O, TFA), 1613 (C=C), 1585 (C=C), 1514 (C=C); **^{1}H NMR** ($CDCl_3$, 500 MHz) δ 7.17 (2H, d, *J* 8.6 Hz, 2 × Ar*H*), 6.89 (2H, d, *J* 8.6 Hz, 2 × Ar*H*), 4.82 (1H, d, *J* 3.7 Hz, C_3H), 4.31 – 4.28 (1H, m C_4H), 3.89 – 3.70 (2H, dd, *J* 41.5, 17.5 Hz, NC*H*$_2$C≡N), 3.82 (3H, s, O*Me*), 3.79 (1H, dd, *J* 11.2, 6.1 Hz, $C_5H_aH_b$), 3.65 (1H, dd, *J* 12.4, 7.4 Hz, C_2H), 3.01 – 2.92 (2H, m, C*H*$_2$Ar), 2.86 (1H, dd, *J* 11.2, 6.1 Hz, $C_5H_aH_b$), 2.19 (3H, s, O*Ac*); **^{13}C NMR** ($CDCl_3$, 126 MHz) δ 170.9 (1C, Q), 158.8 (1C, Q), 129.8 (2C, CH), 127.9 (1C, Q), 114.5 (2C, CH), 112.8 (1C, Q), 80.1 (1C, CH), 73.5 (1C, CH), 65.8 (1C, CH), 59.1 (1C, CH_2), 55.3

(1C, CH_3), 40.6 (1C, CH_2), 31.4 (1C, CH_2), 20.7 (1C, CH_3); ***m/z*** (ESI) 305.2 ($[M + H]^+$, 100%), 263.1 (11), 179.0 (41), 117.0 (22); **HRMS** (ESI) calcd. for $C_{16}H_{20}O_4N_2Na$ $[M + Na]^+$ 327.1315, found 327.1315.

N-Propargyl anisomycin, 2c

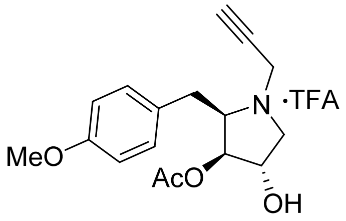

Purification (Method A); colourless oil (21.6 mg, 90%). $\mathbf{R_t}$ (Method B) = 16.6 min; **IR** (neat, cm^{-1}) 3277 (OH), 2131 (C≡C), 1749 (C=O), 1672 (C=O, TFA), 1613 (C=C), 1585 (C=C), 1514 (C=C); **^{1}H NMR** ($CDCl_3$, 500 MHz) δ 7.14 (1H, d, *J* 8.6 Hz, 2 × ArH), 6.88 (1H, d, *J* 8.6 Hz, 2 × ArH), 4.82 (1H, d, *J* 4.0 Hz, C_3H), 4.40 – 4.36 (1H, m, C_4H), 4.22 – 4.15 (1H, m, $C_5H_aH_b$), 4.03 – 4.00 (1H, m, C_2H), 3.95 (2H, ddd, *J* 38.2, 17.2, 2.1 Hz, $NCH_2C{\equiv}CH$), 3.82 (3H, s, O*Me*), 3.21 – 3.15 (1H, m, $C_5H_aH_b$), 3.15 – 3.07 (1H, m, CH_2Ar), 2.62 (1H, t, *J* 2.1 Hz, $NCH_2C{\equiv}CH$), 2.20 (3H, s, O*Ac*); **^{13}C NMR** ($CDCl_3$, 126 MHz) δ 170.4 (1C, Q), 158.9 (1C, Q), 129.8 (2C, CH), 127.2 (1C, Q), 114.5 (2C, CH), 78.74 (1C, Q), 78.68 (1C, CH), 77.2 (1C, CH),* 72.3 (1C, CH), 63.3 (1C, CH), 57.8 (1C, CH_2), 55.3 (1C, CH_3), 41.2 (1C, CH_2), 29.6 (1C, CH_2), 20.6 (1C, CH_3); ***m/z*** (ESI) 326.1 ($[M + Na]^+$, 100%), 304.2 ($[M + H]^+$, 8), 284.1 (18), 262.1 (15), 244.1 (6); **HRMS** (ESI) calcd. for $C_{17}H_{22}O_4N$ $[M + H]^+$ 304.1549, found 304.1547. *Obscured by solvent peak – observed in HSQC.

N-2-Butyn-1-yl anisomycin, 2d

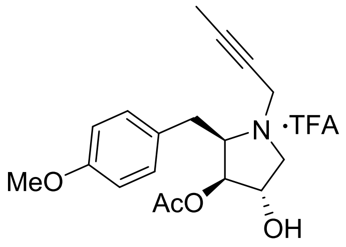

Purification (Method A); colourless oil (17.6 mg, 71%). $\mathbf{R_t}$ (Method B) = 23.1 min; **IR** (neat, cm^{-1}) 3271 (OH), 2247 (C≡C), 1751 (C=O), 1670 (C=O, TFA), 1612 (C=C), 1585 (C=C), 1514 (C=C); **^{1}H NMR** ($CDCl_3$, 500 MHz) δ 7.11 (2H, d, *J* 8.2 Hz, 2 × Ar*H*), 6.87 (2H, d, *J* 8.2 Hz, 2 × Ar*H*), 4.87 (1H, s, C_3H), 4.34 (1H, s, C_4H), 4.18 (1H, br. s, $C_5H_aH_b$), 4.08 (1H, br. s, C_2H), 3.91 (2H, dd, *J* 31.0, 17.0 Hz, $NCH_2C{\equiv}CMe$), 3.81 (3H, s, O*Me*), 3.24 (1H, d, *J* 11.6 Hz, $C_5H_aH_b$), 3.17 – 3.06 (2H, m, CH_2Ar), 2.17 (3H, s, O*Ac*), 1.94 (3H, s, $NCH_2C{\equiv}CMe$); **^{13}C NMR** ($CDCl_3$, 126 MHz) δ 170.2 (1C, Q), 158.9 (1C, Q), 129.8 (2C, CH), 127.1 (1C, Q), 114.5 (2C, CH), 87.8 (1C, Q), 78.0 (1C, CH), 71.7 (1C, CH), 66.7 (1C, Q), 65.7 (1C, CH), 58.0 (1C, CH_2), 55.3 (1C, CH_3), 42.5 (1C, CH_2), 29.4 (1C, CH_2), 20.6 (1C, CH_3), 3.6 (1C, CH_3); ***m/z*** (ESI) 318.2 ($[M + H]^+$, 100%); **HRMS** (ESI) calcd. for $C_{18}H_{24}O_4N$ $[M + H]^+$ 318.1700, found 318.1705.

N-2,4-Pentadiyn-1-yl anisomycin, 2e

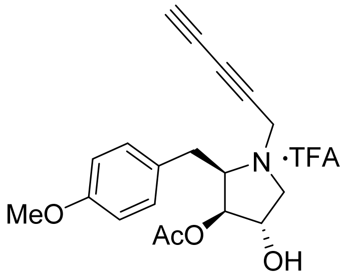

Purification (Method A); pale yellow oil (18.8 mg, 74%). $\mathbf{R_t}$ (Method B) = 24.2 min; **IR** (neat, cm^{-1}) 3271 (OH), 2236 (C≡C), 1751 (C=O), 1670 (C=O, TFA), 1613 (C=C), 1585 (C=C), 1514 (C=C); **^{1}H NMR** ($CDCl_3$, 500 MHz) δ 7.15 (2H, d, *J* 8.6 Hz, 2 × Ar*H*), 6.89 (2H, d, *J* 8.6 Hz, 2 × Ar*H*), 4.90 – 4.85 (1H, m, C_3H), 4.39 – 4.35 (1H, m, C_4H), 4.12 (1H, dd, *J* 12.1, 5.7 Hz, $C_5H_aH_b$), 4.00 – 3.94 (1H, m, C_2H), 3.97 (2H, dd, *J* 59.7, 18.0 Hz, $NCH_2C{\equiv}C$-), 3.83 (3H, s, O*Me*),

3.17 – 3.06 (3H, m, $C_5H_aH_b$ + CH_2Ar), 2.29 (1H, s, -C≡C*H*), 2.20 (3H, s, O*Ac*); **^{13}C NMR** ($CDCl_3$, 126 MHz) δ 170.2 (1C, Q), 158.9 (1C, Q), 129.8 (2C, CH), 127.1 (1C, Q), 114.6 (2C, CH), 78.3 (1C, CH), 74.1 (1C, Q), 72.0 (1C, CH), 69.6 (1C, Q), 66.5 (1C, Q), 66.3 (1C, CH), 64.9 (1C, Q), 58.6 (1C, CH_2), 55.3 (1C, CH_3), 42.6 (1C, CH_2), 29.9 (1C, CH_2), 20.6 (1C, CH_3); ***m/z*** (ESI) 328.2 ($[M + H]^+$, 100%), 179.0 (7); **HRMS** (EI) calcd. for $C_{19}H_{22}O_4N$ $[M + H]^+$ 328.1543, found 328.1544.

N-[6-$^2H_3$]-2,4-Hexadiyn-1-yl anisomycin, 2f

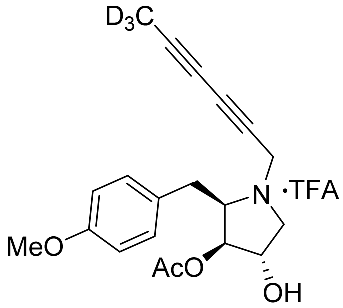

Purification (Method A); colourless oil (20.4 mg, 77%). **R_t** (Method C) = 21.1 min; **IR** (neat, cm^{-1}) 3289 (OH), 2264 (C≡C), 1751 (C=O), 1670 (C=O, TFA), 1613 (C=C), 1585 (C=C), 1514 (C=C); **^{1}H NMR** ($CDCl_3$, 500 MHz) δ 7.15 (2H, d, *J* 8.6 Hz, 2 × Ar*H*), 6.89 (2H, d, *J* 8.6 Hz, 2 × Ar*H*), 4.90 (1H, d, *J* 3.7 Hz, C_3H), 4.41 – 4.38 (1H, m, C_4H), 4.23 (1H, dd, *J* 12.5, 5.6 Hz, $C_5H_aH_b$), 4.12 – 4.07 (1H, m, C_2H) 4.04 (2H, dd, *J* 51.5, 17.8 Hz, NCH_2C≡C-), 3.83 (3H, s, O*Me*), 3.24 (1H, d, *J* 12.5 Hz, $C_5H_aH_b$), 3.19 – 3.09 (2H, m, CH_2Ar), 2.20 (3H, s, O*Ac*); **^{13}C NMR** ($CDCl_3$, 126 MHz) δ 170.2 (1C, Q), 159.0 (1C, Q), 129.8 (2C, CH), 126.8 (1C, Q), 114.6 (2C, CH), 79.1 (1C, Q), 78.1 (1C, CH), 75.8 (1C, Q), 71.9 (1C, CH), 66.0 (1C, CH), 63.0 (1C, Q), 62.0 (1C, Q), 58.2 (1C, CH_2), 55.3 (1C, CH_3), 42.6 (1C, CH_2), 29.5 (1C, CH_2), 20.5 (1C, CH_3) 3.73 (1C, m, CD_3); ***m/z*** (ESI) 345.2 ($[M + H]^+$, 100%), 179.0 (6); **HRMS** (ESI) calcd. for $C_{20}H_{21}D_3O_4N$ $[M + H]^+$ 345.1888, found 345.1888.

N-4-(1,3-Butadiyn-1-yl)benzyl anisomycin, 2g

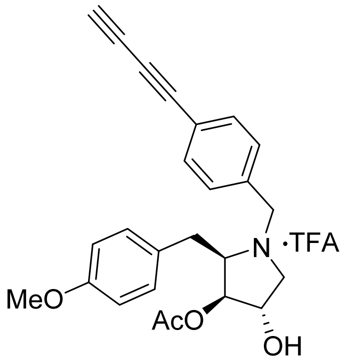

Purification (Method A); light brown oil (28.5 mg, 95%). **R_t** (Method B) = 28.2 min; **IR** (neat, cm^{-1}) 3273 (OH), 1751 (C=O), 1670 (C=O, TFA), 1613 (C=C), 1585 (C=C), 1514 (C=C); **^{1}H NMR** ($CDCl_3$, 500 MHz) δ 7.58 (2H, d, *J* 8.2 Hz, 2 × Ar*H*), 7.49 (2H, d, *J* 8.2 Hz, 2 × Ar*H*), 6.99 (2H, d, *J* 8.6 Hz, 2 × Ar*H*), 6.84 (2H, d, *J* 8.6 Hz, 2 × Ar*H*), 4.93 (1H, d, *J* 3.2 Hz, C_3H), 4.32 – 4.23 (3H, m, C_4H + NCH_2Ar), 4.03 – 3.89 (2H, m, C_2H + $C_5H_aH_b$), 3.79 (3H, s, O*Me*), 3.15 – 3.08 (2H, m, $C_5H_aH_b$ + CH_XH_YAr), 2.91 – 2.84 (1H, m, CH_XH_YAr), 2.55 (1H, s, -C≡C*H*), 2.16 (3H, s, O*Ac*); **^{13}C NMR** ($CDCl_3$, 126 MHz) δ 169.9 (1C, Q), 158.9 (1C, Q), 133.5 (2C, CH), 130.9 (2C, CH), 129.7 (2C, CH), 127.2 (1C, Q), 123.0 (1C, Q), 117.9 (1C, Q), 114.5 (2C, CH), 77.8 (1C, Q), 77.2 (1C, CH),* 75.3 (1C, Q), 74.0 (1C, Q), 72.3 (1C, CH), 72.3 (1C, CH), 67.8 (1C, CH), 59.9 (1C, CH_2), 58.8 (1C, CH_2), 55.3 (1C, CH_3), 30.4 (1C, CH_2), 20.6 (1C, CH_3); ***m/z*** (ESI) 404.2 ($[M + H]^+$, 100%), 179.0 (17); **HRMS** (ESI) calcd. for $C_{25}H_{26}O_4N$ $[M + H]^+$ 404.1856, found 404.1869. * Obscured by solvent peak – observed in HSQC.

***N*-5-Phenyl-2,4-pentadiyn-1-yl anisomycin, 2h (PhDY-ANS)**

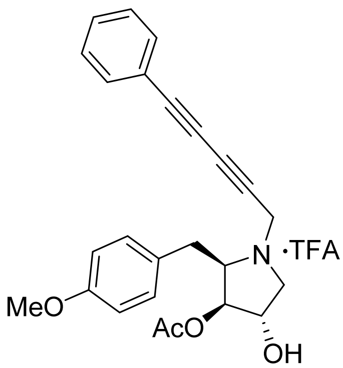

Purification (isocratic 60:40 H_2O: MeCN 0.1% TFA), light brown oil (21.0 mg, 73%). $\mathbf{R_t}$ (Method C) = 31.3 min; **IR** (neat, cm^{-1}) 3284 (OH), 2253 (C≡C), 1751 (C=O), 1672 (C=O, TFA), 1612 (C=C), 1585 (C=C), 1514 (C=C); **^{1}H NMR** ($CDCl_3$, 500 MHz) δ 7.58 – 7.55 (2H, m, 2 × Ar*H*), 7.47 – 7.43 (1H, m, Ar*H*), 7.41 – 7.34 (2H, m, 2 × Ar*H*), 7.18 (2H, d, *J* 8.6 Hz, 2 × Ar*H*), 6.90 (2H, d, *J* 8.6 Hz, 2 × Ar*H*), 4.90 (1H, d, *J* 3.8 Hz, C_3H), 4.41 (1H, br s, C_4H), 4.23 – 4.18 (1H, m, $C_5H_aH_b$), 4.15 – 4.02 (3H, m, C_2H + NCH_2C≡C-), 3.83 (3H, s, O*Me*), 3.25 – 3.22 (1H, m, $C_5H_aH_b$), 3.19 – 3.10 (2H, m, CH_2Ar), 2.21 (3H, s, O*Ac*); **^{13}C NMR** ($CDCl_3$, 126 MHz) δ 170.3 (1C, Q), 159.0 (1C, Q), 132.9 (2C, CH), 130.1 (1C, CH), 129.8 (2C, CH), 128.6 (2C, CH), 127.1 (1C, Q), 120.5 (1C, Q), 114.6 (2C, CH), 79.5 (1C, Q), 78.6 (1C, CH),* 74.9 (1C, Q), 72.3 (1C, CH), 72.3 (1C, Q), 65.7 (1C, CH), 58.1 (1C, CH_2), 55.3 (1C, CH_3), 42.5 (1C, CH_2), 29.7 (1C, CH_2), 20.6 (1C, CH_3); ***m/z*** (ESI) 404.2 ($[M + H]^+$, 100%), 324.9 (4), 246.9 (5), 179.0 (9); **HRMS** (ESI) calcd. for $C_{25}H_{26}O_4N$ $[M + H]^+$ 404.1856, found 404.1861. *Q peak expected at 77.2 ppm obscured by solvent peak.

***N*-4-(4-Phenyl-1,3-butadiyn-1-yl)benzyl anisomycin, 2i (BADY-ANS)**

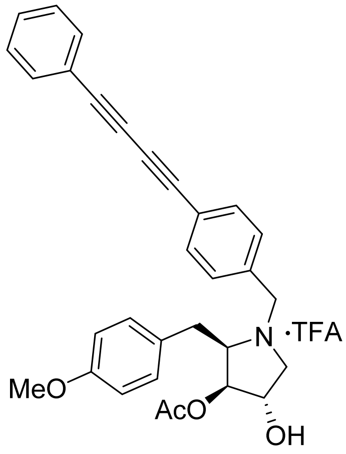

Purification (isocratic 50:50 H_2O:MeCN 0.1% TFA), pale yellow oil (28.0 mg, 81%). $\mathbf{R_t}$ (Method C) = 29.9 min; **IR** (neat, cm^{-1}) 3284 (OH), 2218 (C≡C), 1751 (C=O), 1670 (C=O, TFA), 1613 (C=C), 1585 (C=C), 1514 (C=C); **^{1}H NMR** ($CDCl_3$, 500 MHz) δ 7.61 – 7.54 (4H, m, 4 × Ar*H*), 7.50 (2H, d, *J* 7.9 Hz, 2 × Ar*H*), 7.43 – 7.34 (3H, m, 3 × Ar*H*), 6.99 (2H, d, *J* 8.6 Hz, 2 × Ar*H*), 6.84 (2H, d, *J* 8.6 Hz, 2 × Ar*H*), 4.95 (1H, s, C_3H), 4.32 – 4.25 (3H, m, C_4H + NCH_2Ar), 4.05 – 3.87 (2H, m, C_2H + $C_5H_aH_b$), 3.79 (3H, s, O*Me*), 3.17 – 3.06 (2H, m, CH_XH_YAr + $C_5H_aH_b$), 2.91 – 2.81 (1H, m, CH_XH_YAr), 2.15 (3H, s, O*Ac*); **^{13}C NMR** ($CDCl_3$, 126 MHz) δ 169.9 (1C, Q), 158.9 (1C, Q), 133.2 (2C, CH), 132.6 (2C, CH), 131.0 (2C, CH), 129.7 (2C, CH), 129.5 (1C, CH), 128.5 (2C, CH), 127.3 (1C, Q), 123.8 (1C, Q), 121.5 (2C, Q), 114.5 (2C, CH), 82.6 (1C, Q), 80.2 (1C, Q), 77.2 (1C, CH),* 75.0 (1C, Q), 73.6 (1C, Q), 72.3 (1C, CH), 69.2 (1C, CH), 60.2 (1C, CH_2), 58.9 (1C, CH_2), 55.3 (1C, CH_3), 30.5 (1C, CH_2), 20.6 (1C, CH_3); ***m/z*** (ESI) 480.2 ($[M + H]^+$, 100%), 324.9 (4), 246.9 (6); **HRMS** (ESI) calcd. for $C_{31}H_{30}O_4N$ $[M + H]^+$ 480.2169, found 480.2179. *Obscured by solvent peak – observed in HSQC.

References

1. N. P. Bowling, N. J. Burrmann, R. J. Halter, J. A. Hodges and R. J. McMahon, *J. Org. Chem.*, 2010, **75**, 6382-6390.
2. B. F. Coles and D. R. M. Walton, *Synthesis*, 1975, **6**, 390-391.
3. C.-R. Elie, N. Noujeim, C. Pardin and A. R. Schmitzer, *Chem. Commun.*, 2011, **47**, 1788-1790.
4. F. Ungeheuer and A. Fürstner, *Chem. − Eur. J.*, 2015, **21**, 11387-11392.
5. V. T. Tripp, J. S. Lampkowski, R. Tyler and D. D. Young, *ACS Comb. Sci.*, 2014, **16**, 164-167.
6. K. Semba, T. Fujihara, T. Xu, J. Terao and Y. Tsuji, *Adv. Synth. Catal.*, 2012, **354**, 1542-1550.
7. W. Yin, C. He, M. Chen, H. Zhang and A. Lei, *Org. Lett.*, 2009, **11**, 709-712.
8. H. Yamakoshi, A. Palonpon, K. Dodo, J. Ando, S. Kawata, K. Fujita and M. Sodeoka, *Bioorg. Med. Chem. Lett.*, 2015, **25**, 664-667.

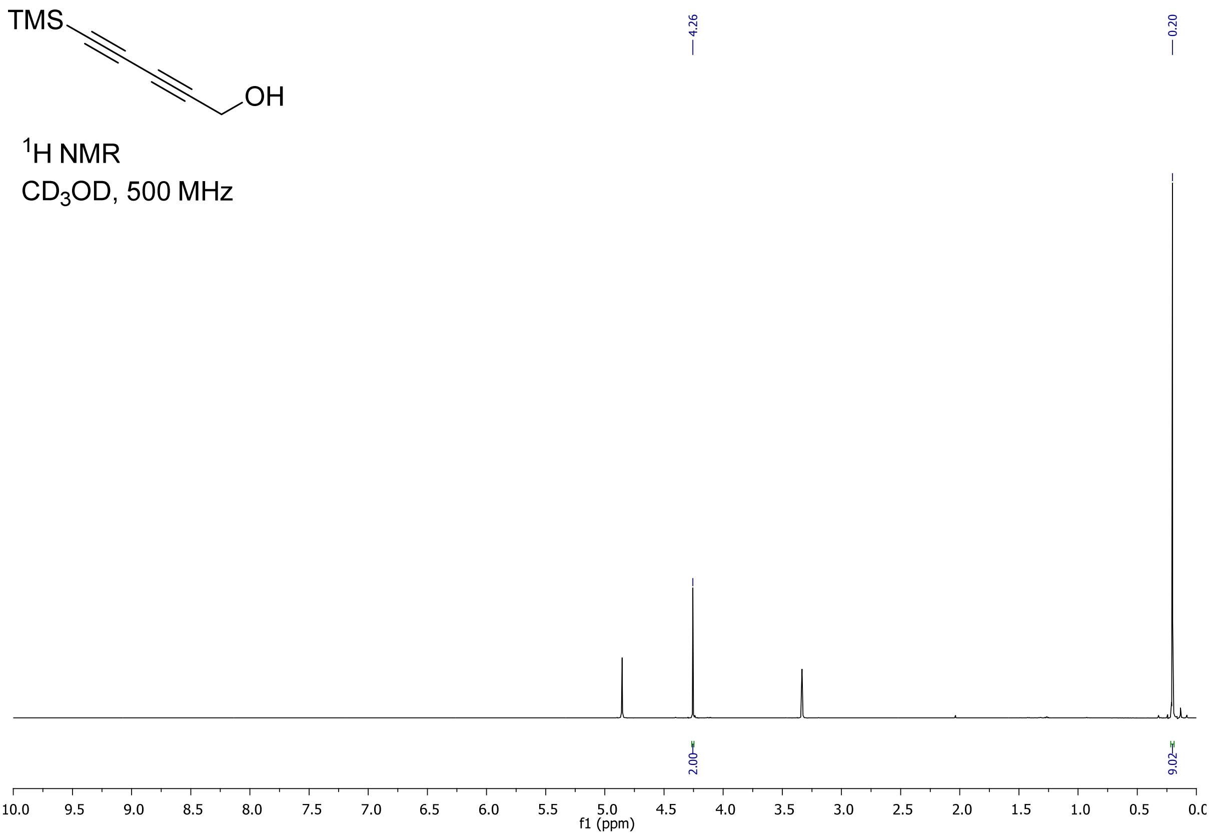
TMS
OH
1H NMR
CD3OD, 500 MHz
4.26
0.20
2.00
9.02
f1 (ppm)

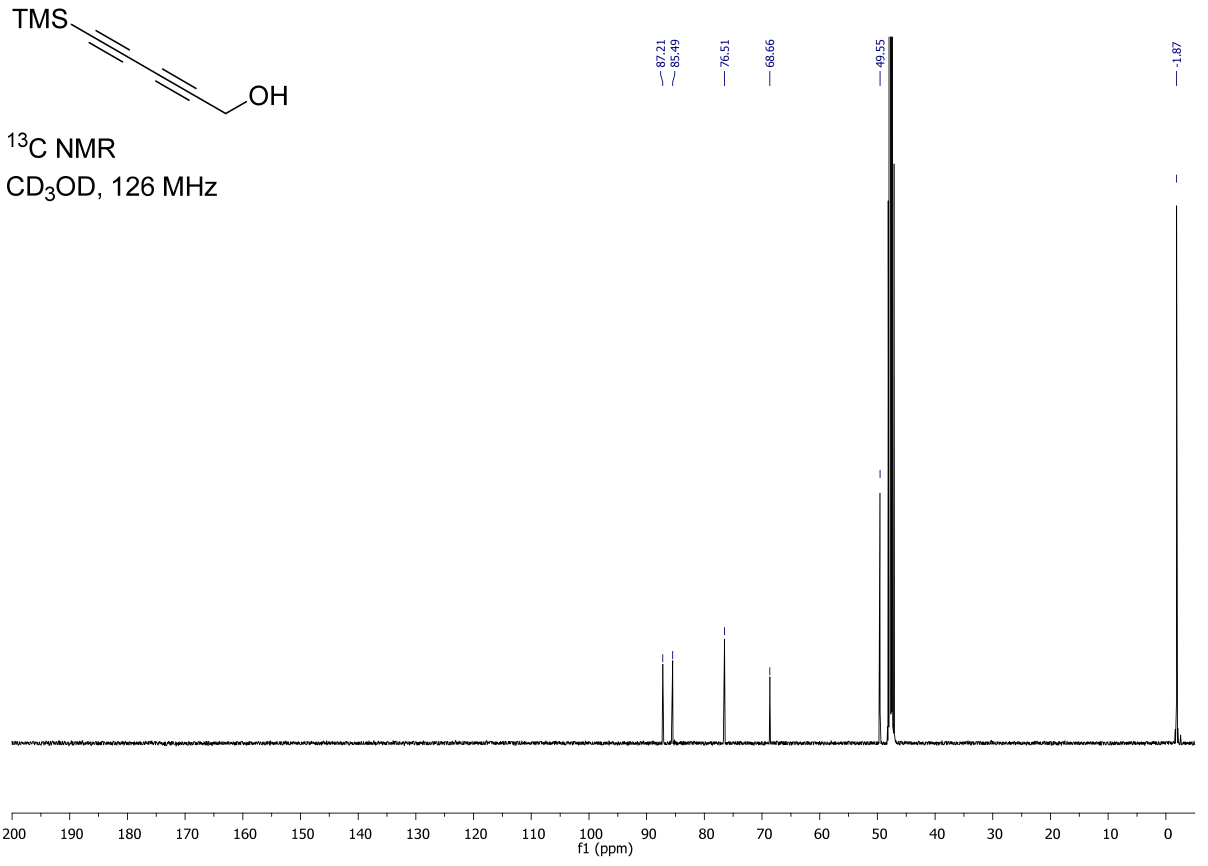
TMS
OH
13C NMR
CD3OD, 126 MHz
87.21
85.49
76.51
68.66
49.55
-1.87
f1 (ppm)

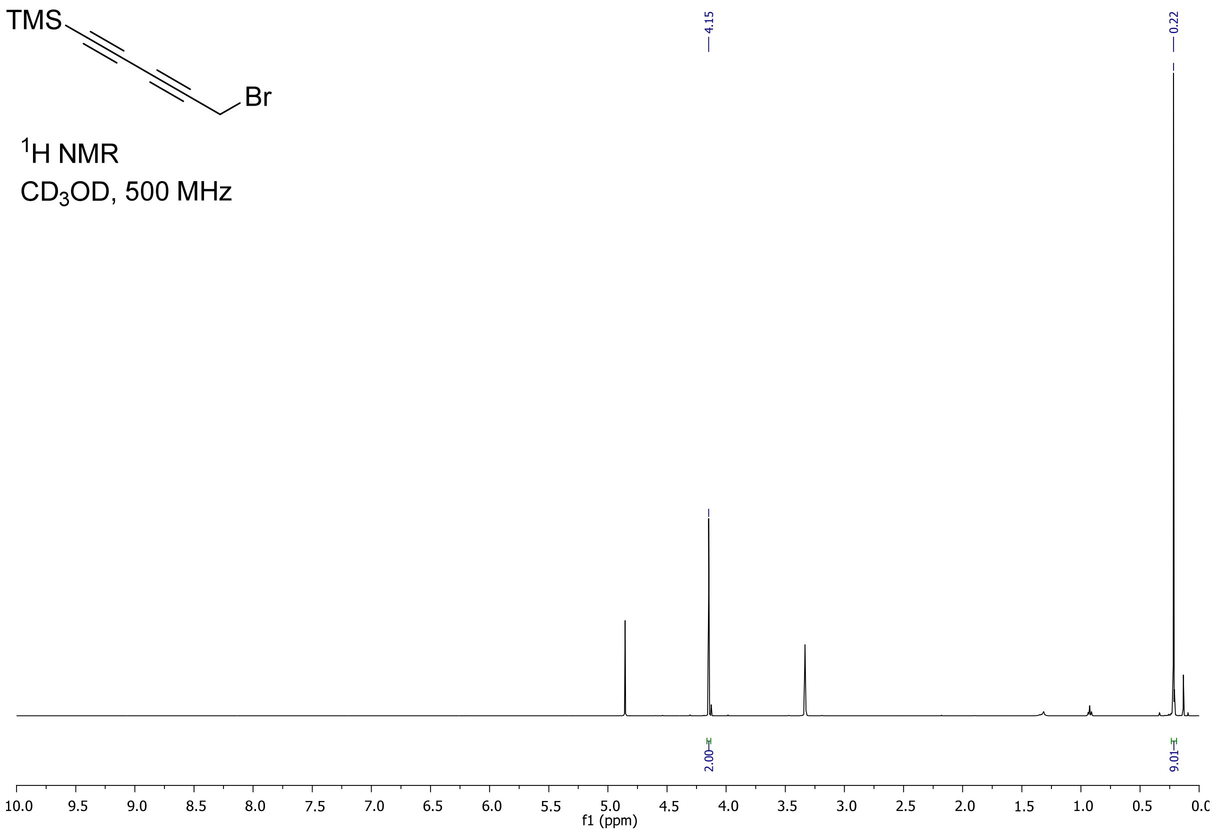
TMS
Br
^{1}H NMR
CD$_3$OD, 500 MHz
4.15
0.22
2.00
9.01
f1 (ppm)

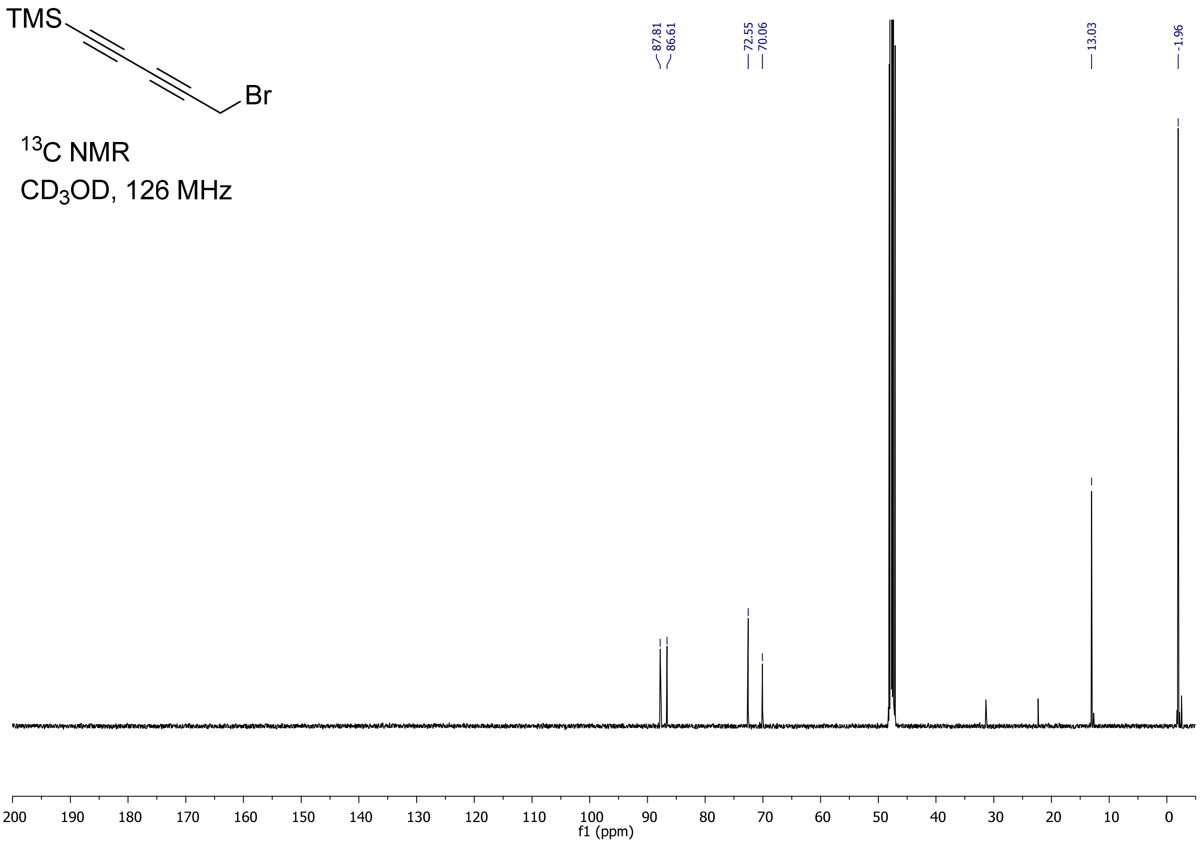
TMS
Br
^{13}C NMR
CD$_3$OD, 126 MHz
87.81
86.61
72.55
70.06
13.03
-1.96
f1 (ppm)

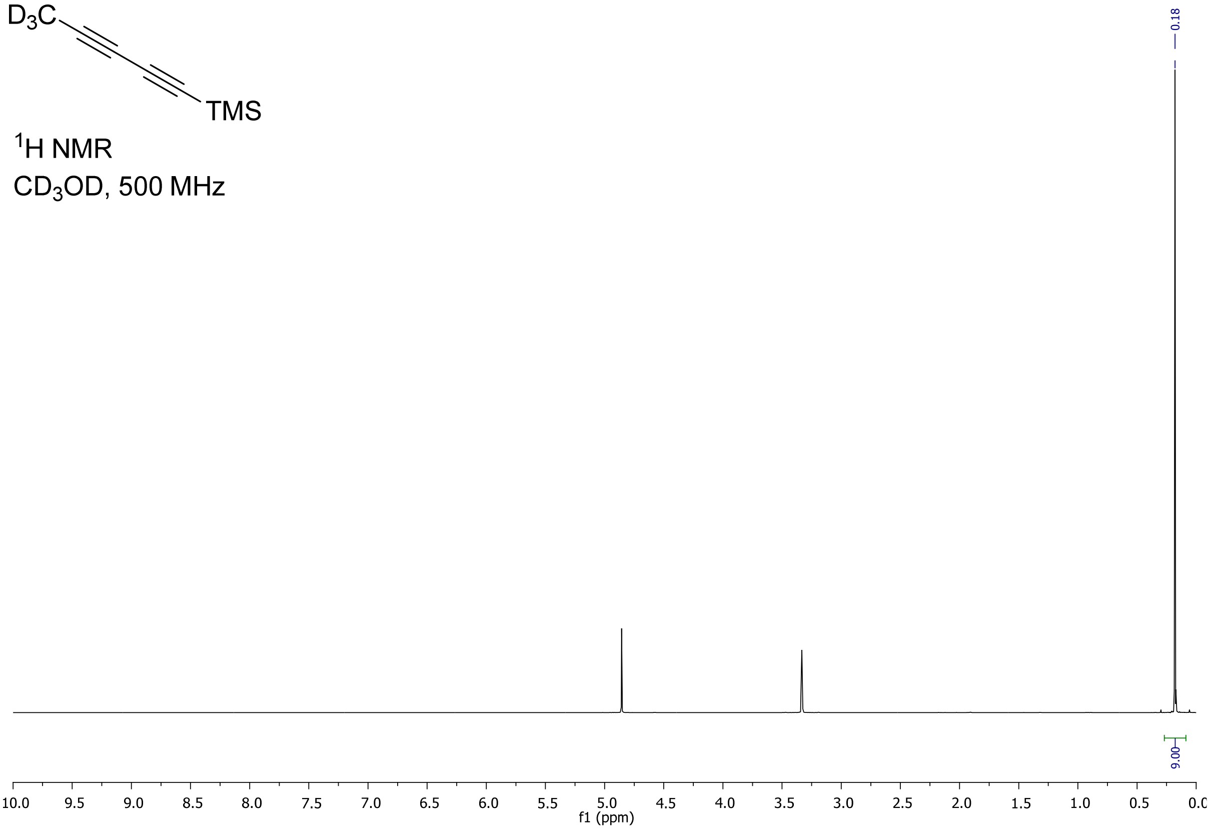
D3C
TMS
1H NMR
CD3OD, 500 MHz
0.18
9.00
f1 (ppm)

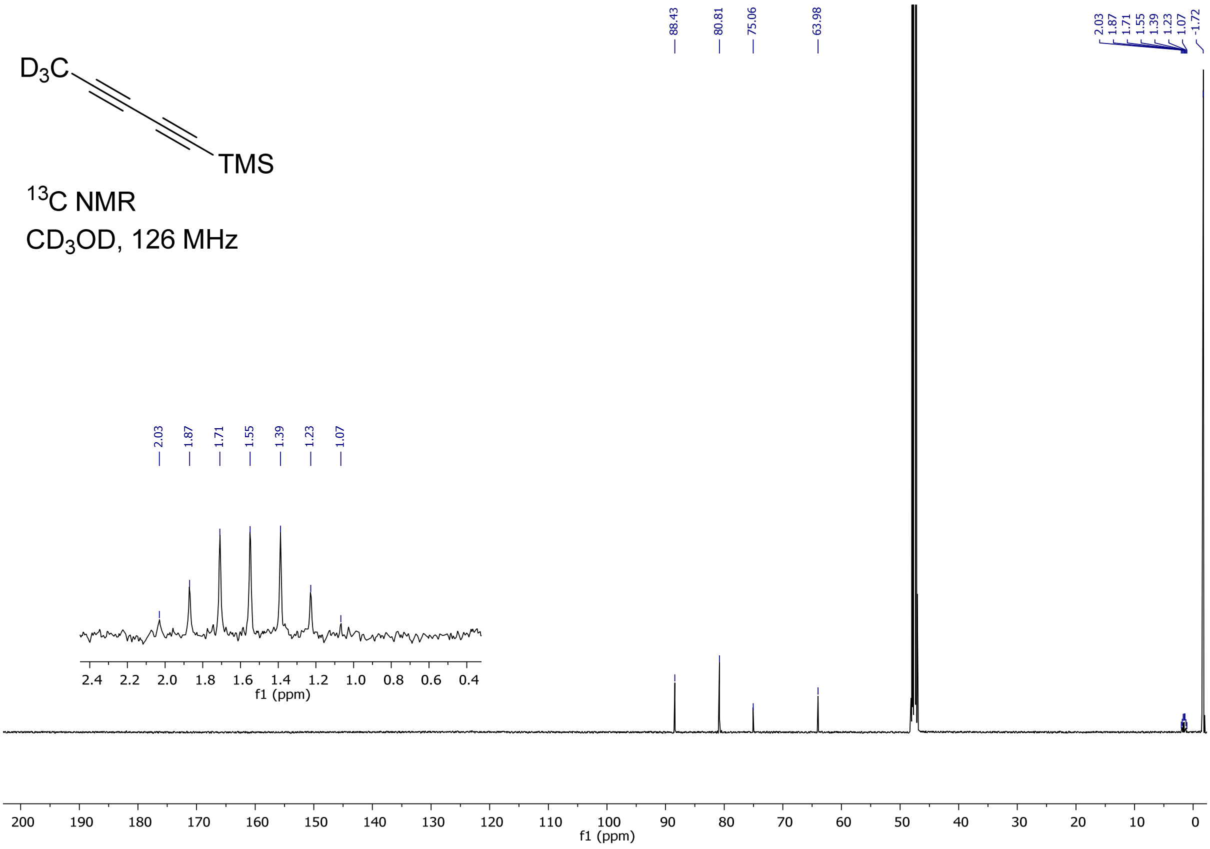
D3C
TMS
13C NMR
CD3OD, 126 MHz
88.43
80.81
75.06
63.98
2.03
1.87
1.71
1.55
1.39
1.23
1.07
-1.72
f1 (ppm)

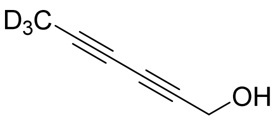

^{1}H NMR

CD_3OD, 500 MHz

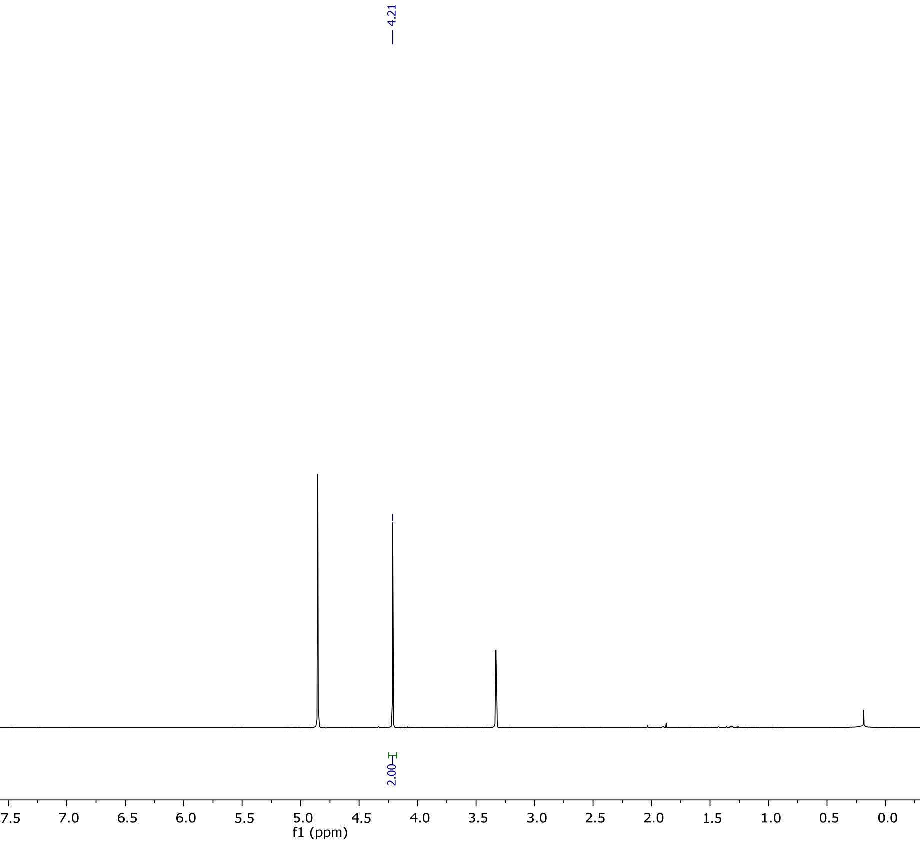

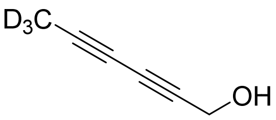

^{13}C NMR

CD_3OD, 126 MHz

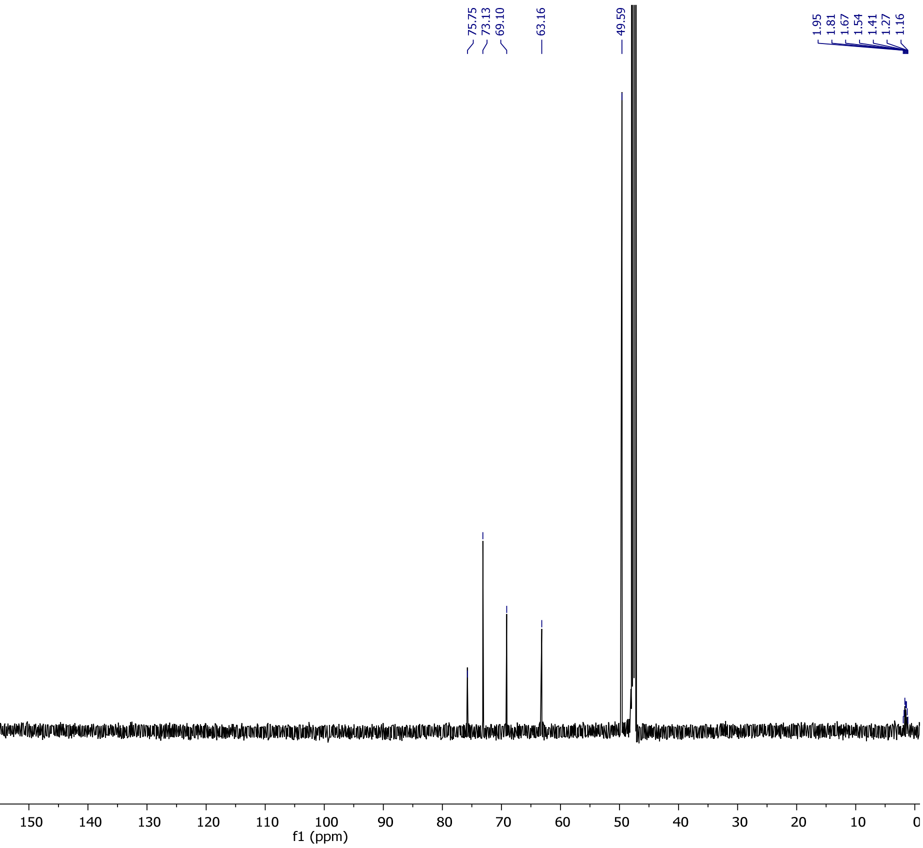

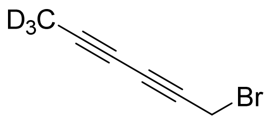

^{1}H NMR
CD_3OD, 600 MHz

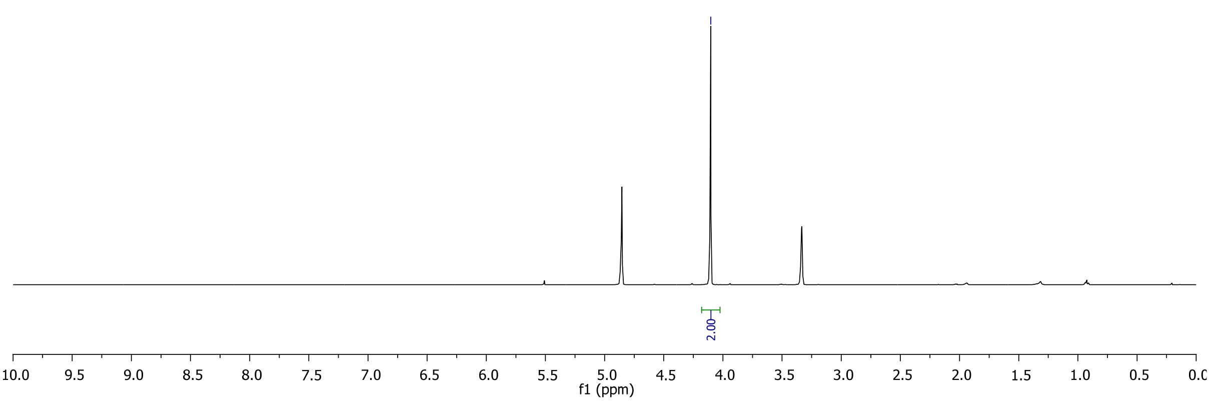

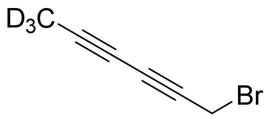

^{13}C NMR
CD_3OD, 151 MHz

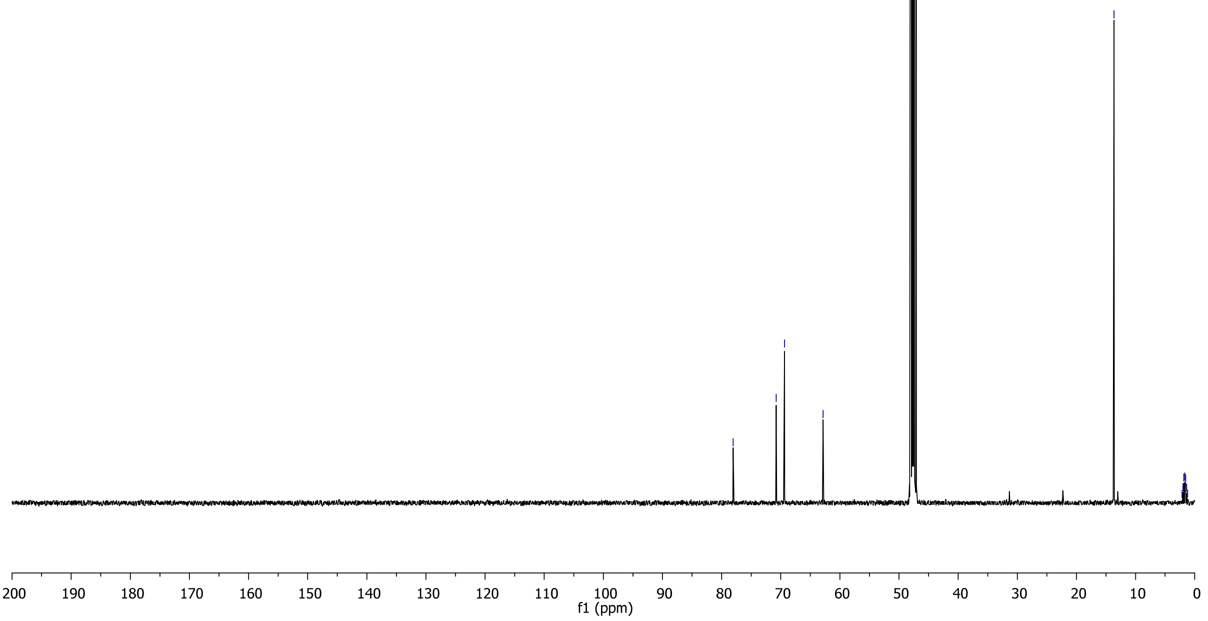

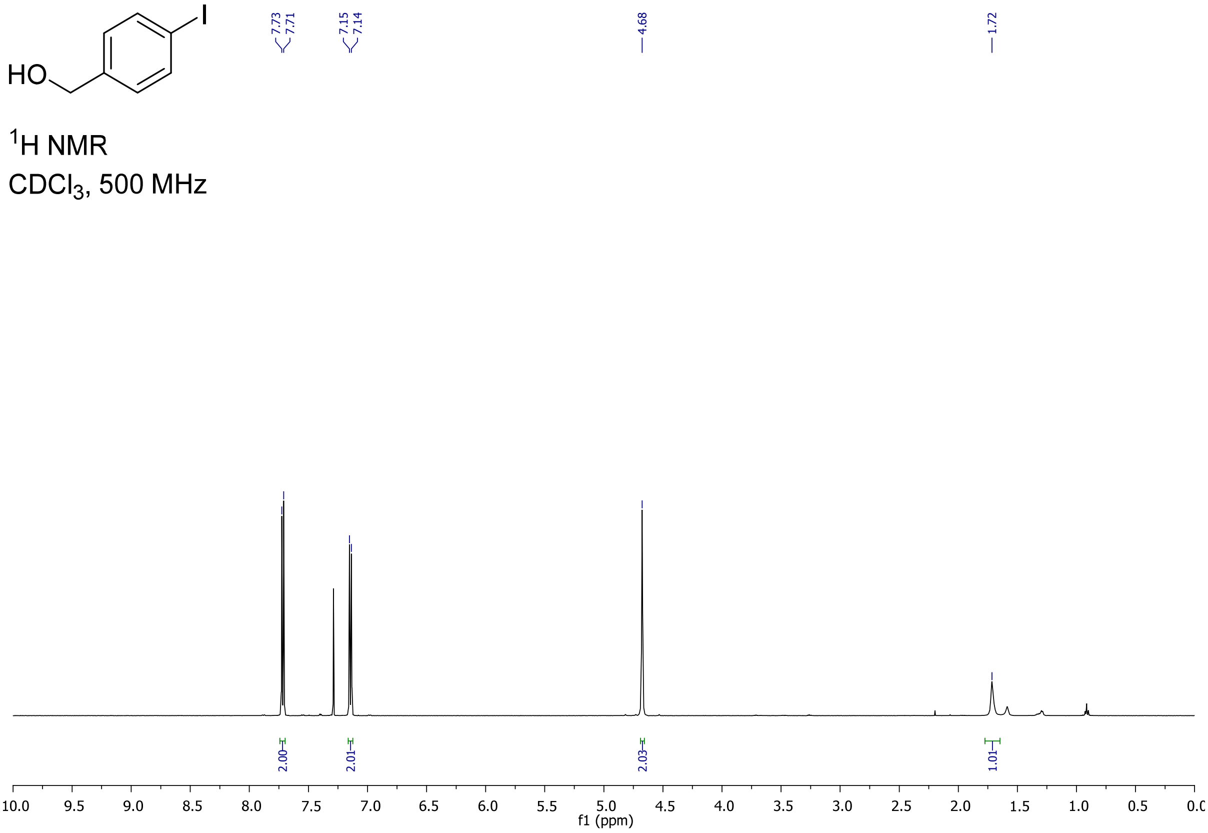

^{1}H NMR
$CDCl_3$, 500 MHz

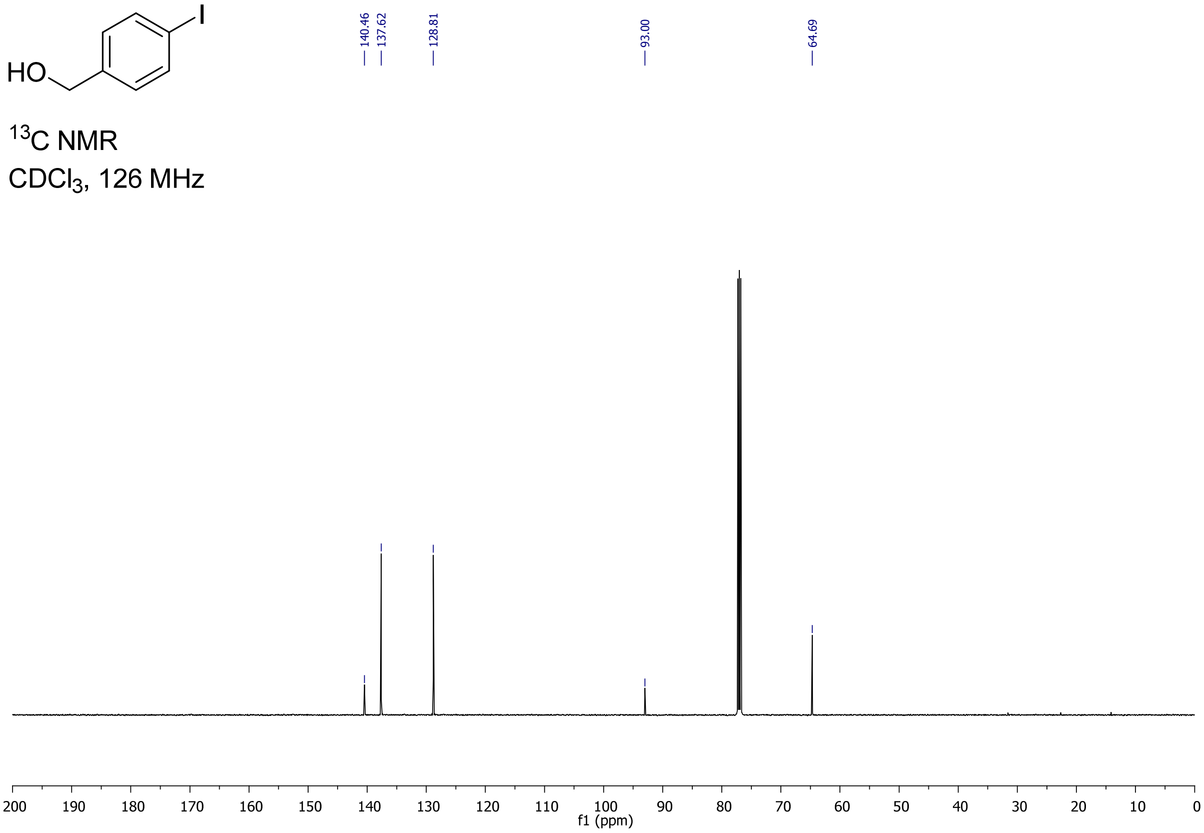

^{13}C NMR
$CDCl_3$, 126 MHz

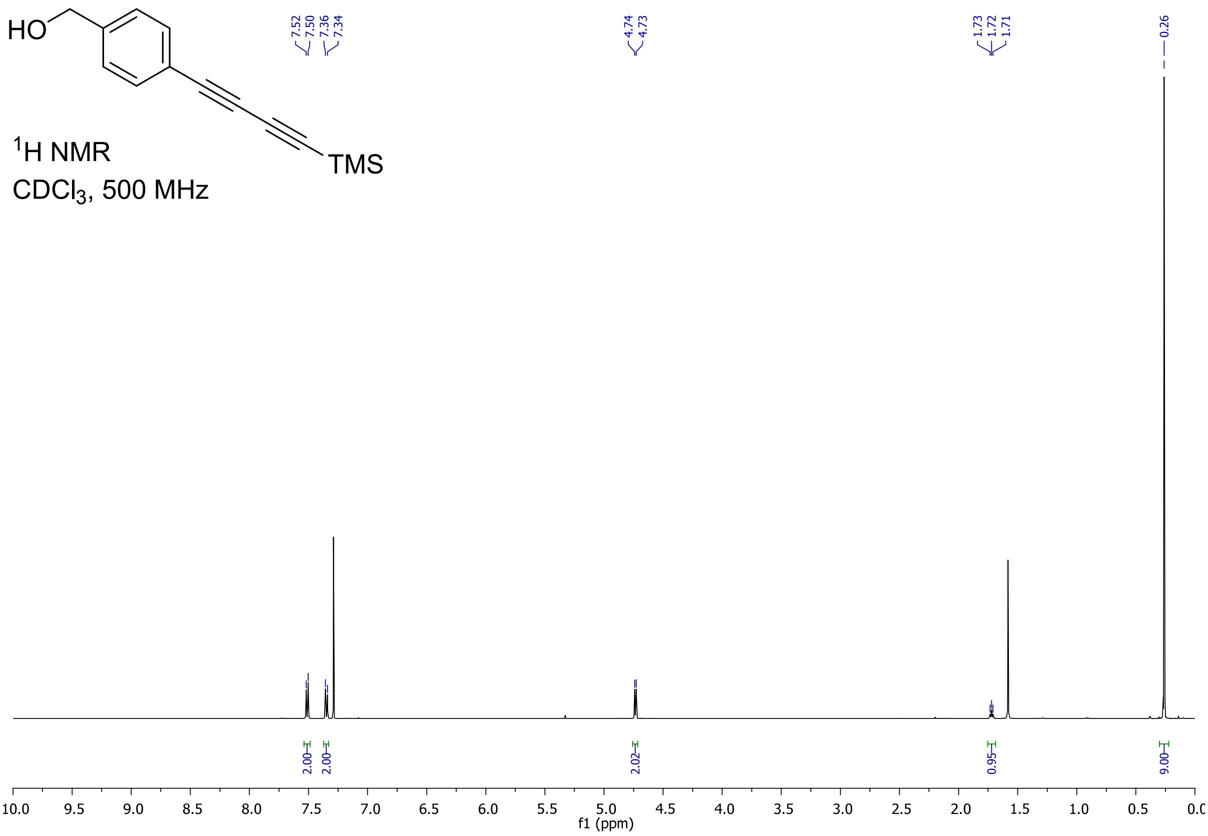
HO
TMS
1H NMR
CDCl3, 500 MHz
7.52
7.50
7.36
7.34
4.74
4.73
1.73
1.72
1.71
0.26
2.00
2.00
2.02
0.95
9.00
f1 (ppm)

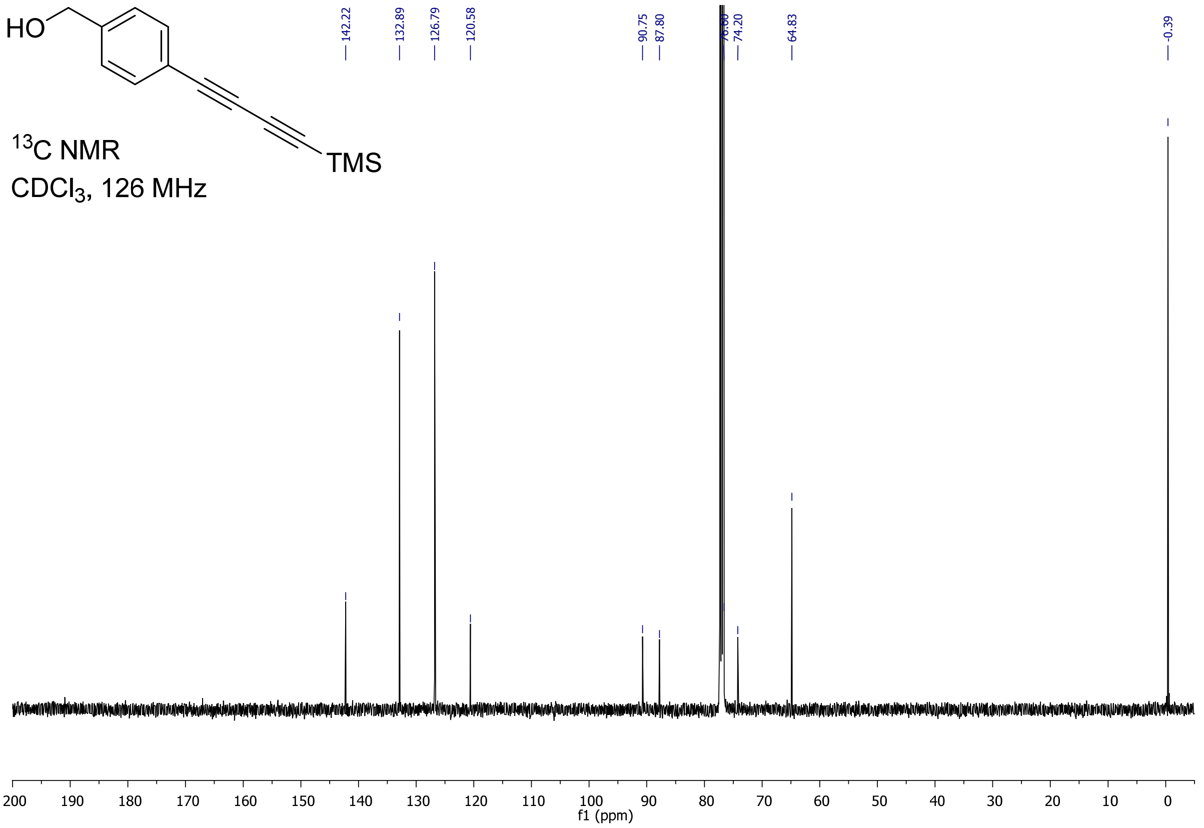
HO
TMS
13C NMR
CDCl3, 126 MHz
142.22
132.89
126.79
120.58
90.75
87.80
74.20
64.83
-0.39
f1 (ppm)

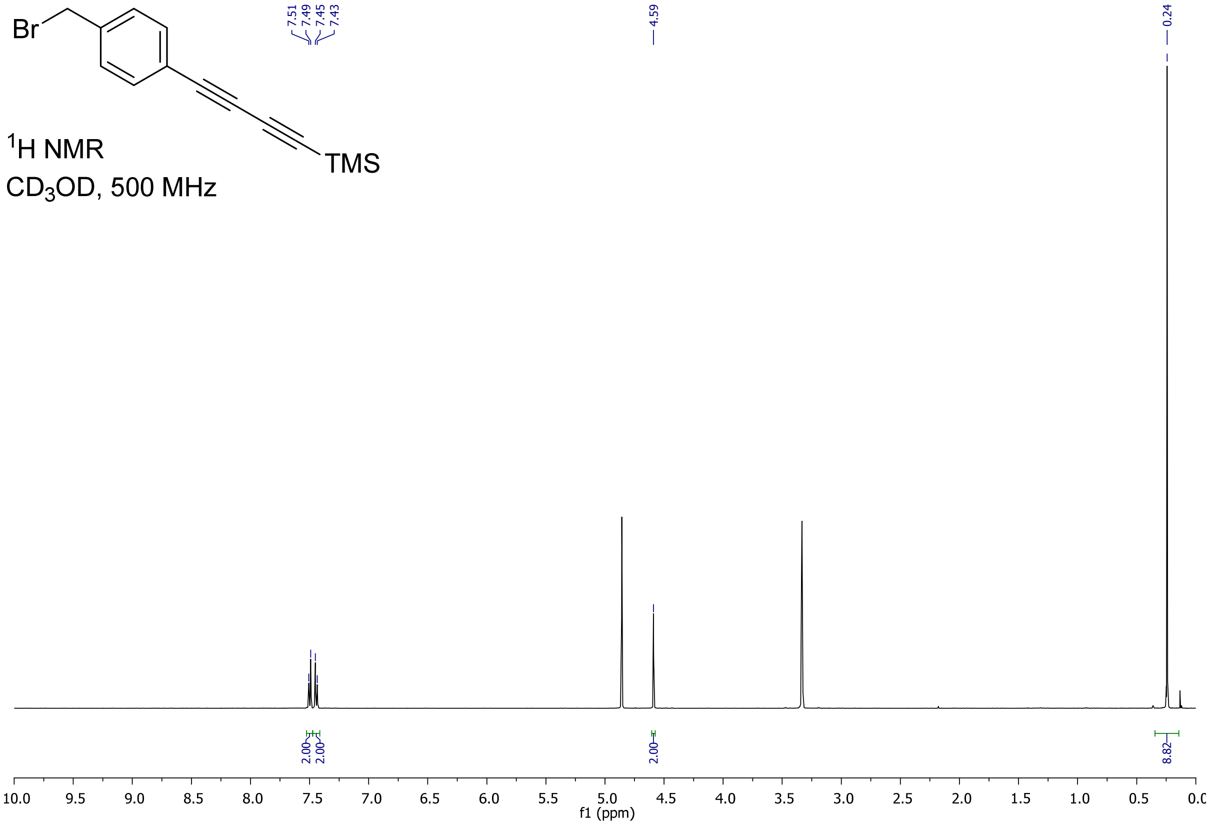
Br
TMS
^{1}H NMR
CD$_3$OD, 500 MHz
7.51
7.49
7.45
7.43
4.59
0.24
2.00
2.00
2.00
8.82
f1 (ppm)

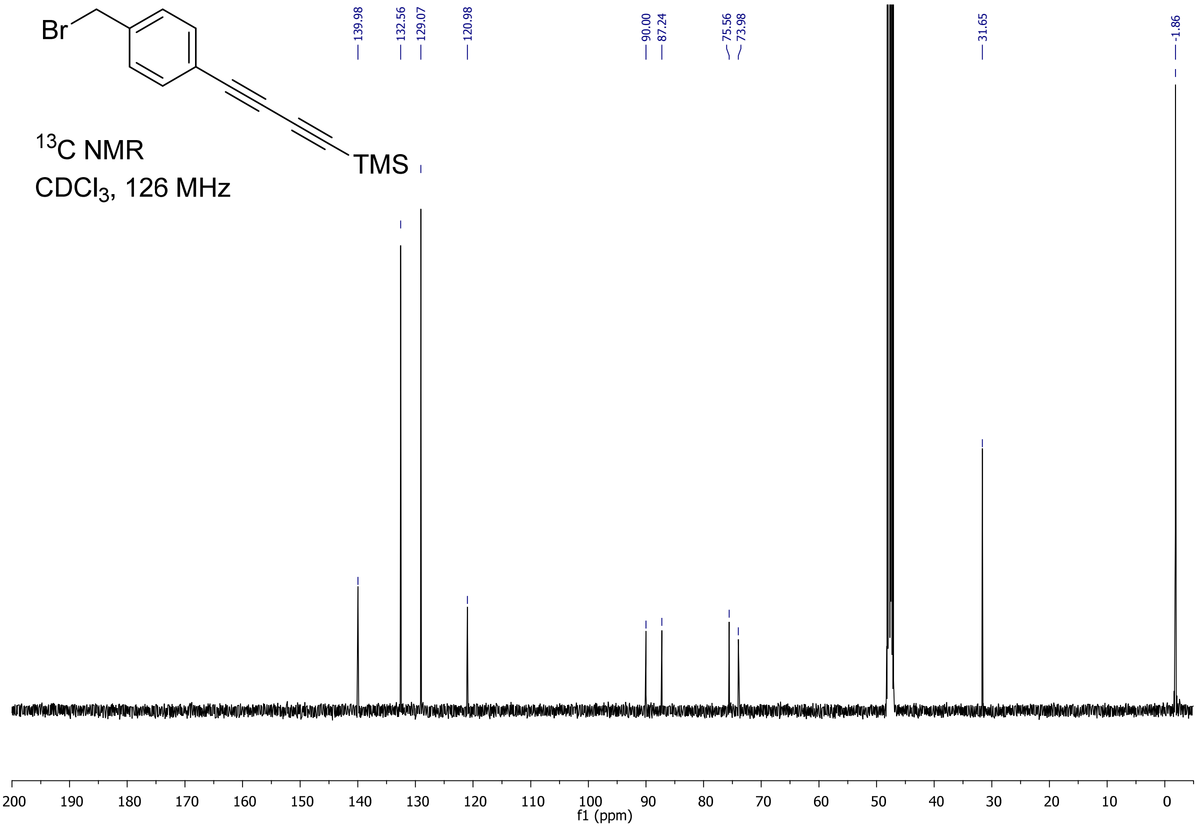
Br
TMS
^{13}C NMR
CDCl$_3$, 126 MHz
139.98
132.56
129.07
120.98
90.00
87.24
75.56
73.98
31.65
-1.86
f1 (ppm)

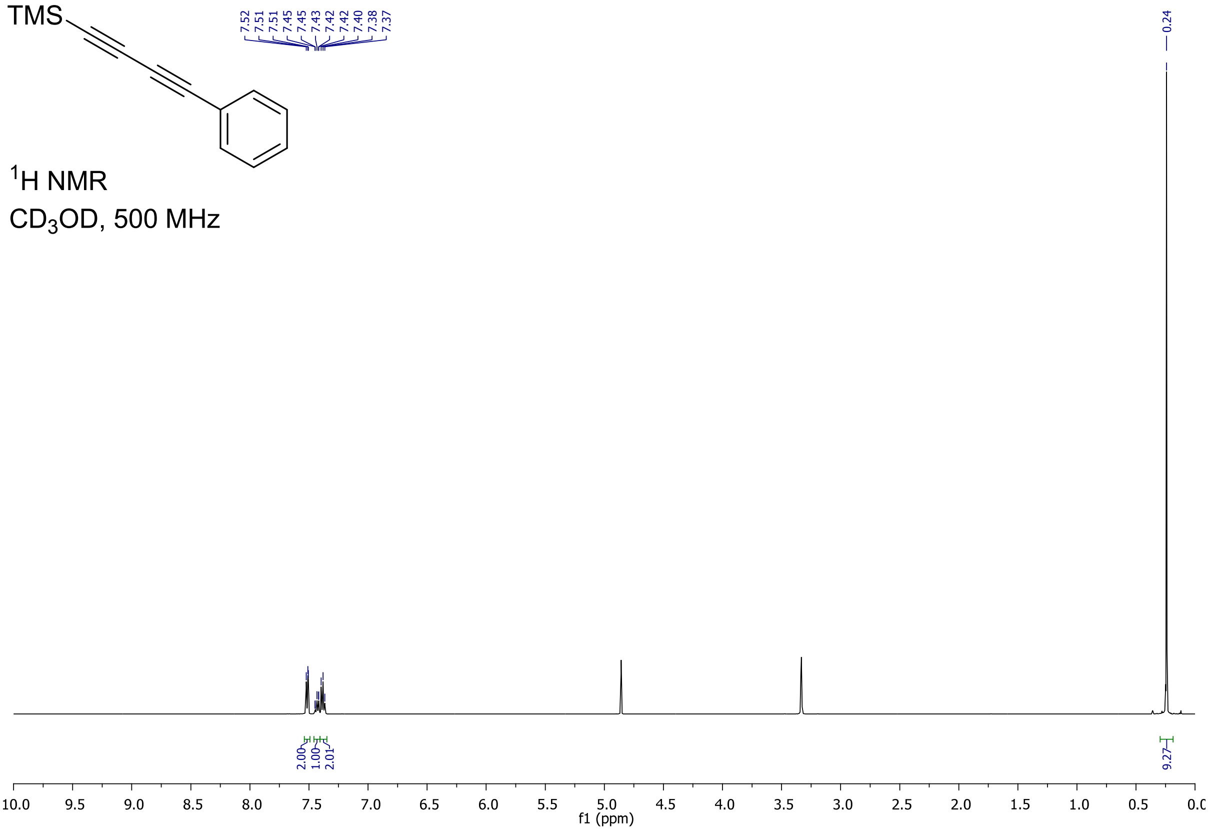
TMS
1H NMR
CD3OD, 500 MHz
7.52
7.51
7.51
7.45
7.45
7.43
7.42
7.42
7.40
7.38
7.37
0.24
2.00
1.00
2.01
9.27
f1 (ppm)

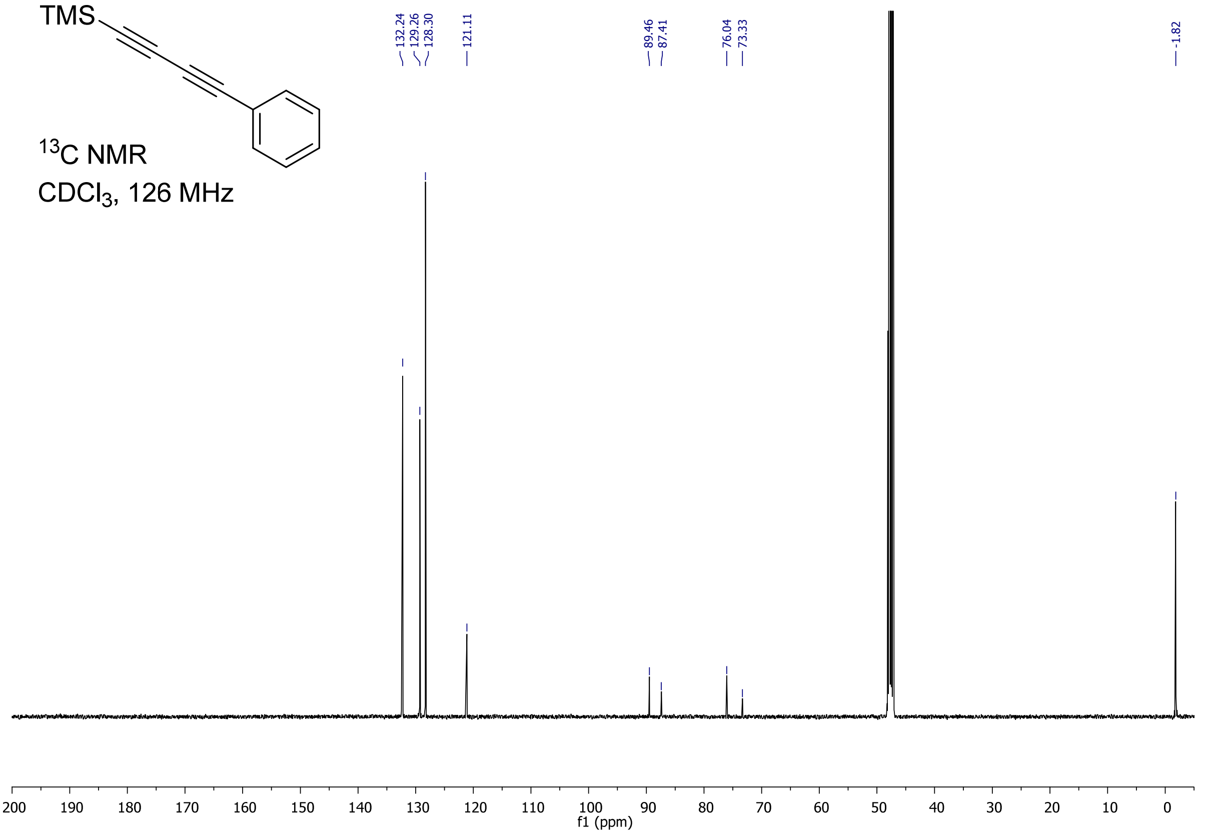
TMS
13C NMR
CDCl3, 126 MHz
132.24
129.26
128.30
121.11
89.46
87.41
76.04
73.33
-1.82
f1 (ppm)

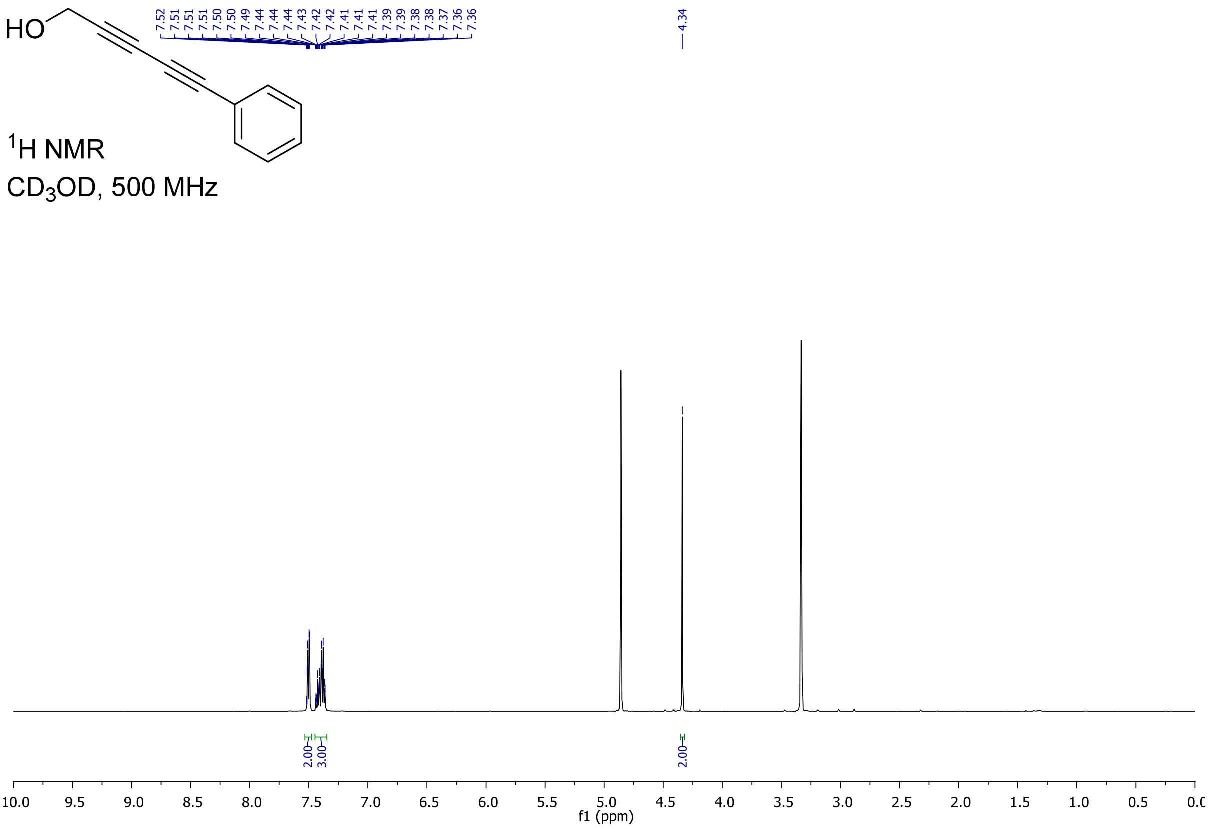
HO
7.52 7.51 7.51 7.51 7.50 7.49 7.44 7.44 7.44 7.43 7.42 7.42 7.41 7.41 7.41 7.39 7.39 7.38 7.38 7.37 7.36 7.36
4.34
1H NMR
CD3OD, 500 MHz
2.00
3.00
2.00
10.0 9.5 9.0 8.5 8.0 7.5 7.0 6.5 6.0 5.5 5.0 4.5 4.0 3.5 3.0 2.5 2.0 1.5 1.0 0.5 0.0
f1 (ppm)

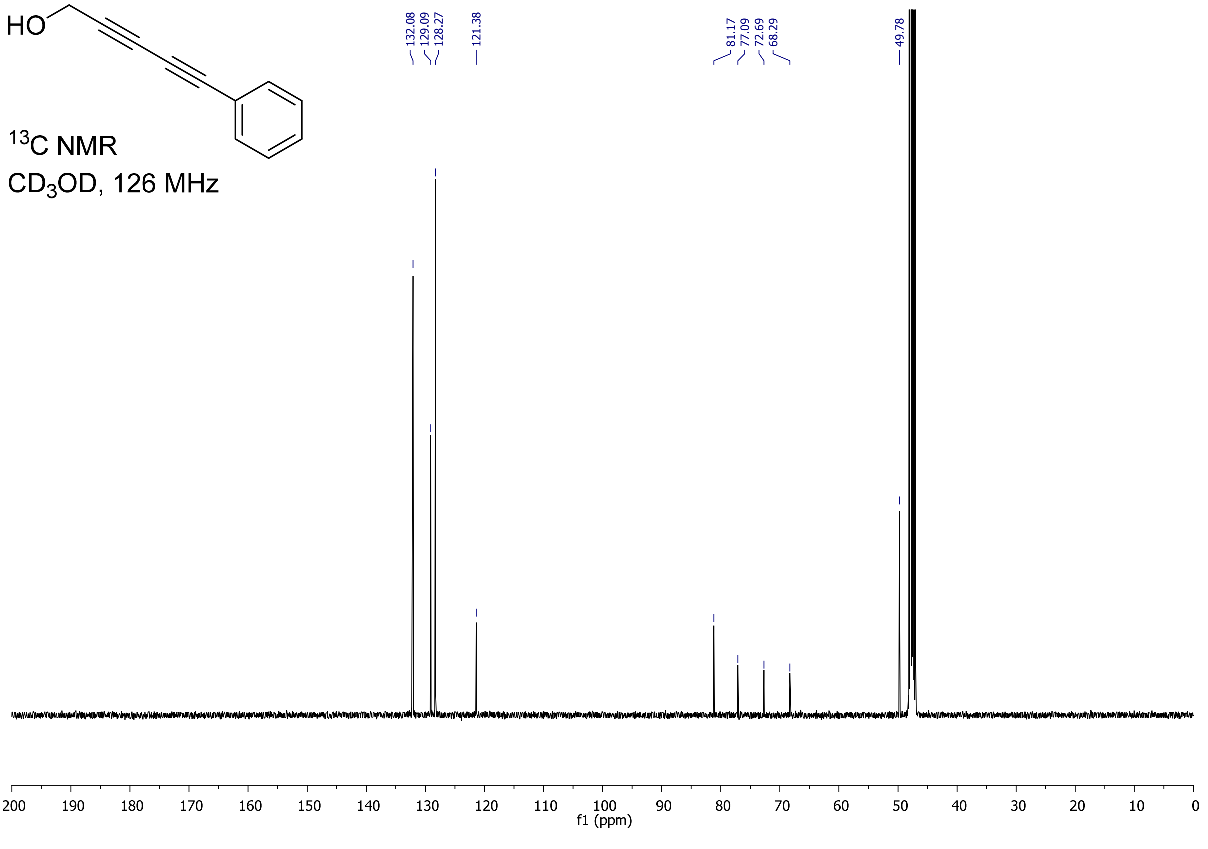
HO
132.08
129.09
128.27
121.38
81.17
77.09
72.69
68.29
49.78
13C NMR
CD3OD, 126 MHz
200 190 180 170 160 150 140 130 120 110 100 90 80 70 60 50 40 30 20 10 0
f1 (ppm)

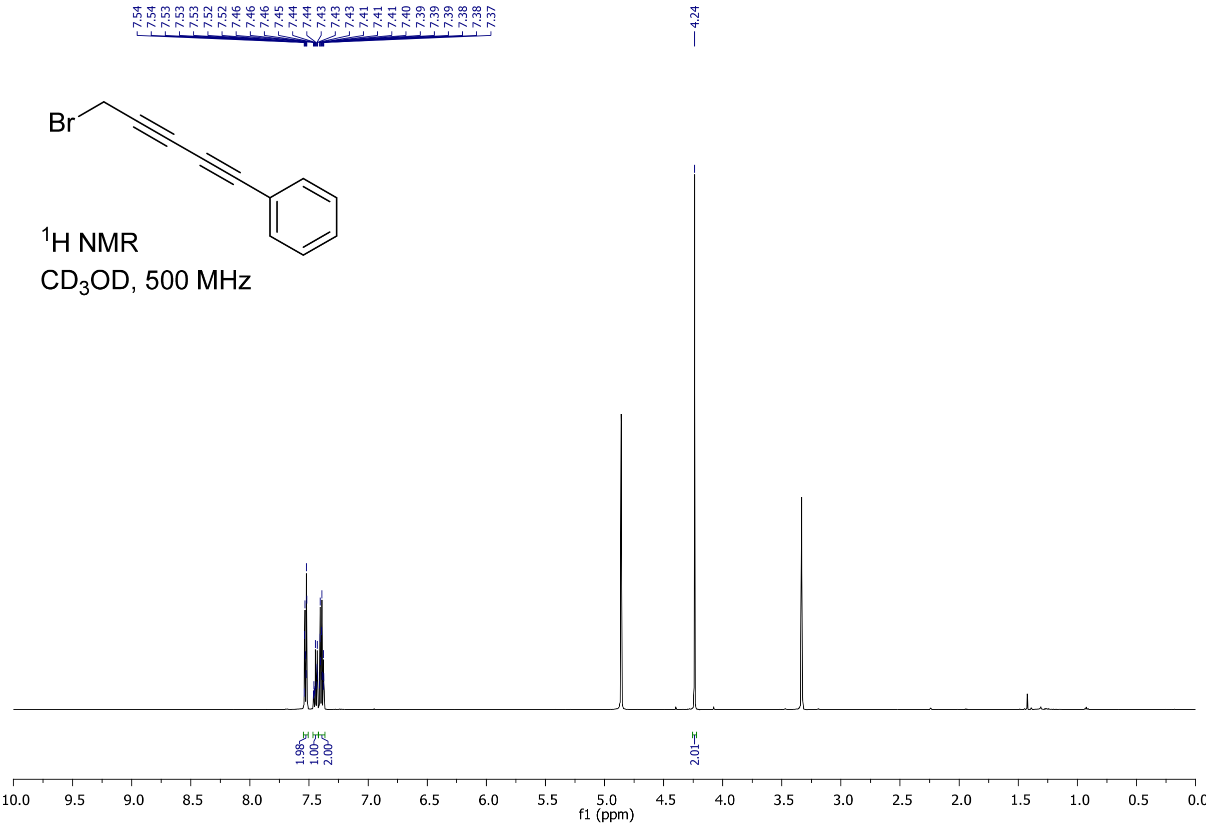
Br
^{1}H NMR
CD_3OD, 500 MHz
7.54 7.54 7.53 7.53 7.53 7.52 7.52 7.46 7.46 7.46 7.45 7.44 7.44 7.43 7.43 7.43 7.41 7.41 7.41 7.40 7.39 7.39 7.39 7.38 7.38 7.37
4.24
1.98 1.00 2.00
2.01
10.0 9.5 9.0 8.5 8.0 7.5 7.0 6.5 6.0 5.5 5.0 4.5 4.0 3.5 3.0 2.5 2.0 1.5 1.0 0.5 0.0
f1 (ppm)

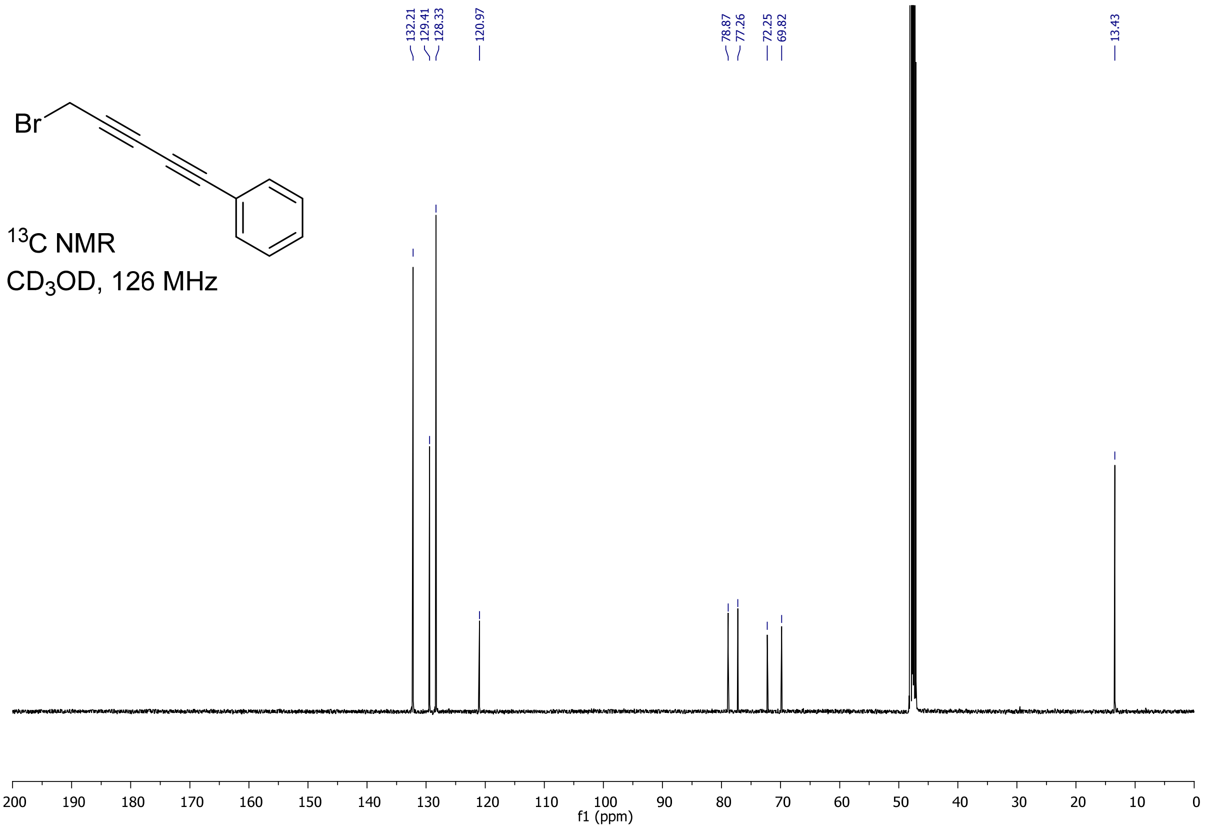
Br
^{13}C NMR
CD_3OD, 126 MHz
132.21 129.41 128.33
120.97
78.87 77.26
72.25 69.82
13.43
200 190 180 170 160 150 140 130 120 110 100 90 80 70 60 50 40 30 20 10 0
f1 (ppm)

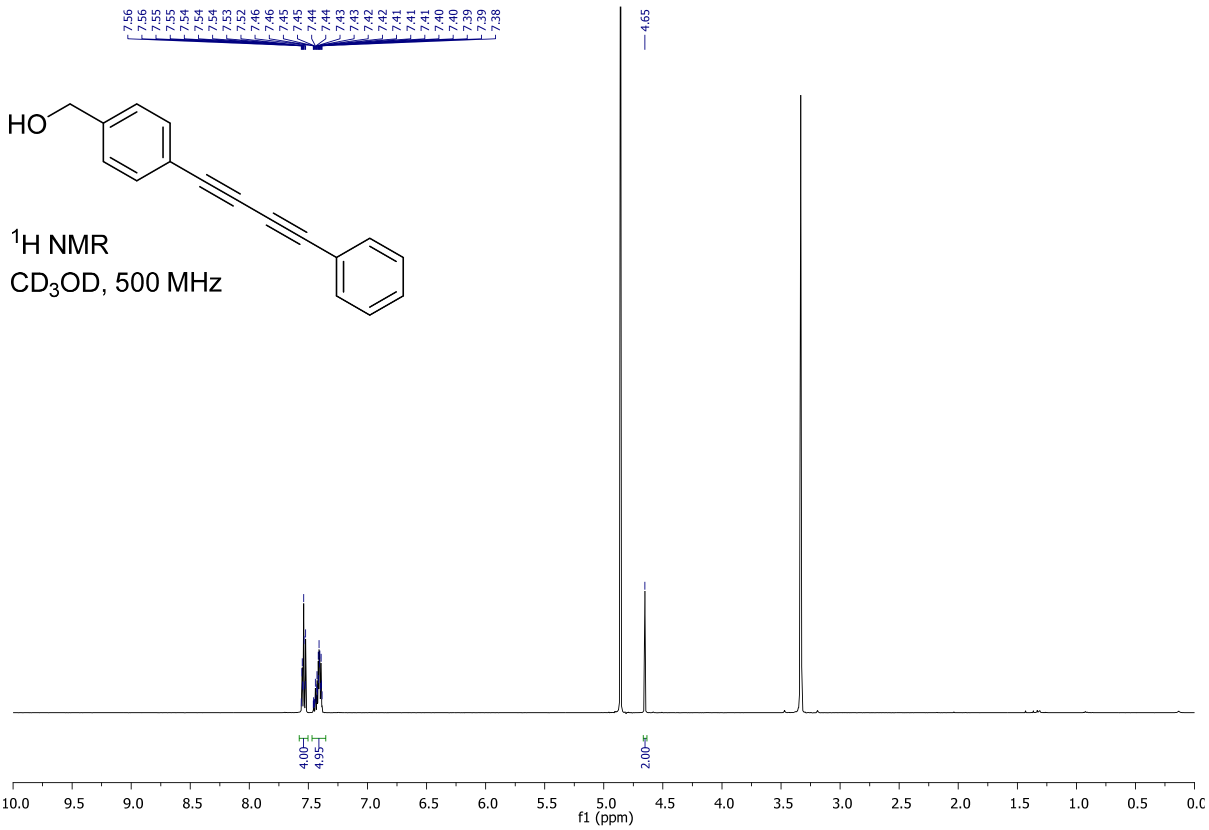
HO
1H NMR
CD3OD, 500 MHz
7.56
7.56
7.55
7.55
7.54
7.54
7.54
7.53
7.52
7.46
7.46
7.45
7.45
7.44
7.44
7.43
7.43
7.42
7.42
7.41
7.41
7.41
7.40
7.40
7.39
7.39
7.38
4.65
4.00
4.95
2.00
f1 (ppm)

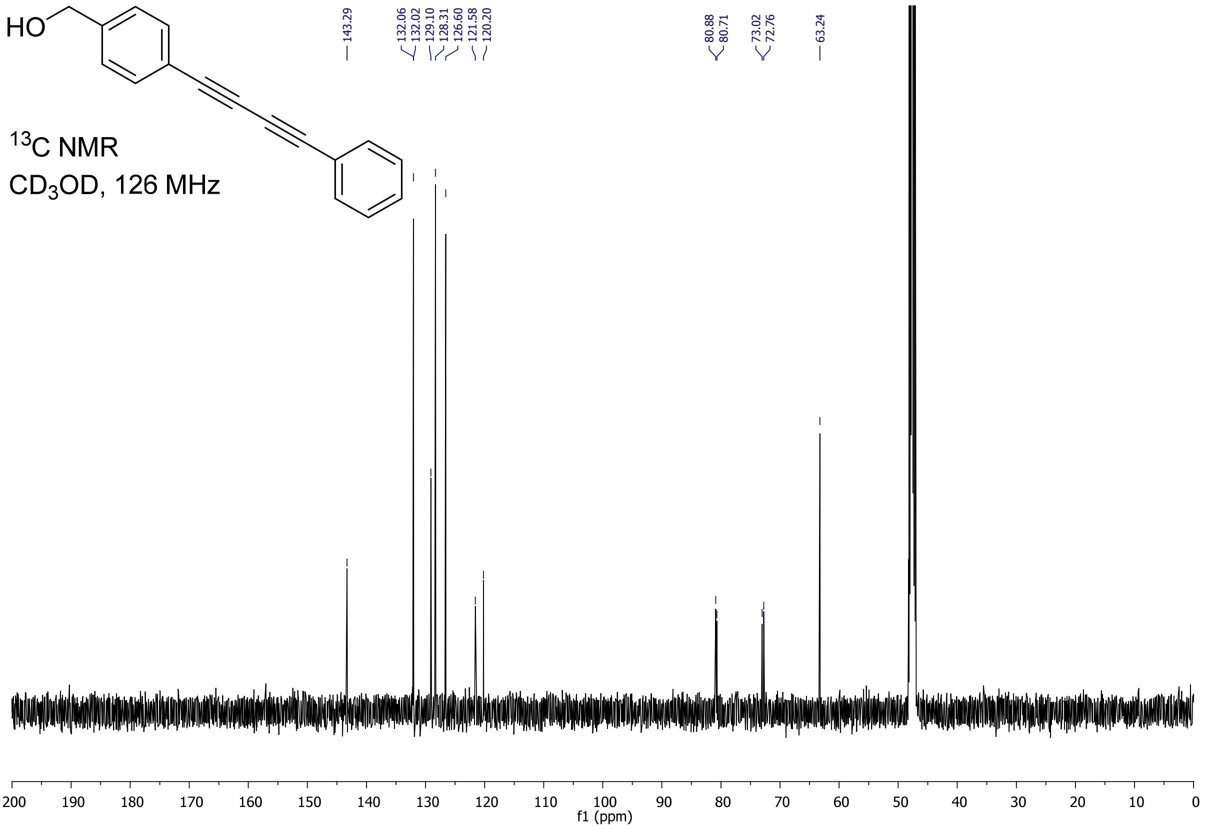
HO
13C NMR
CD3OD, 126 MHz
143.29
132.06
132.02
129.10
128.31
126.60
121.58
120.20
80.88
80.71
73.02
72.76
63.24
f1 (ppm)

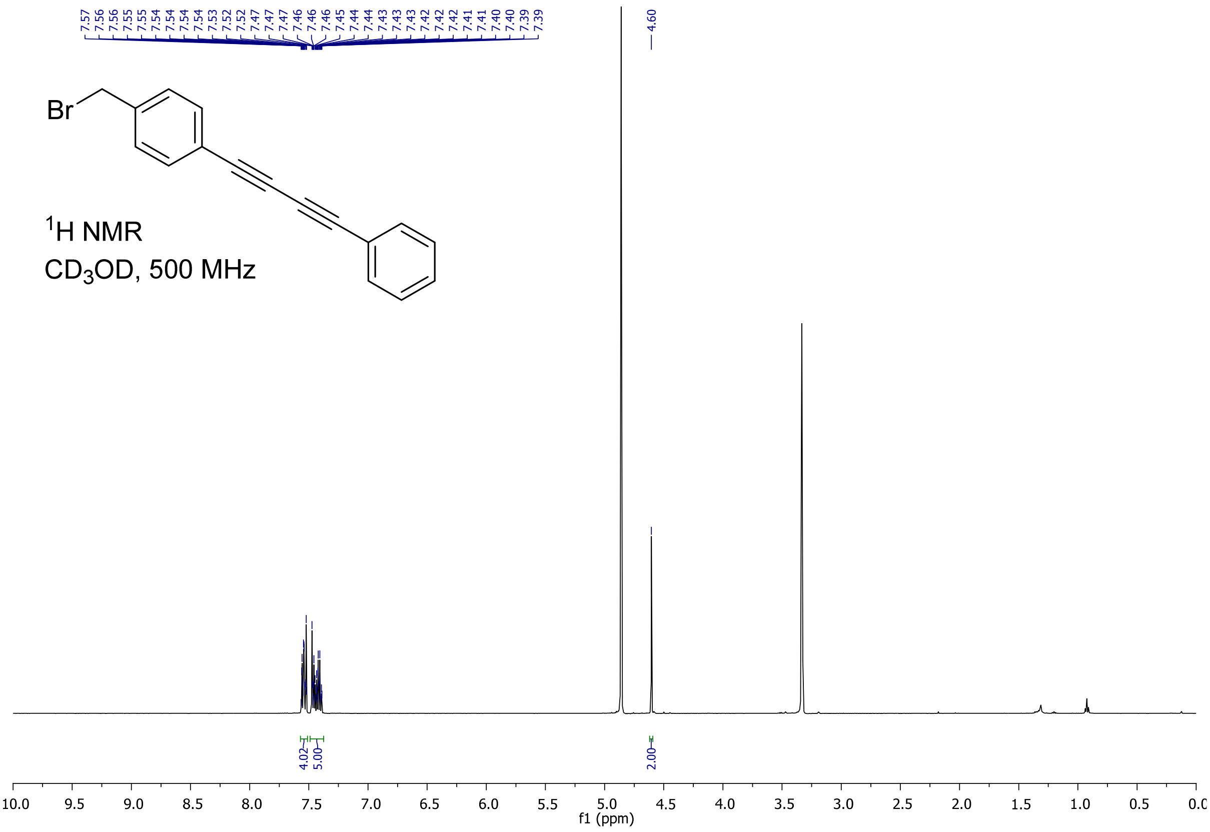

7.57 7.56 7.56 7.55 7.54 7.54 7.54 7.54 7.53 7.52 7.52 7.47 7.47 7.47 7.46 7.46 7.46 7.45 7.44 7.44 7.43 7.43 7.42 7.42 7.42 7.41 7.41 7.40 7.40 7.39 7.39
4.60
Br
1H NMR
CD3OD, 500 MHz
4.02
5.00
2.00
f1 (ppm)

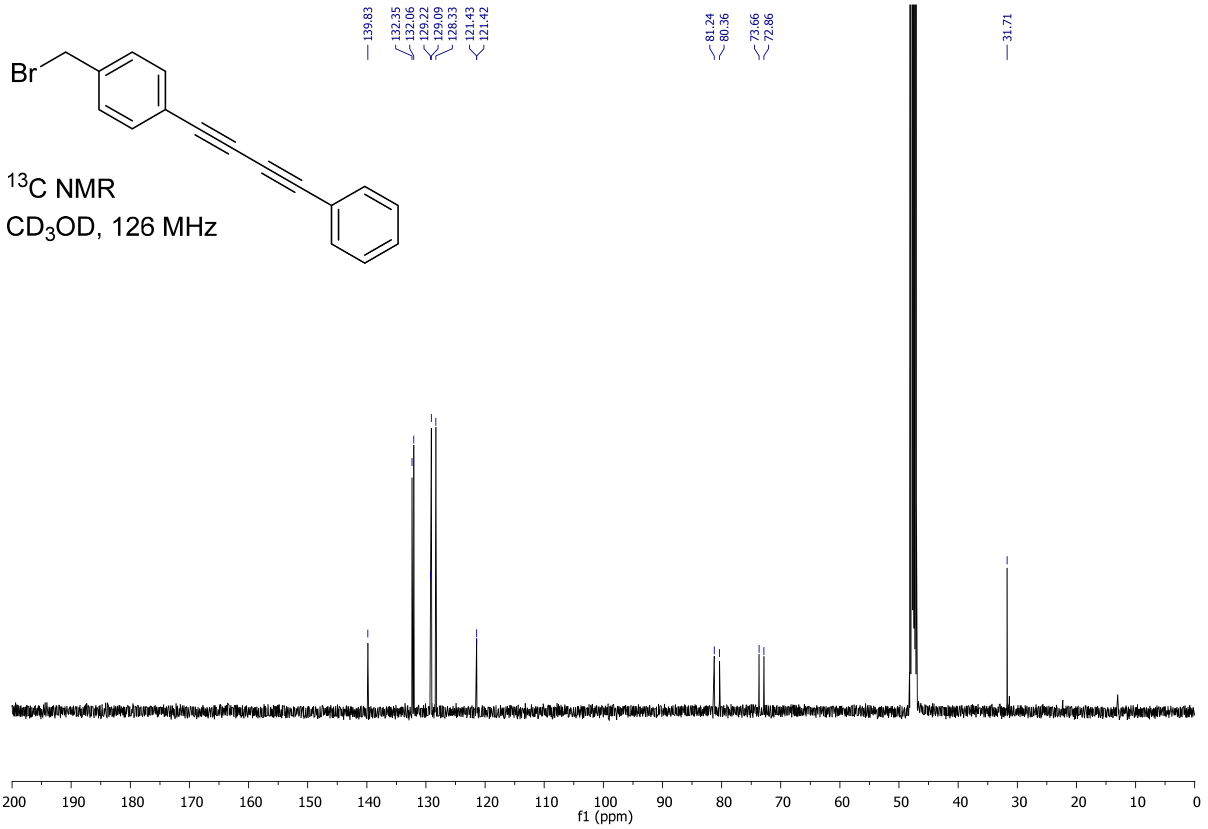

139.83
132.35 132.06 129.22 129.09 128.33 121.43 121.42
81.24 80.36
73.66 72.86
31.71
Br
13C NMR
CD3OD, 126 MHz
f1 (ppm)

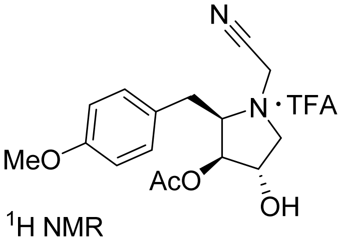

^{1}H NMR

$CDCl_3$, 500 MHz

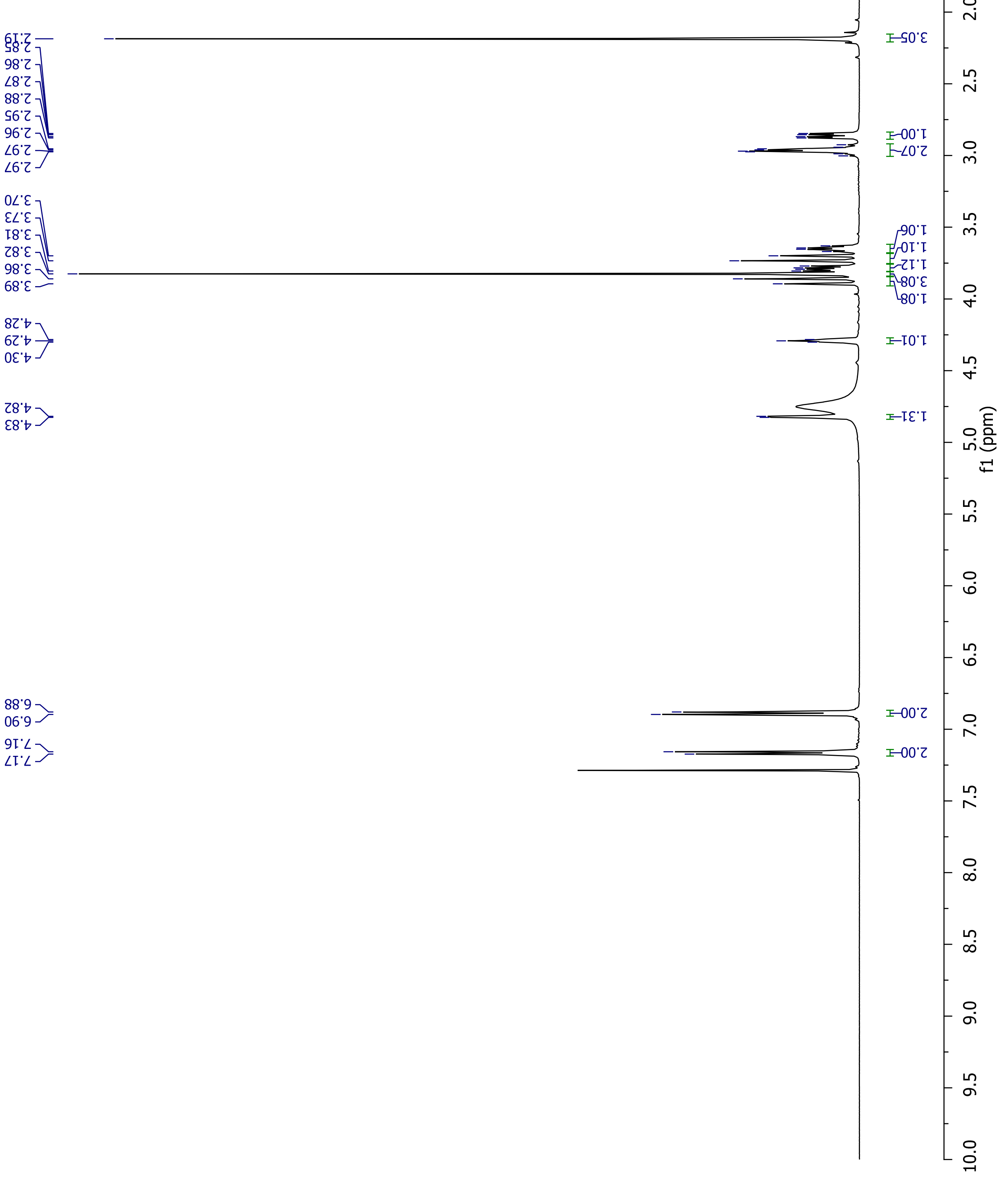

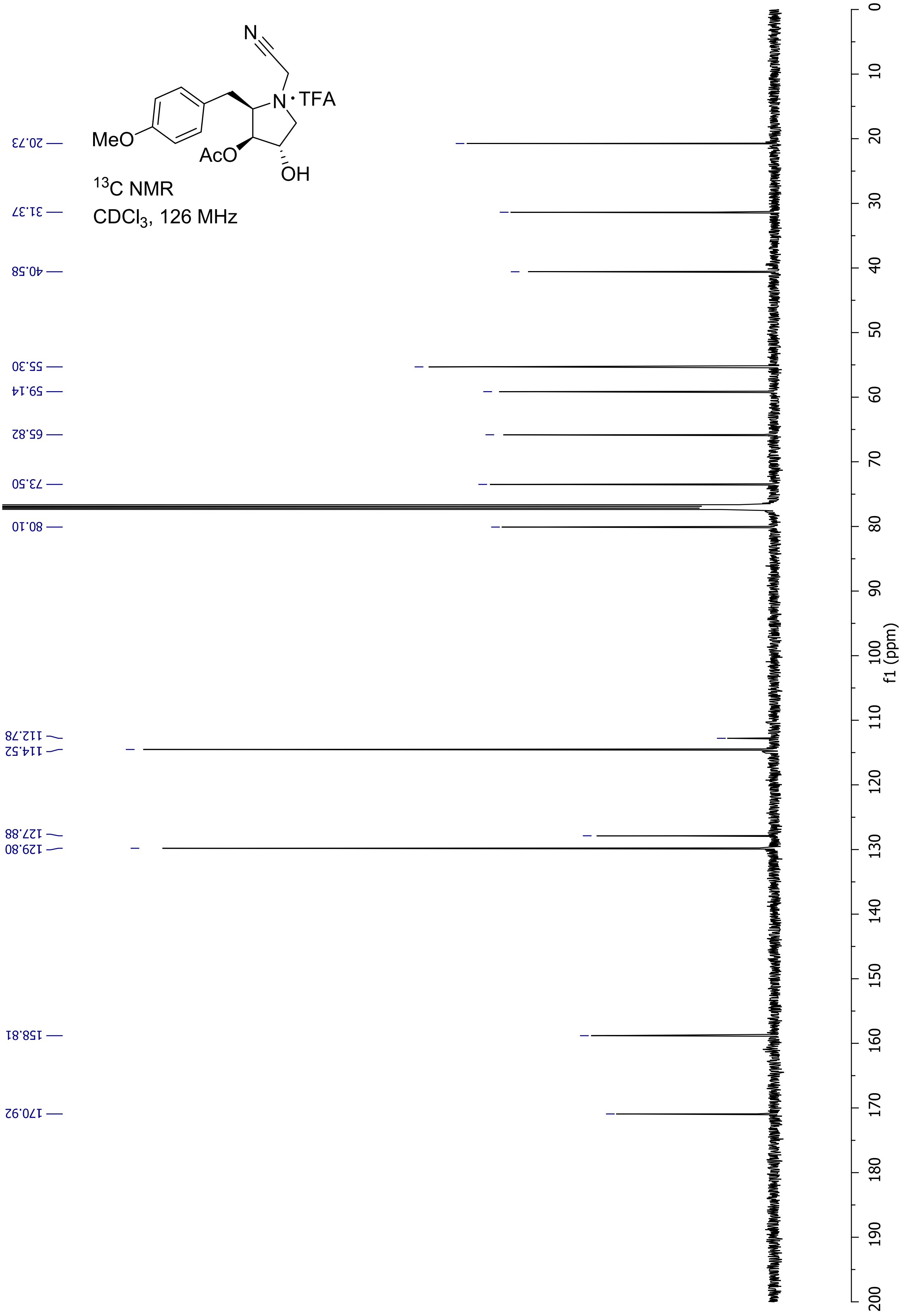

N
N·TFA
MeO
AcO
OH
^{13}C NMR
$CDCl_3$, 126 MHz
20.73
31.37
40.58
55.30
59.14
65.82
73.50
80.10
112.78
114.52
127.88
129.80
158.81
170.92
f1 (ppm)

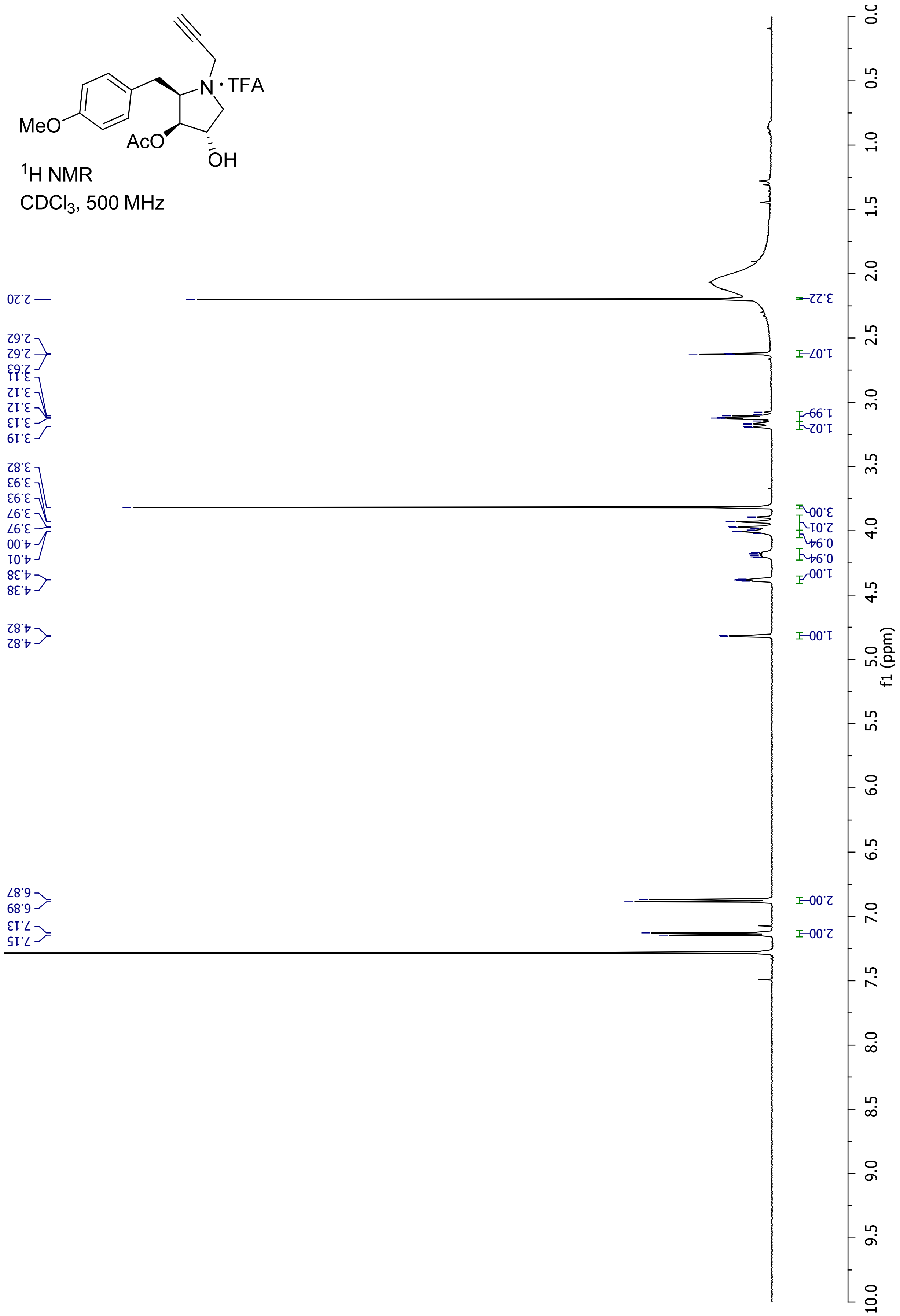

MeO
AcO
OH
N
· TFA
^{1}H NMR
$CDCl_3$, 500 MHz
f1 (ppm)

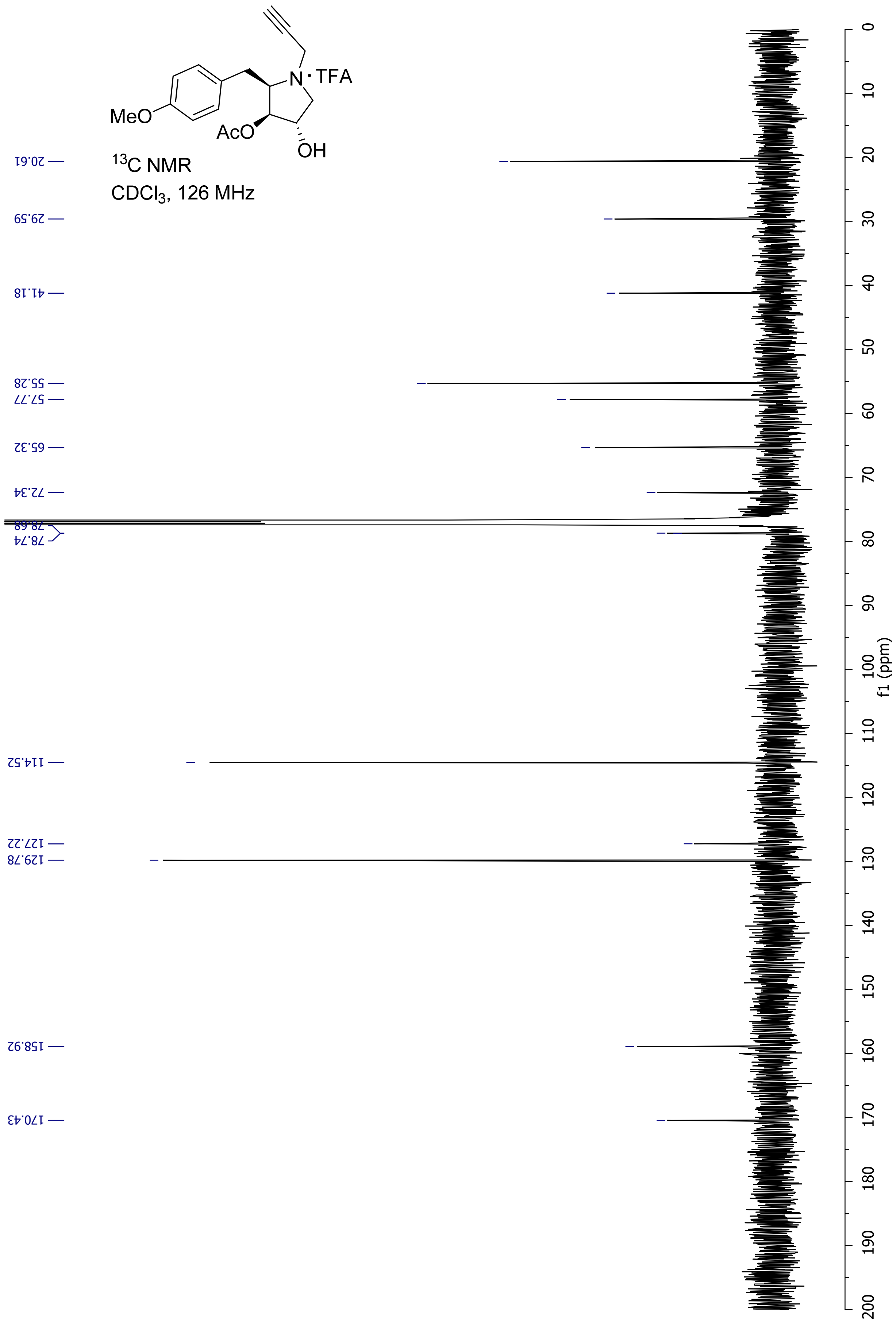

MeO
AcO
OH
N·TFA
^{13}C NMR
$CDCl_3$, 126 MHz
20.61
29.59
41.18
55.28
57.77
65.32
72.34
78.68
78.74
114.52
127.22
129.78
158.92
170.43
f1 (ppm)

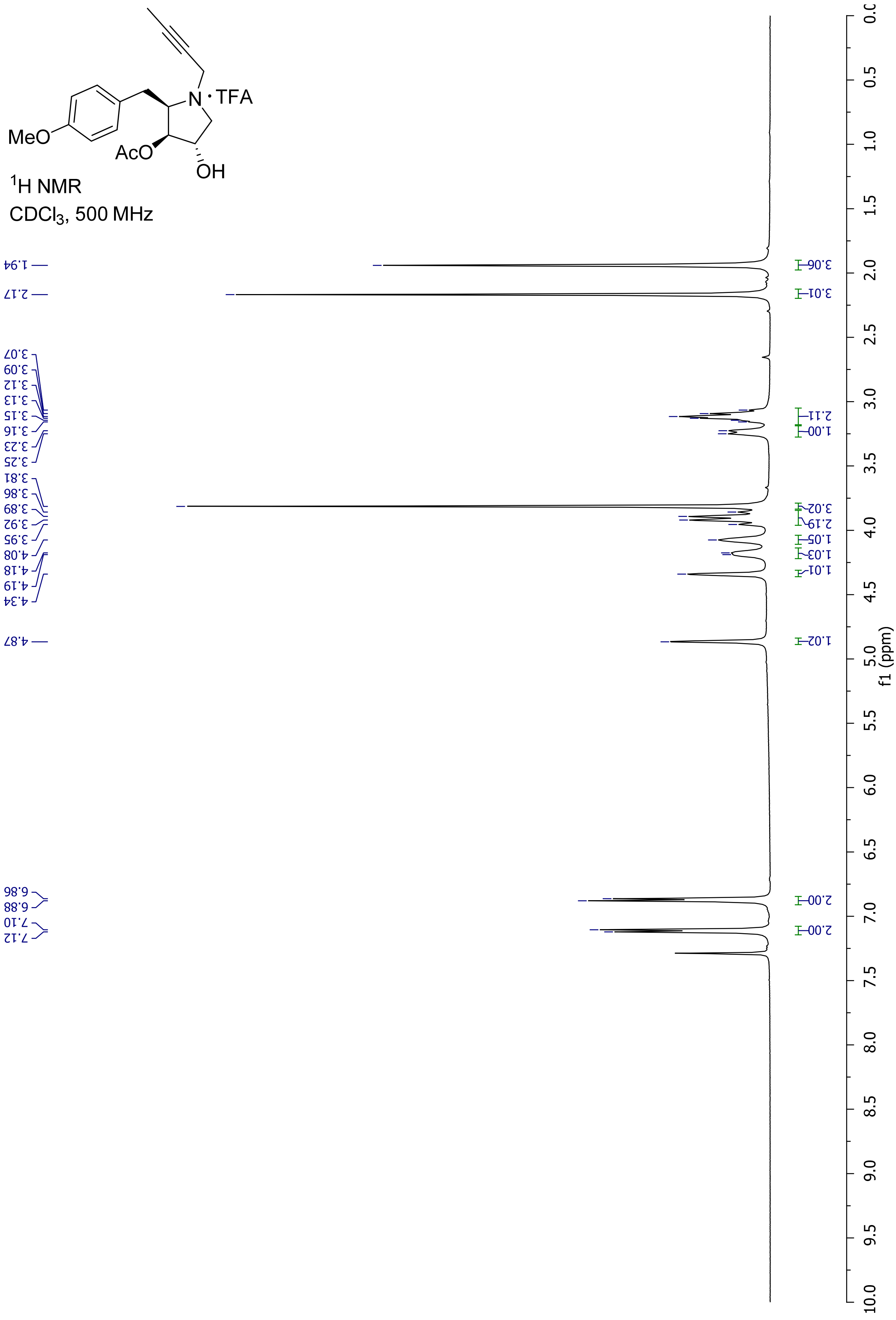

MeO
AcO
OH
N · TFA
1H NMR
CDCl3, 500 MHz
1.94
2.17
3.07
3.09
3.12
3.13
3.15
3.16
3.23
3.25
3.81
3.86
3.89
3.92
3.95
4.08
4.18
4.19
4.34
4.87
6.86
6.88
7.10
7.12
3.06
3.01
2.11
1.00
3.02
2.19
1.05
1.03
1.01
1.02
2.00
2.00
f1 (ppm)

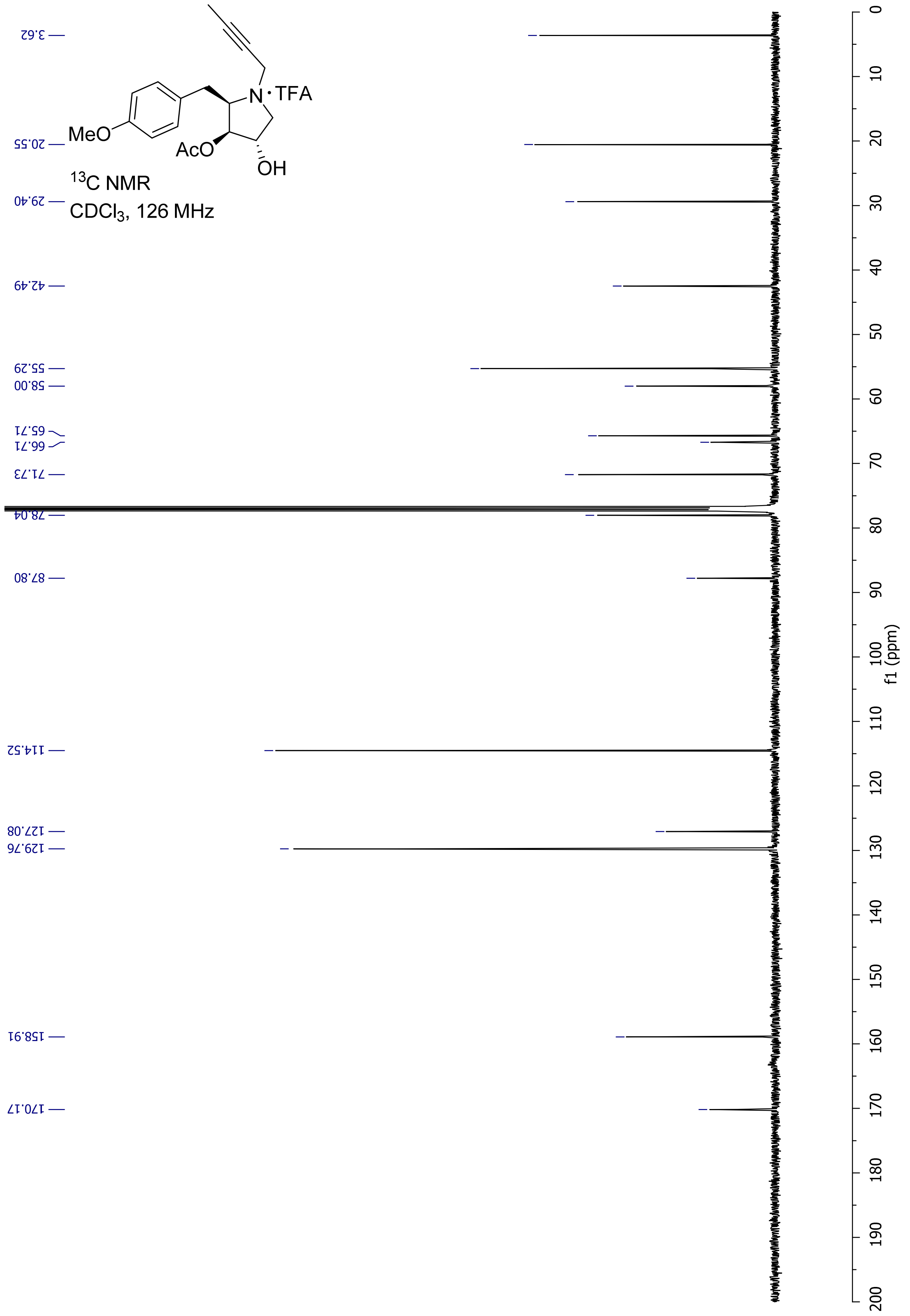

MeO
AcO
OH
N·TFA
13C NMR
CDCl3, 126 MHz
3.62
20.55
29.40
42.49
55.29
58.00
65.71
66.71
71.73
78.04
87.80
114.52
127.08
129.76
158.91
170.17
f1 (ppm)

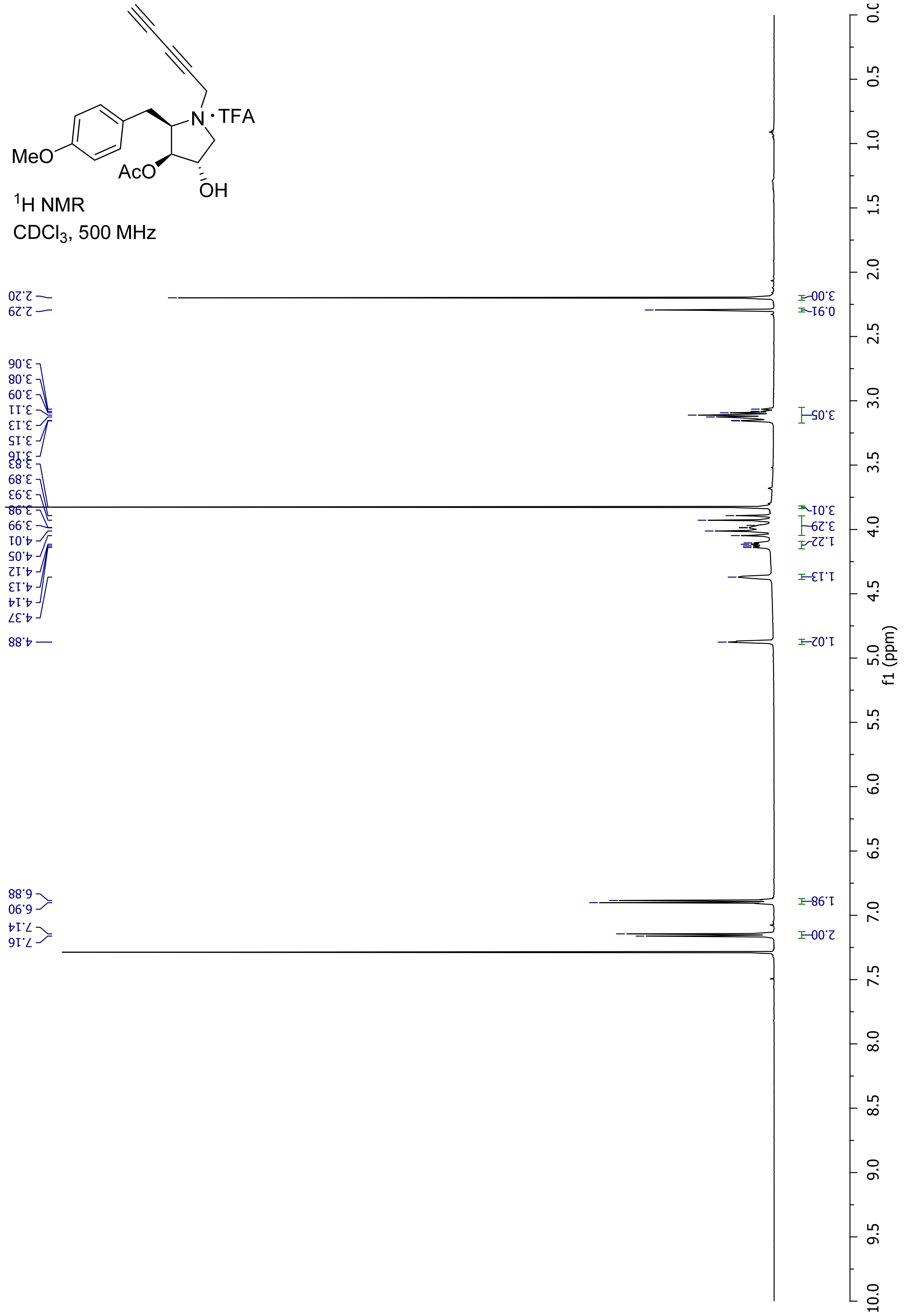

^{1}H NMR

$CDCl_3$, 500 MHz

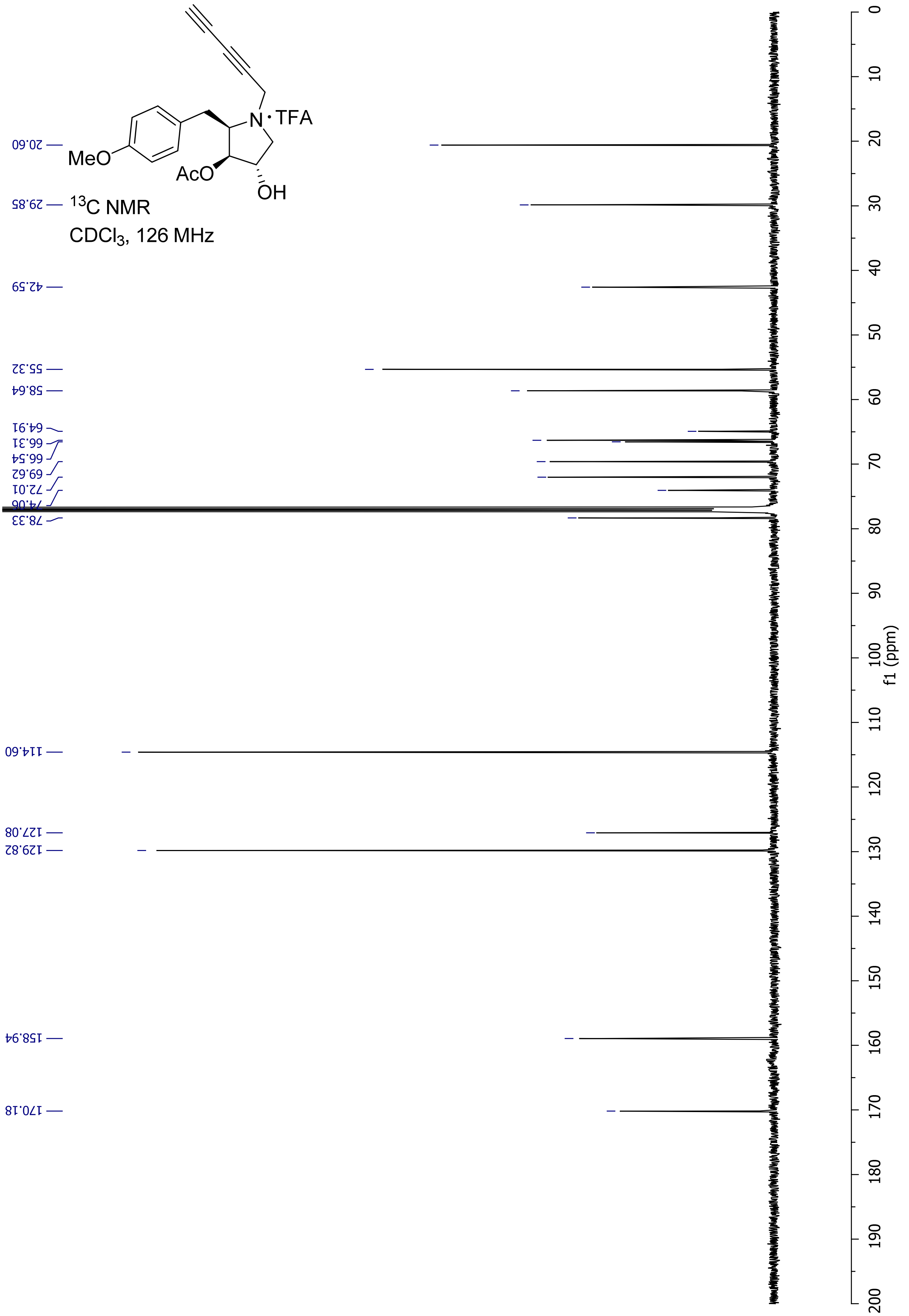
N · TFA
MeO
AcO
OH
^{13}C NMR
$CDCl_3$, 126 MHz
20.60
29.85
42.59
55.32
58.64
64.91
66.31
66.54
69.62
72.01
74.06
78.33
114.60
127.08
129.82
158.94
170.18
f1 (ppm)

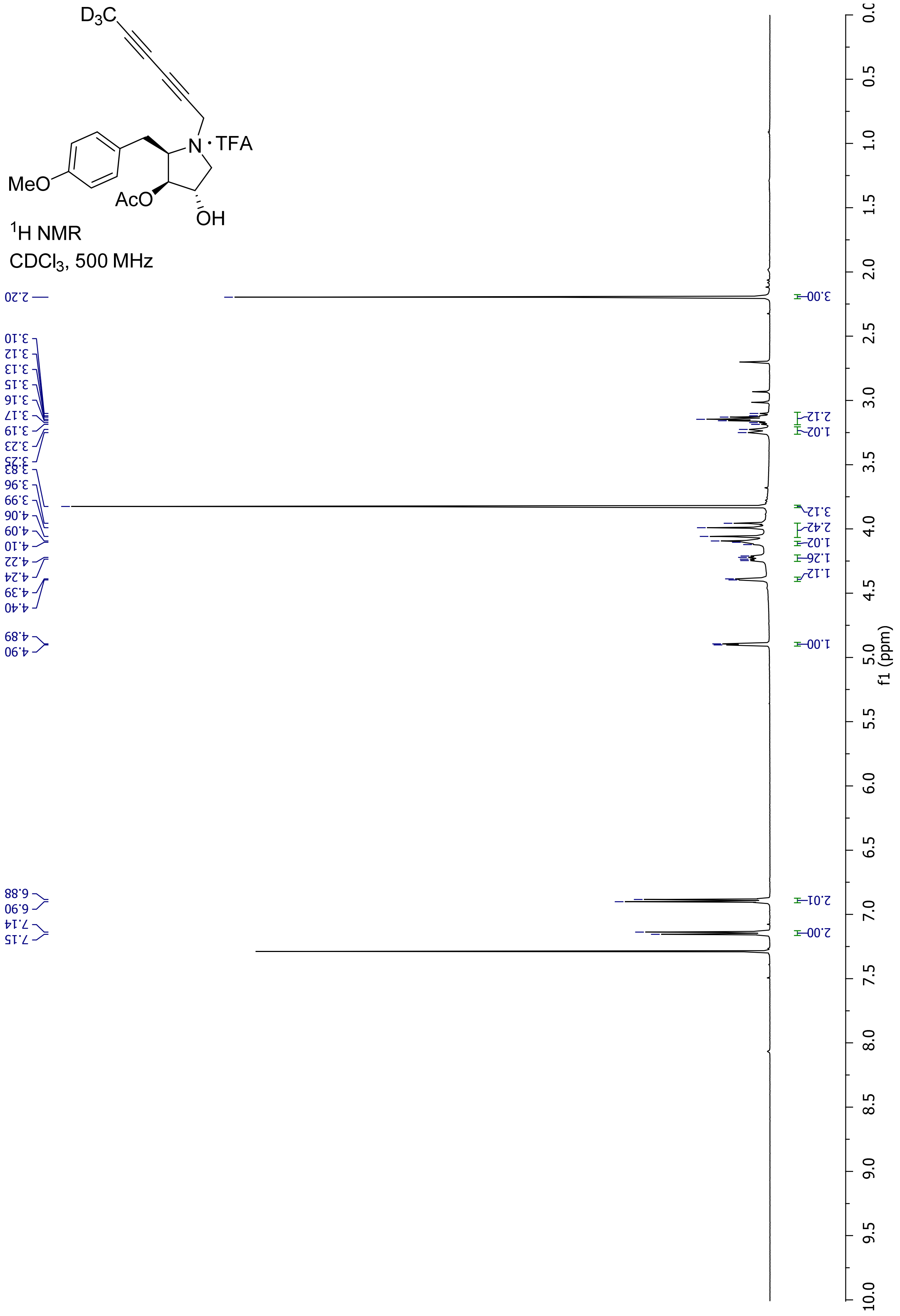
D3C
N · TFA
MeO
AcO
OH
1H NMR
CDCl3, 500 MHz
2.20
3.10
3.12
3.13
3.15
3.16
3.17
3.19
3.23
3.25
3.83
3.96
3.99
4.06
4.09
4.10
4.22
4.24
4.39
4.40
4.89
4.90
6.88
6.90
7.14
7.15
3.00
2.12
1.02
3.12
2.42
1.02
1.26
1.12
1.00
2.01
2.00
f1 (ppm)

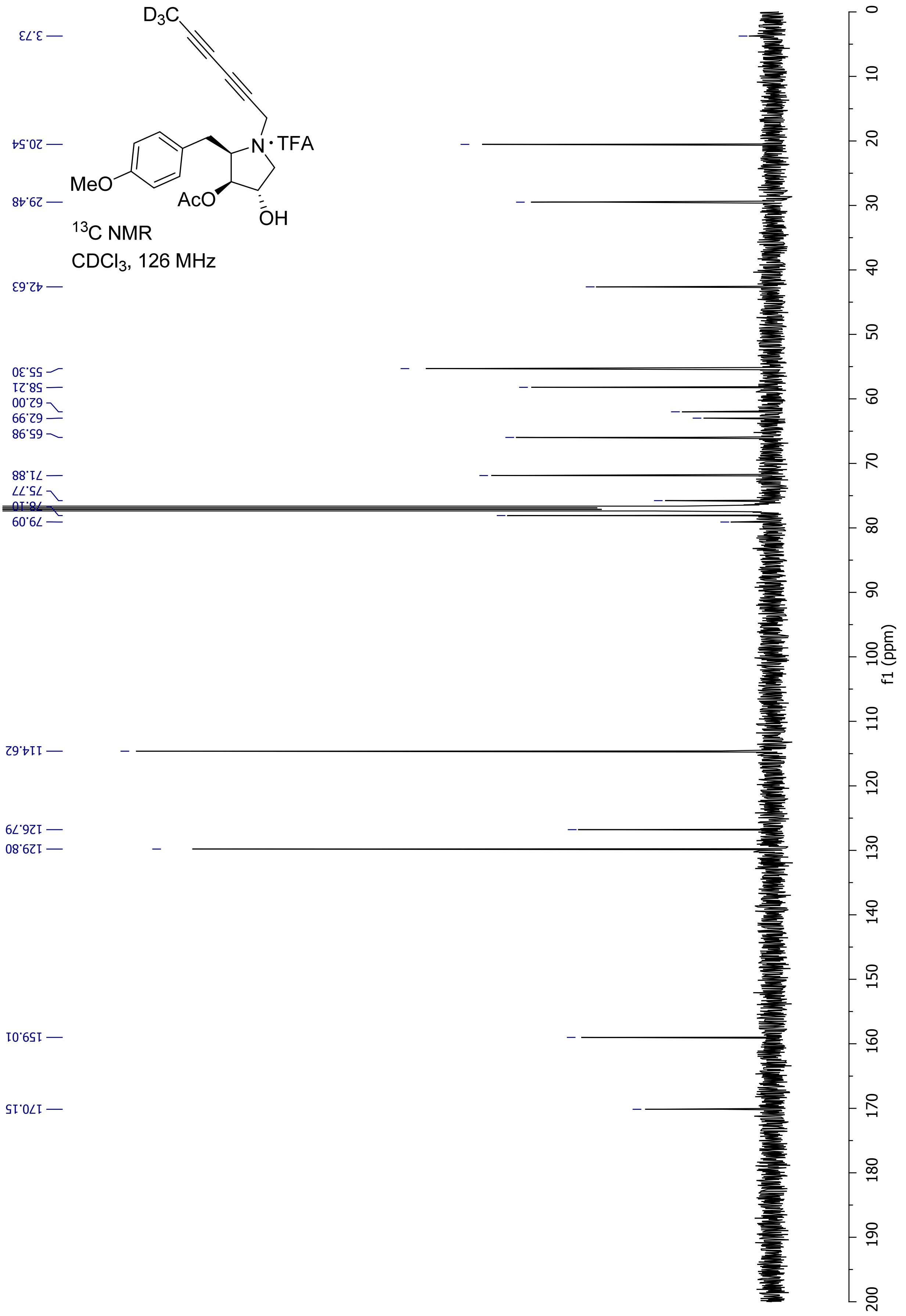
D3C
N · TFA
MeO
AcO
OH
13C NMR
CDCl3, 126 MHz
3.73
20.54
29.48
42.63
55.30
58.21
62.00
62.99
65.98
71.88
75.77
78.10
79.09
114.62
126.79
129.80
159.01
170.15
f1 (ppm)

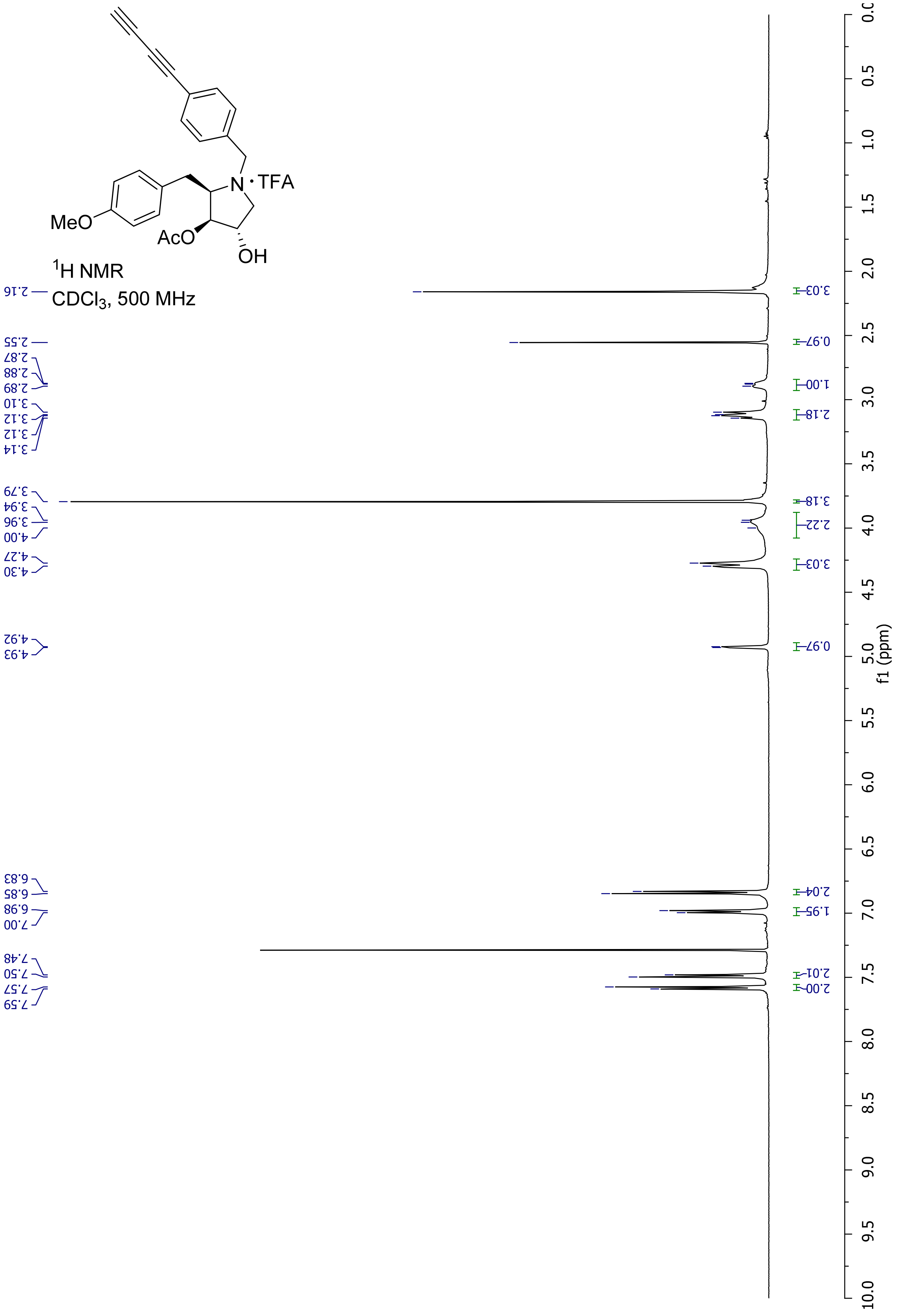
TFA
MeO
AcO
OH
1H NMR
CDCl3, 500 MHz
f1 (ppm)

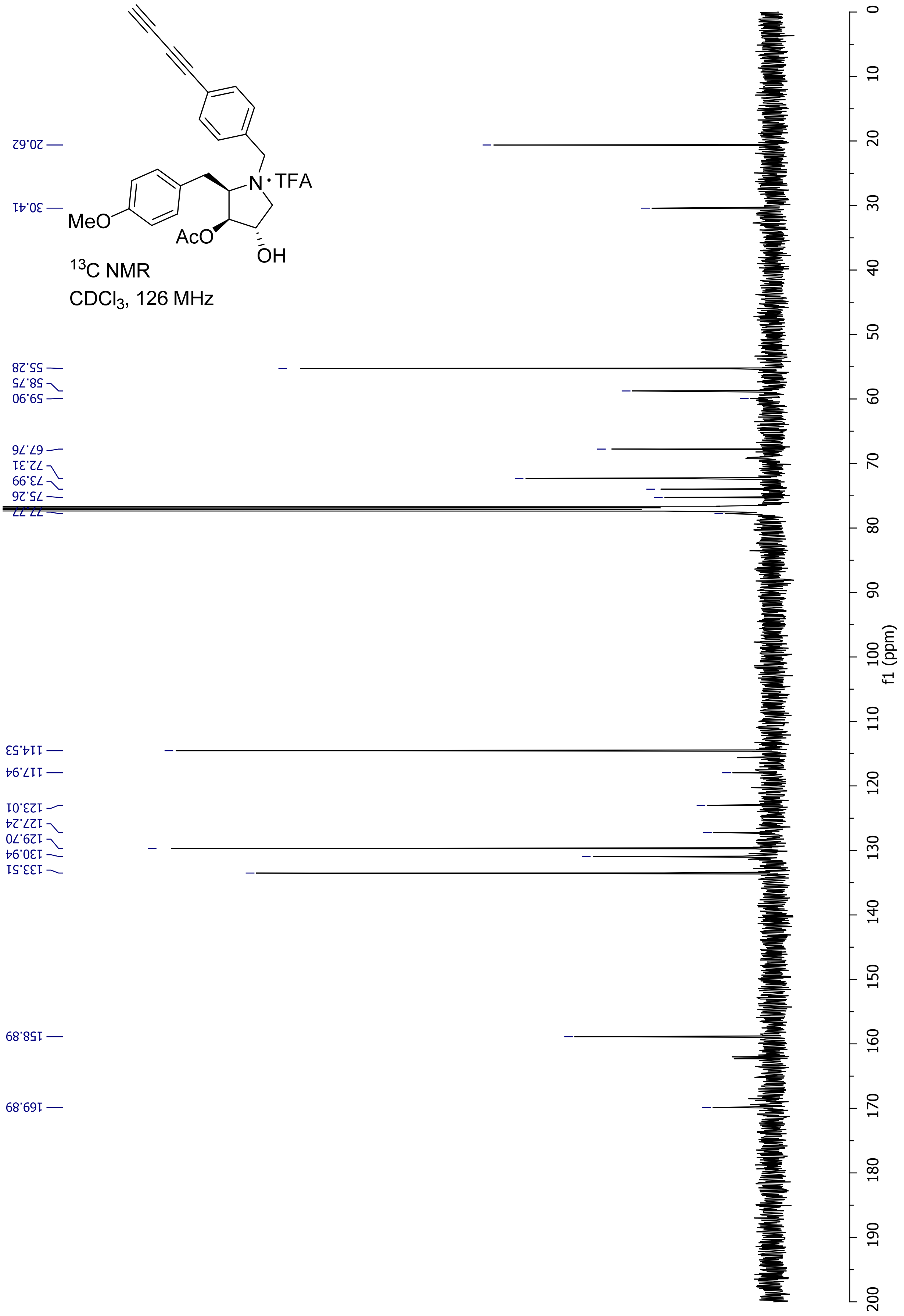
MeO
AcO
OH
N · TFA
13C NMR
CDCl3, 126 MHz
20.62
30.41
55.28
58.75
59.90
67.76
72.31
73.99
75.26
77.27
114.53
117.94
123.01
127.24
129.70
130.94
133.51
158.89
169.89
f1 (ppm)

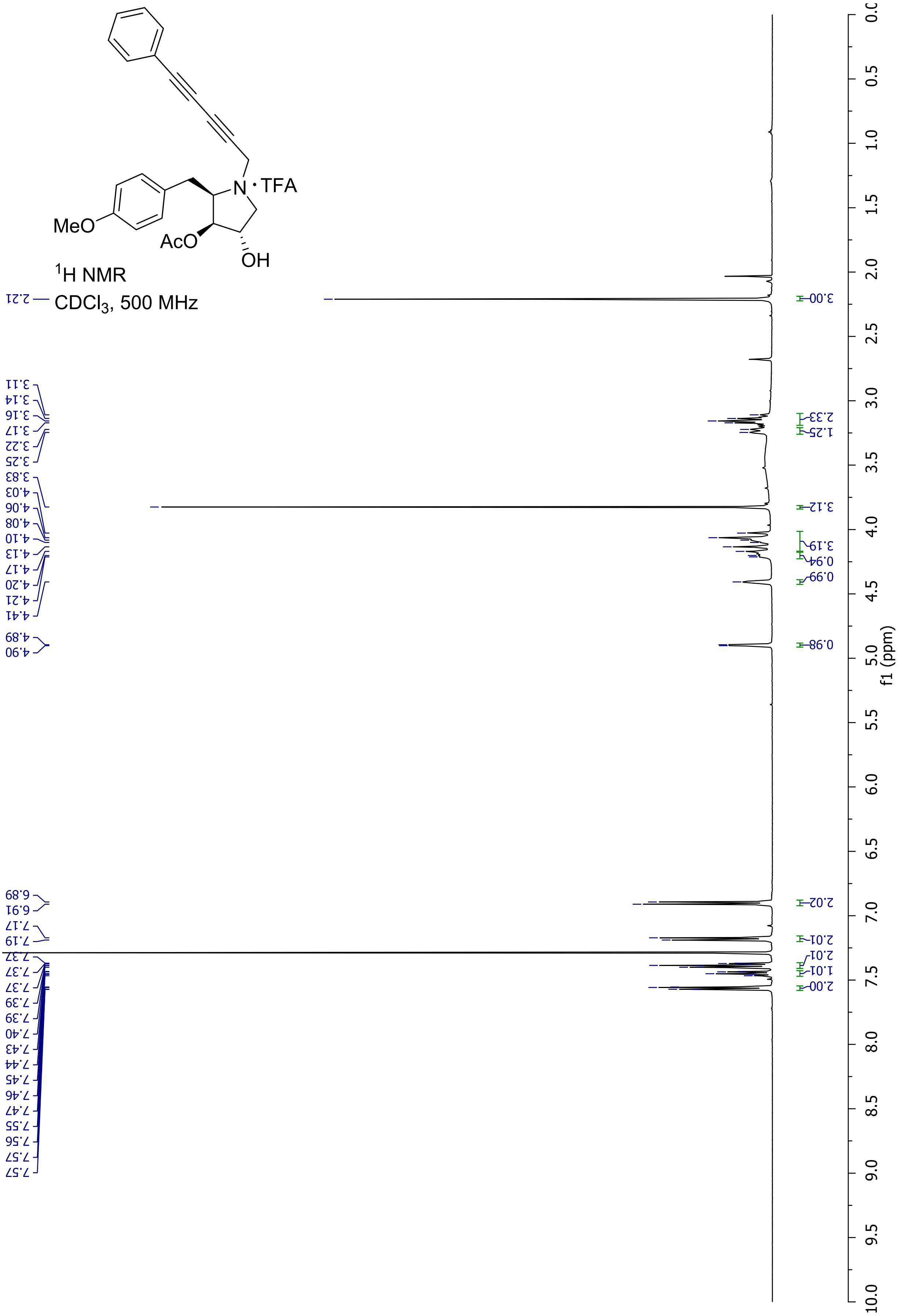

MeO
AcO
OH
N · TFA
^{1}H NMR
CDCl$_3$, 500 MHz
2.21
3.11 3.14 3.16 3.17 3.22 3.25
3.83 4.03 4.06 4.08 4.10 4.13 4.17 4.20 4.21 4.41
4.89 4.90
6.89 6.91 7.17 7.19 7.32 7.37 7.37 7.39 7.39 7.40 7.43 7.44 7.45 7.46 7.47 7.55 7.56 7.57 7.57
3.00
2.33
1.25
3.12
3.19
0.94
0.99
0.98
2.02
2.01
2.01
1.01
2.00
f1 (ppm)

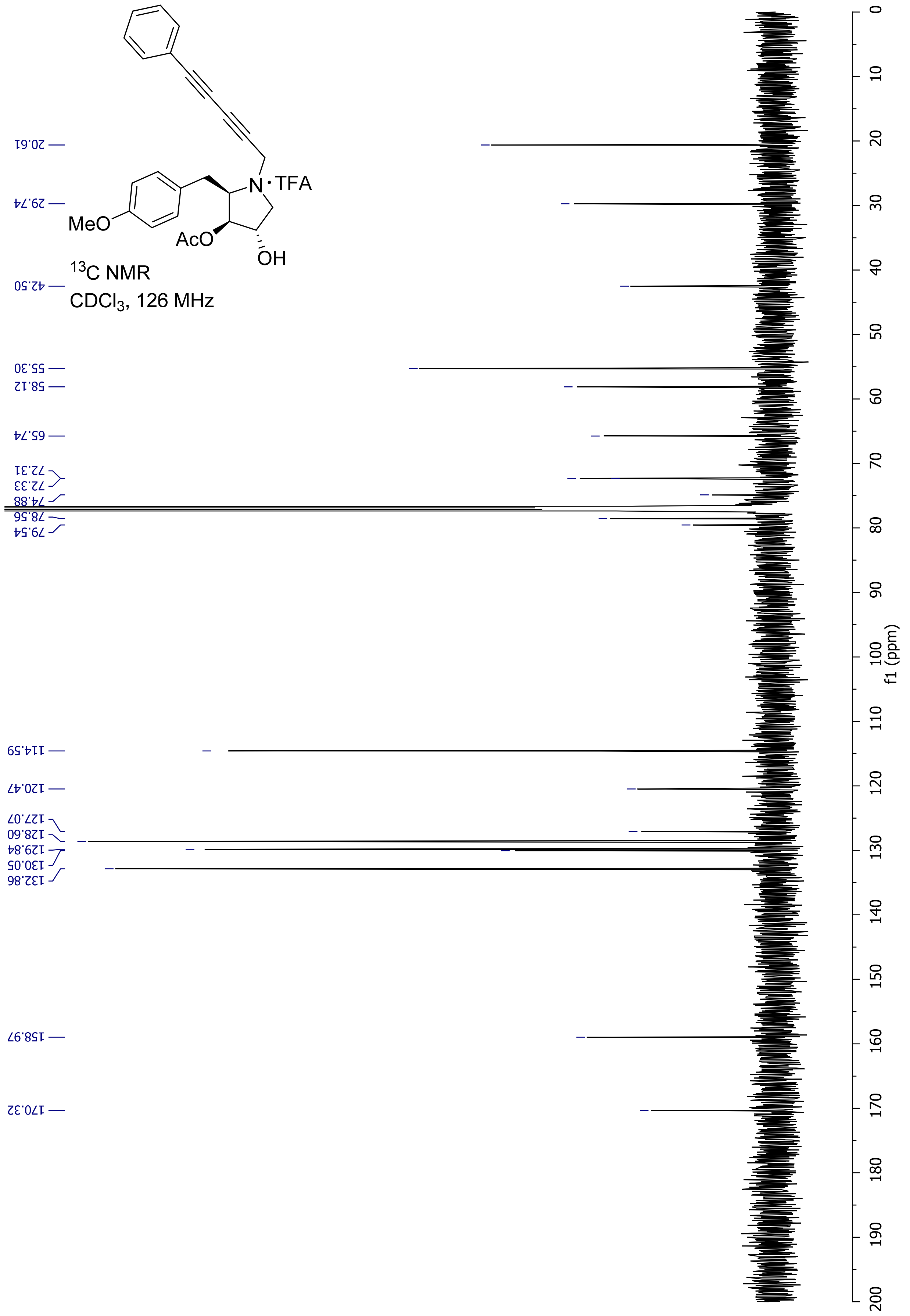
N·TFA
MeO
AcO
OH
13C NMR
CDCl3, 126 MHz
20.61
29.74
42.50
55.30
58.12
65.74
72.31
72.33
74.88
78.56
79.54
114.59
120.47
127.07
128.60
129.84
130.05
132.86
158.97
170.32
f1 (ppm)

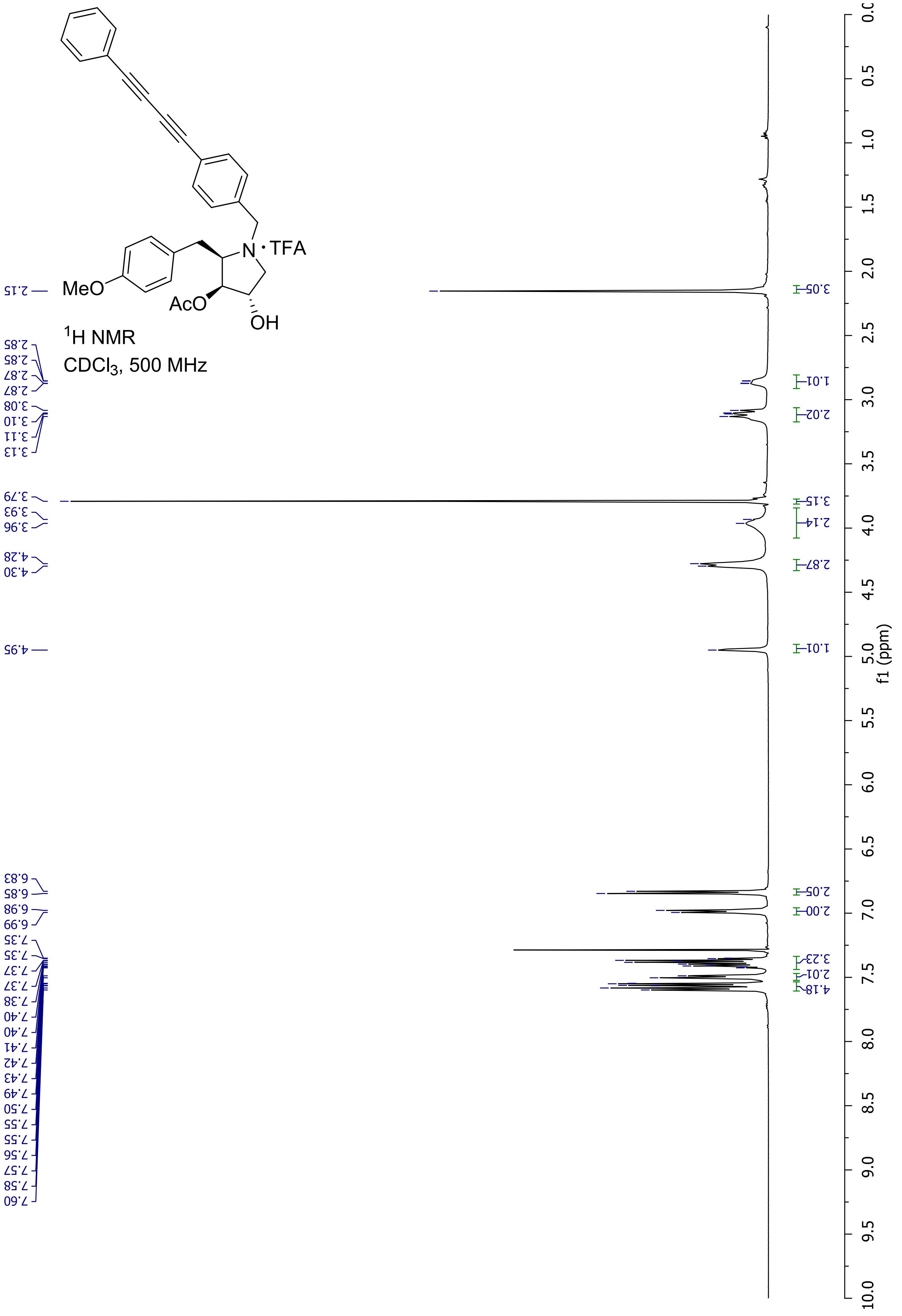

MeO
AcO
OH
N · TFA
1H NMR
CDCl3, 500 MHz
f1 (ppm)

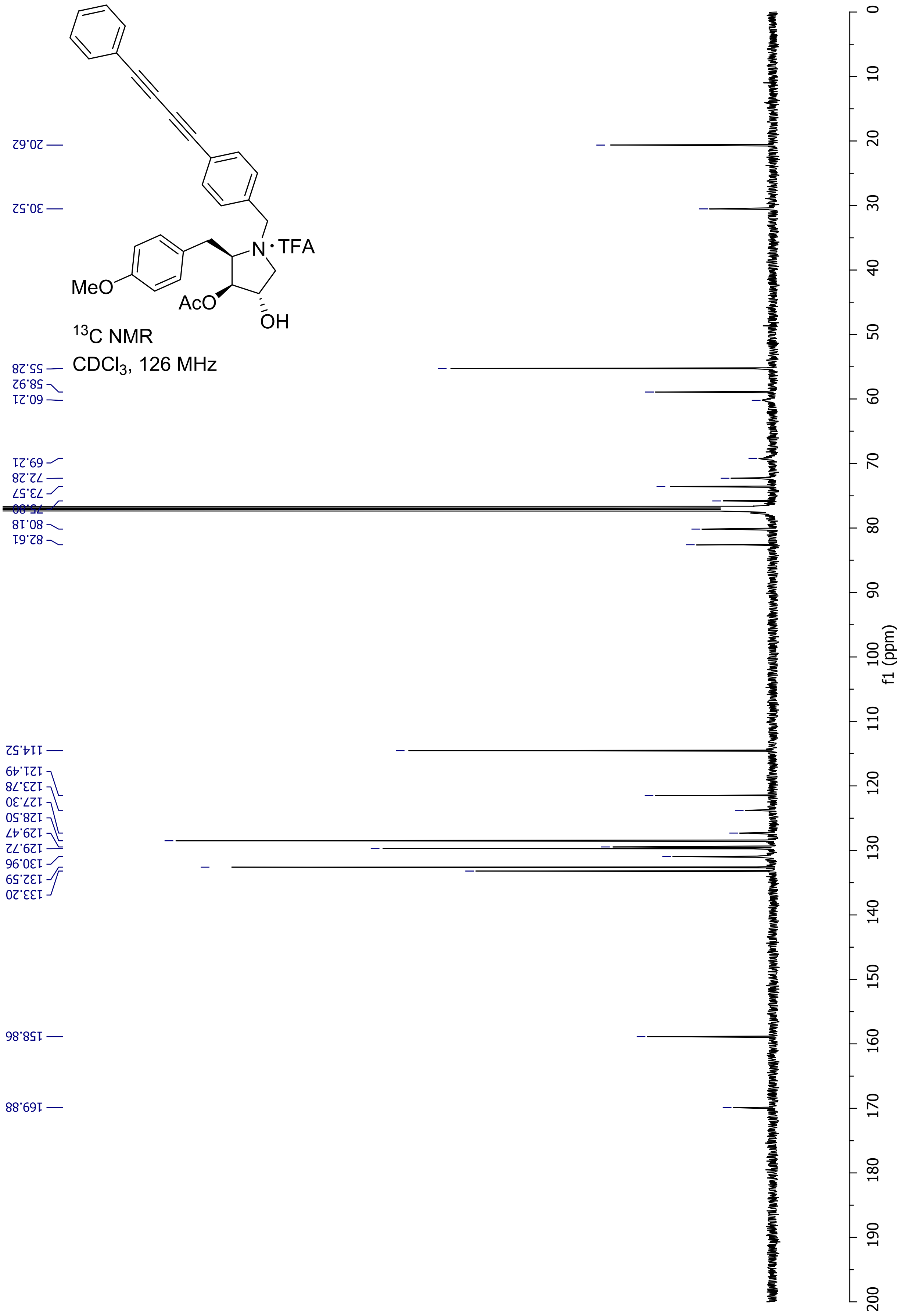

MeO
AcO
OH
N
· TFA
^{13}C NMR
CDCl$_3$, 126 MHz
20.62
30.52
55.28
58.92
60.21
69.21
72.28
73.57
80.18
82.61
114.52
121.49
123.78
127.30
128.50
129.47
129.72
130.96
132.59
133.20
158.86
169.88
f1 (ppm)

Optimised geometries for compounds **3b – i** and EdU **1** reported in standard orientation.

Compound **3b**

Center Number	Atomic Number	Atomic Type	Coordinates (Angstroms) X	Y	Z
1	7	0	-0.734458	0.019048	-0.315890
2	6	0	0.313091	-0.749856	0.346684
3	6	0	1.659521	-0.258487	0.012313
4	7	0	2.725570	0.128507	-0.232204
5	1	0	0.221497	-0.755436	1.451740
6	1	0	0.248247	-1.793650	0.018611
7	6	0	-0.767810	1.407920	0.133985
8	6	0	-2.028421	-0.633888	-0.155670
9	1	0	-2.358687	-0.702430	0.898480
10	1	0	-1.989625	-1.646617	-0.569082
11	1	0	-2.784963	-0.071902	-0.710056
12	1	0	-1.525026	1.951952	-0.437096
13	1	0	-1.007331	1.508945	1.209557
14	1	0	0.199817	1.882118	-0.049365

Compound **3c**

Center Number	Atomic Number	Atomic Type	Coordinates (Angstroms) X	Y	Z
1	7	0	-0.767768	0.026340	-0.327453
2	6	0	0.277283	-0.765840	0.326502
3	6	0	1.632731	-0.291730	0.032283
4	6	0	2.758672	0.085260	-0.186153
5	1	0	3.750362	0.415941	-0.391754
6	1	0	0.144504	-0.799805	1.428299
7	1	0	0.183575	-1.801230	-0.024993
8	6	0	-0.781121	1.408745	0.136043
9	6	0	-2.068516	-0.605670	-0.157562
10	1	0	-2.388366	-0.678099	0.900767
11	1	0	-2.050200	-1.617216	-0.576164
12	1	0	-2.825977	-0.030151	-0.697916
13	1	0	-1.528160	1.973716	-0.429597
14	1	0	-1.022327	1.503315	1.213072
15	1	0	0.196675	1.864557	-0.036221

Compound **3d**

Center Number	Atomic Number	Atomic Type	Coordinates (Angstroms) X	Y	Z
1	7	0	-1.392028	0.057378	-0.334655
2	6	0	-0.447850	-0.837083	0.344648
3	6	0	0.955656	-0.463455	0.146229
4	6	0	2.120160	-0.168431	0.006758
5	6	0	3.525513	0.183158	-0.175691
6	1	0	-0.652795	-0.897533	1.434543
7	1	0	-0.607190	-1.848581	-0.050995
8	6	0	-1.320897	1.421005	0.174688
9	6	0	-2.745833	-0.471159	-0.252143
10	1	0	-3.126560	-0.548533	0.785792
11	1	0	-2.785498	-1.468656	-0.702101
12	1	0	-3.426196	0.178875	-0.810630
13	1	0	-1.988051	2.062607	-0.409240
14	1	0	-1.612802	1.503112	1.240510
15	1	0	-0.301068	1.798584	0.070852
16	1	0	3.657016	0.848379	-1.036357
17	1	0	4.137220	-0.708781	-0.351827
18	1	0	3.929622	0.694676	0.705101

Compound **3e**

Center Number	Atomic Number	Atomic Type	Coordinates (Angstroms) X	Y	Z
1	7	0	1.743369	0.088101	-0.341381
2	6	0	0.851697	-0.851133	0.344060
3	6	0	-0.565912	-0.531043	0.175870
4	6	0	-1.748950	-0.277082	0.060645
5	6	0	-3.081257	0.004226	-0.071134
6	6	0	-4.262749	0.254405	-0.186994
7	6	0	1.639722	1.439034	0.198637
8	6	0	3.116762	-0.396923	-0.310218
9	1	0	2.272599	2.112049	-0.387209
10	1	0	0.607299	1.788793	0.123557
11	1	0	1.952840	1.508386	1.258658
12	1	0	3.175704	-1.382550	-0.783182
13	1	0	3.530632	-0.481379	0.713691
14	1	0	3.755788	0.287765	-0.875389
15	1	0	1.070778	-0.919510	1.430810
16	1	0	1.035101	-1.852197	-0.067659
17	1	0	-5.300200	0.473039	-0.288809

Compound **3f** (note the CD_3 group in compound **3f** was modelled as a CH_3 group).

Center Number	Atomic Number	Atomic Type	Coordinates (Angstroms) X	Y	Z
1	7	0	2.448883	0.130785	-0.355908
2	6	0	1.610169	-0.865899	0.317498
3	6	0	0.176990	-0.601446	0.191101
4	6	0	-1.019035	-0.395767	0.112814
5	6	0	-2.364893	-0.169503	0.021800
6	6	0	-3.561139	0.031825	-0.058714
7	6	0	2.294290	1.460356	0.222479
8	6	0	3.842372	-0.292795	-0.356710
9	1	0	2.885420	2.177552	-0.354801
10	1	0	1.245621	1.763349	0.174825
11	1	0	2.623024	1.515620	1.278831
12	1	0	3.937830	-1.262356	-0.856363
13	1	0	4.274909	-0.385997	0.658998
14	1	0	4.442802	0.434073	-0.911695
15	1	0	1.859959	-0.957681	1.396078
16	1	0	1.828900	-1.844584	-0.129685
17	6	0	-4.993769	0.271936	-0.154556
18	1	0	-5.543572	-0.317460	0.588139
19	1	0	-5.376746	0.001618	-1.145239
20	1	0	-5.230234	1.328138	0.017994

Compound **3g**

Center Number	Atomic Number	Atomic Type	Coordinates (Angstroms) X	Y	Z
1	6	0	1.484532	0.153765	-0.128905
2	6	0	2.895098	-0.005048	-0.036238
3	6	0	4.104613	-0.141372	0.043617
4	6	0	5.457705	-0.293958	0.132962
5	6	0	6.661833	-0.429750	0.212668
6	6	0	0.660936	-0.930802	-0.496260
7	6	0	-0.716072	-0.770711	-0.585674
8	6	0	-1.316815	0.466110	-0.309944
9	6	0	-0.499488	1.539897	0.061943
10	6	0	0.881639	1.395449	0.149696
11	1	0	7.717864	-0.549104	0.282449
12	6	0	-2.815067	0.647080	-0.463765
13	7	0	-3.589291	-0.485779	0.039252
14	6	0	-4.967655	-0.447127	-0.431861
15	6	0	-3.530831	-0.587380	1.493502
16	1	0	-3.991992	0.279904	2.004370
17	1	0	-2.490991	-0.660805	1.821372
18	1	0	-4.055172	-1.490887	1.819373
19	1	0	-5.530120	0.435978	-0.071939
20	1	0	-5.496614	-1.342109	-0.089781
21	1	0	-4.987956	-0.438512	-1.526243
22	1	0	-3.045112	0.748767	-1.532989
23	1	0	-3.117984	1.603182	0.007751
24	1	0	-1.350203	-1.608438	-0.857569
25	1	0	-0.950776	2.502880	0.287326
26	1	0	1.504697	2.234830	0.440275
27	1	0	1.116821	-1.892151	-0.709603

Compound **3h**

Center Number	Atomic Number	Atomic Type	Coordinates (Angstroms) X	Y	Z
1	7	0	-4.646655	0.153748	-0.404009
2	6	0	-3.878351	-0.820234	0.377146
3	6	0	-2.431145	-0.634692	0.275514
4	6	0	-1.223538	-0.494856	0.217317
5	6	0	0.130318	-0.344823	0.149261
6	6	0	1.341520	-0.210944	0.088250
7	1	0	-4.129916	-1.822433	0.005927
8	1	0	-4.162651	-0.809512	1.451034
9	6	0	-4.455154	1.516943	0.077383
10	6	0	-6.056790	-0.210923	-0.428709
11	1	0	-6.603158	0.495813	-1.060153
12	1	0	-6.176816	-1.210831	-0.858182
13	1	0	-6.530316	-0.208079	0.572801
14	6	0	2.754731	-0.058537	0.016411
15	6	0	3.340651	1.222737	0.066347
16	6	0	4.723058	1.365559	-0.006038
17	6	0	5.542769	0.240909	-0.127020
18	6	0	4.971872	-1.033098	-0.176428
19	6	0	3.590583	-1.187175	-0.106561
20	1	0	3.142711	-2.174575	-0.145888
21	1	0	5.605576	-1.909968	-0.270543
22	1	0	6.621018	0.356682	-0.182624
23	1	0	5.162793	2.357756	0.032655
24	1	0	2.700076	2.093286	0.160917
25	1	0	-3.393172	1.772585	0.049610
26	1	0	-4.821051	1.667160	1.111934
27	1	0	-4.991663	2.210682	-0.576665

Compound **3i**

Center Number	Atomic Number	Atomic Type	Coordinates (Angstroms) X	Y	Z
1	6	0	-1.315577	0.256791	-0.253756
2	6	0	0.102430	0.194002	-0.184730
3	6	0	1.321562	0.136091	-0.129048
4	6	0	2.678051	0.070319	-0.068597
5	6	0	3.897006	0.010016	-0.015175
6	6	0	-2.084959	-0.922580	-0.347723
7	6	0	-3.470461	-0.857154	-0.419874
8	6	0	-4.135809	0.377372	-0.397025
9	6	0	-3.373221	1.546765	-0.294047
10	6	0	-1.984414	1.496473	-0.226808
11	6	0	5.315475	-0.063999	0.045713
12	6	0	6.083557	1.090020	0.305092
13	6	0	7.471530	1.010723	0.363626
14	6	0	8.117246	-0.212249	0.165926
15	6	0	7.365588	-1.361306	-0.091903
16	6	0	5.977147	-1.293657	-0.152558
17	6	0	-5.644785	0.442378	-0.535417
18	7	0	-6.341919	-0.583998	0.237408
19	6	0	-7.730790	-0.721703	-0.180849
20	6	0	-6.243209	-0.349965	1.673983
21	1	0	-6.743122	0.585148	1.992799
22	1	0	-5.193237	-0.292310	1.971200
23	1	0	-6.704863	-1.182158	2.214256
24	1	0	-8.333520	0.191533	-0.011332
25	1	0	-8.200973	-1.539754	0.373797
26	1	0	-7.777030	-0.964492	-1.247186
27	1	0	-5.905045	0.286370	-1.591170
28	1	0	-5.991810	1.464019	-0.281928
29	1	0	-4.060407	-1.766112	-0.481429
30	1	0	-3.874374	2.511101	-0.265512
31	1	0	-1.404625	2.410082	-0.146821
32	1	0	-1.579384	-1.882607	-0.365200
33	1	0	5.389318	-2.183329	-0.352625
34	1	0	5.577856	2.037610	0.458229
35	1	0	8.052149	1.906263	0.564220
36	1	0	9.200600	-0.269551	0.212369
37	1	0	7.863699	-2.313849	-0.246507

EdU **1**

Center Number	Atomic Number	Atomic Type	Coordinates (Angstroms) X	Y	Z
1	6	0	-3.465456	-0.156665	0.141031
2	6	0	-2.495357	0.949151	0.069546
3	6	0	-1.182516	0.663521	-0.174319
4	7	0	-0.708477	-0.608621	-0.349559
5	6	0	-1.547545	-1.725838	-0.300667
6	7	0	-2.872801	-1.418918	-0.067171
7	8	0	-4.660692	-0.064356	0.349249
8	8	0	-1.130455	-2.864252	-0.449351
9	6	0	-2.944260	2.286255	0.242697
10	6	0	0.730596	-0.893789	-0.597338
11	8	0	1.377160	0.293538	-1.031385
12	6	0	2.468053	0.606272	-0.148842
13	6	0	2.851185	-0.732291	0.502545
14	6	0	1.478651	-1.388847	0.651891
15	6	0	3.584842	1.247264	-0.956472
16	8	0	4.671367	1.409807	-0.043620
17	8	0	3.498049	-0.593251	1.745262
18	1	0	3.258898	2.209279	-1.375194
19	1	0	3.848241	0.581258	-1.791028
20	1	0	1.006679	-1.000883	1.560312
21	1	0	1.500216	-2.477110	0.709650
22	1	0	-0.438225	1.442703	-0.265904
23	1	0	-3.499896	-2.213420	-0.024743
24	1	0	0.746835	-1.631428	-1.402974
25	1	0	3.468511	-1.306020	-0.209650
26	1	0	2.142207	1.295376	0.644590
27	6	0	-3.319951	3.426318	0.391604
28	1	0	-3.665182	4.425265	0.524165
29	1	0	4.279920	-0.044872	1.576336
30	1	0	5.427849	1.772633	-0.519746
